# Salts of boronium (+3) ions with unprecedented hydrolytic stability. Design, synthesis, single-crystal X-ray structures, electrochemistry, and thermophysical properties†

**DOI:** 10.1039/d5ra02301g

**Published:** 2025-06-12

**Authors:** Margaret E. Crowley, Christopher D. Stachurski, James H. Davis, Matthias Zeller, Gabriel A. Merchant, E. A. Salter, A. Wierzbicki, Richard A. O'Brien, Paul C. Trulove, David P. Durkin

**Affiliations:** a Department of Chemistry, University of South Alabama Mobile AL 36688 USA jdavis@southalabama.edu; b Department of Chemistry, United States Naval Academy Annapolis MD 21402 USA; c Department of Chemistry, Purdue University West Lafayette Indiana 47907 USA

## Abstract

Boron cations (boronium ions) of any charge – 1+, 2+, or 3+ – are demonstrably among the least well-known and studied family of monoboron species. Those of 3+ charge are especially few in number, and scant information is available about them. This lack of information is likely rooted in the relatively poor stability – especially towards water – of known exemplars. Now, by utilizing strongly donating 4-*N*,*N*-dialkylaminopyridines as ligands to the boron center we have succeeded in preparing salts indefinitely stable in water at pH values from 1 to 14. Further, we have acquired multinuclear NMR spectra on each of the new species and for the first time evaluated their thermal characteristics (DSC and TGA), finding that they are quite robust. In addition, the electrochemical behaviour of the new salts was evaluated using cyclic voltammetry. The structures of several of the new materials have been determined by single-crystal X-ray crystallography, and detailed computational modelling has been undertaken to provide additional insight into the experimental data.

## Abstract 2

Boron trications are rare and tend to be unstable, especially in water. We show herein that they can be made highly water-stable by using strongly electron-donating 4-(dialkylamino)pyridine ligands to support the boron centre.

## Graphical abstract

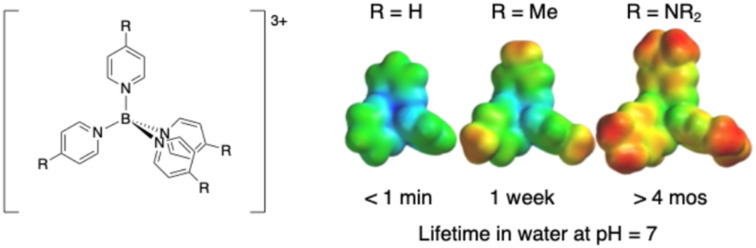

## Introduction

Boronium (+3) ions are rare and their chemistry is scarcely explored. They, like their boronium (+1) and (+2) counterparts, consist of a central boron atom in a tetrahedral coordination environment. However, unlike the (+1) and (+2) ions, all four ligands in the (+3) ion are neutral, N-donor Lewis bases; the latter are almost always pyridine or a simple alkyl pyridine variant thereof.^1^ They are particularly interesting for several reasons. First, they fill a representational gap in the fundamental chemistry of coordination complexes. To our knowledge, there are no other types of tetrahedral, 3+ complexes of a transition-metal or main-group element supported only by neutral ligands although there are square-planar Au(iii) complexes with four pyridine ligands, and other main-group trications, albeit with structures which are not tetrahedral.^2,3^ Second, the parent Py_4_B^3+^ (Py = pyridine) ion is evocative of the tetraphenylphosphonium ion, Ph_4_P^+^, which is valued for its propensity to form easily crystallizable salts of anions of interest, and which has seen widespread use as a probe ion in studies of mitochondrial membrane potentials.^4,5^ Finally, the combination of large size, tetrahedral geometry, structural rigidity, and high charge on these boroniums ions may make them useful for engineering hybrid organic–inorganic solids, provided issues with their hydrolytic instability can be overcome.^6^ The latter is a concern of practical importance, a current case-in-point being the application-hampering water sensitivity of many perovskites (including hybrid organic–inorganic exemplars) being developed as solar cell materials.^7^

**Fig. 1:**
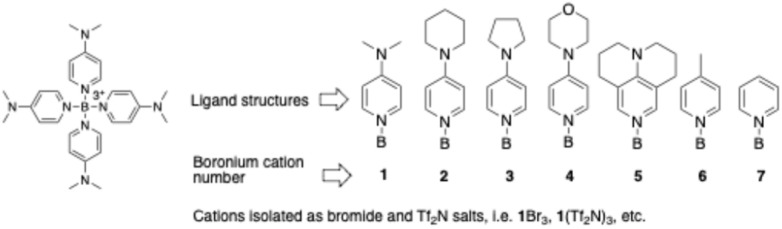
Left: Basic structure of the Py_4_B^3+^ cations. Right: Structures (1–5) of the 4-alkylamino pyridine ligands of the new cations, followed by those of the Ryschkewitsch (6) and Bohl (7) cations.

In 1970 Bohl and Galloway isolated the parent Py_4_B^3+^ ion as an iodide salt which they reported to be exquisitely sensitive to water, rapidly decomposing upon addition to an aqueous solution of KPF_6_ in an attempt at anion exchange. Consequently, the sole published characterization data for it consists of elemental analysis (C, H, N, and I) of the initially isolated product.^8^ Contemporaneously, Ryschkewitsch and co-workers found that a derivative cation bearing four 4-methylpyridine ligands was somewhat more stable, albeit on a pH-dependent basis.^9^ Specifically, this cation was stable for ∼1 week in water at pH = 1, moderately stable for 24 h at pH = 7, decomposed in ∼1 h at pH = 11 and in ∼1 min at pH = 13.6. The improved stability of the latter cation *versus* that of Py_4_B^3+^ was attributed to the 4-methyl group increasing electron density at the pyridyl nitrogen. Against this background, and given our ongoing work with boronium ions, we were interested in attempting to further improve the aqueous stability of boronium (+3) ions so that their chemistry might be more thoroughly evaluated, including making determinations of the thermochemical and electrochemical characteristics of this cation class. It was our hypothesis that improving the stability of these cations might be accomplished by using as ligands the powerful nucleophilic N-donor DMAP (4-dimethylamino pyridine) and variants thereof (Fig. 1).^10^

## Experimental

### Representative synthesis

**Scheme 1:**
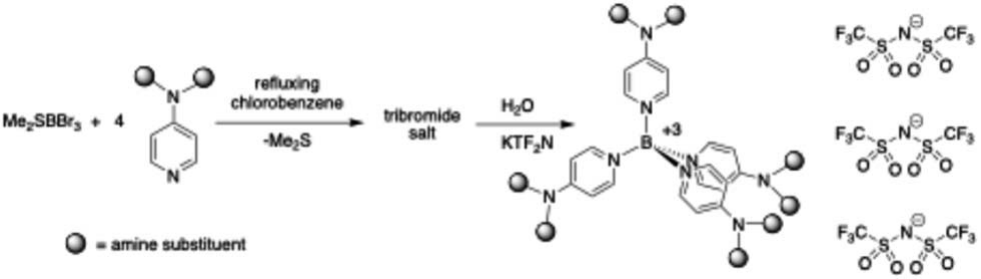
Synthesis of the Br^−^ and Tf_2_N^−^ salts of cations 1–5.

The bromide salts of the product cations routinely contained trace amounts (and similar from cation to cation) of an unidentified but apparently boron-containing impurity which we were unable to separate despite repeated attempts. Consequently, very small resonances for this substance appear in the ^1^H, ^13^C, and ^10^B NMR spectra of the bromide salts. In a well-ventilated fume hood, a 500 mL, two-neck round bottomed flask was charged with a magnetic stirbar and 200 mL of reagent grade chlorobenzene. It was then fitted with a heating mantle and an air-cooled condenser which was loosely plugged at the top. 4-*N*,*N*-Dimethylamino pyridine (DMAP; 15.6 g, 0.128 mol) was added through the side neck, and the resulting pale ivory solution brought to reflux. Through the side neck were then added, portion-wise, 10.0 g of solid Me_2_SBBr_3_ (note: fumes in air). Upon mixing there was vigorous off-gassing of the dimethylsulfide as the boron reagent dissolved (see ESI† for important safety notes). Once addition of the latter was complete, the side neck was stoppered and reflux continued for 12 h, during which time the orange-yellow solution faded in color and a voluminous quantity of white solid separated. The suspension was vacuum filtered while still hot. The white, granular product was then washed successively with fresh chlorobenzene, toluene, and diethyl ether. Yield of 1Br_3_: 21.2 g (89.6%). A portion of the product was used in the subsequent anion exchange step, as well as in pH stability studies of the cation (*vide infra*). Bromide salts of cations 2–5 were prepared in like fashion (Scheme 1).

A 500 mL Erlenmeyer flask was charged with 200 mL of hot water and a magnetic stirbar. While stirring, 15.0 g (0.020 mol) of the preceding bromide salt was added. It dissolved quickly, producing a colorless solution. Separately, 20.0 g (0.062 mol) of KTf_2_N [potassium bis(trifluoromethanesulfonyl)imide] was likewise dissolved in hot water, and the resulting solution slowly added to that of the bromide salt. Upon mixing, a copious amount of white solid precipitated. Stirring was continued for an hour after which time the product [1(Tf_2_N)_3_] was separated by vacuum filtration, washed with water, and dried *in vacuo* (25.7 g, 96%). The Tf_2_N^−^ salts of cations 2–5 were prepared in the same way (Scheme 1).

### NMR spectroscopy

All NMR spectra – ^1^H (500 MHz), ^13^C (125 MHz), and ^10^B (54 MHz) – were collected using a JEOL JNM-ECA series, 500 MHz FT-NMR spectrometer. For the bromide salts of compounds 1–2, the ^1^H spectra are reported in ppm referenced to residual HDO/H_2_O the D_2_O solvent (*δ* 4.79). For those of 3–5, the ^1^H and ^13^C spectra are reported referenced to methanol-d_4_ (*δ* 4.79 and *δ* 49.0, respectively). After subsequent ion exchange from bromide salts of cations 1–5 to salts of the Tf_2_N^−^ anion, all were found to be soluble in acetone. NMR analysis of the same three nuclei was performed in acetone-d_6_ referenced at *δ* 2.05 in ^1^H and *δ* 206.26 for ^13^C. With the addition of the fluorinated anion, ^19^F (471 MHz) spectra were also acquired. Note that ^10^B spectra were acquired rather than (the more commonly used) ^11^B; in our experience with boronium ions, the former tends to produce spectra with narrower line widths and not requiring baseline correction.

### Thermogravimetric analysis

The thermal stability of the Tf_2_N^−^ salts of trications 1–5 were evaluated using thermal gravimetric analysis (TGA; TA Instruments TGA 5500). Samples were loaded into platinum pans and heated under nitrogen from 20–800 °C at a heating rate of 10 °C min^−1^. The decomposition temperature (*T*_5_) was taken as the point at which 5 wt% of the initial sample mass was lost.

### Differential scanning calorimetry

Thermal phase transitions were measured using differential scanning calorimetry (DSC; TA Instruments Q2000) equipped with liquid nitrogen cooling. Samples were sealed in hermetic aluminum pans under nitrogen and cycled from room temperature (RT) to −150 °C, then 350 °C at 10 °C min^−1^ under a helium purge at a flow rate of 25 mL min^−1^.

### Electrochemistry

All electrochemical experiments were conducted in a nitrogen filled glovebox to control for ambient water exposure (<1 ppm H_2_O). Samples of the Tf_2_N^−^ salts were prepared in acetonitrile to predetermined concentrations (25- and 35 mM solutions of trications 2 and 3 (respectively) and 50 mM concentrations for the remaining salts). A standard solution of 50 mM tetrabutylammonium hexafluorophosphate was used to establish a baseline behavior for acetonitrile. Cyclic voltammetry (CV) was performed using a Biologic SP-200 potentiostat. All samples were analyzed in a standard three-electrode cell comprised of a glassy carbon working electrode (EDAQ, surface area = 7.8 × 10^−3^ cm^2^), a platinum mesh counter electrode, and a silver wire serving as a quasi Ag/Ag^+^ reference electrode. Potentials were adjusted to the *E*_1/2_ of the ferrocene/ferrocenium (Fc/Fc^+^) couple for each cell following analysis of the boronium salt. All electrodes were held in the same compartment, not separated by any membrane or frit. For all standard cyclic voltammograms, the working electrode was held at the open circuit potential (*E*_oc_) before sweeping between the switching potentials at a scan rate of 50 mV s^−1^. The working electrode was polished between scans using a series of alumina slurries (6, 1, 0.25 μm) and cleaned with a microfiber polishing pad.

## Results and discussion

Five new boronium (+3) cations were prepared (see structures 1–5, Fig. 1) as bromide salts. The synthetic procedure for the salts was a modification of that used by Bohl and Galloway as well as Ryschkewitsch and his co-workers for the synthesis of L_4_B^3+^ cations (L = pyridine or 4-methylpyridine); in short, four molar equivalents of the pyridine of interest were combined with commercially available Me_2_SBBr_3_ (Galloway used Me_3_NBI_3,_ while Ryschkewitsch used Me_3_NBBr_3_) in refluxing chlorobenzene, the boronium bromide salt then precipitated in essentially quantitative yield. The bromide salts are all white solids. Those of cations 1, 4, and 5 are non-hygroscopic while those of 2 and 3 absorb atmospheric moisture. The Tf_2_N^−^ salts of each of the cations were prepared by an aqueous anion exchange between the respective bromide salt and KTf_2_N. Unlike the experience of Bohl and Galloway when they attempted an anion exchange between the iodide salt of Py_4_B^3+^ and KPF_6_, no sensitivity to water was observed for the present cations.

D_2_O solutions were prepared having pH (pD) values ranging monotonically from 7 to 14. The bromide salt of the parent DMAP boronium, 1, was dissolved in these solutions and ^1^H-, ^13^C- and ^10^B-NMR spectra were acquired (*T* = 20 °C) five minutes after dissolution, after one week, after two weeks, and finally after four months (note: the Tf_2_N^−^ salt of the cation is water-insoluble; hence, to gauge the cation's stability towards water, the soluble bromide salt was used). The outcome was clear. The spectra of 1 were unchanged at pHs 1–13 (acidic pH range experiments below) over any length of time; some evidence of decomposition (extra peaks) was observed at pH = 14 after four months. We then undertook a similar study on 2, (selected as a representative of those salts bearing more lipophilic R_2_N^−^ groups). The spectra of 2 showed no changes at benchmark pH values of 1, 7, or 13 after five minutes, after one week, or after four months. However, (apparent) decomposition products were visible even after one week at pH = 14.

We also considered that using these *N*,*N*-dialkylamino pyridine ligands might create a sensitivity on the part of the boroniums towards acidic media due to the lone electron pair on the dialkylamine groups. We note that protonation of the Me_2_N^−^ group of a DMAP ligand coordinated to a molybdenum atom through the pyridyl nitrogen has been reported by Harman *et al.*^11^ Further, Forsythe and co-workers have shown that DMAP protonated at the pyridine nitrogen can be further protonated at the Me_2_N group in acidic media.^12^ Consequently, we thought it wise to extend our pH studies into the acidic domain. Accordingly, the bromide salts of 1 and 2 were dissolved in D_2_O having pH (pD) values varying monotonically from 1–7, with ^1^H-, ^13^C-, and ^10^B-NMR spectra acquired on each after five minutes, one week, two weeks, and four months. Here again, no changes were observed for either boronium at any of those pH values over any of those periods of time, indicating that the cations remained stable in the acidic pH domain as well. Indeed, the wholesale lack of any chemical shift changes also suggests that no protonation at the R_2_N^−^ substituents occurs in these ions, in contrast to the Harman and Forsythe cases (*vide supra*). We posit that the coordination of the present ligands to the B (+3) center is sufficiently depleting of electron density on the R_2_N^−^ nitrogen atoms to render them inert to protonation. Globally, these NMR studies provide clear evidence that at ambient temperature, boronium cations 1 and 2 – and by extension, we posit, 3–5 as well – are highly resistant to hydrolysis under conditions ranging from very acidic to very basic. This is in sharp contrast to previous reports detailing the aqueous stability of (Py)_4_B^3+^ (the Bohl cation) and (Pic)_4_B^3+^ (Pic = picoline, 4-methylpyridine, the Ryschkewitsch cation). Indeed, given the very well-known propensity for molecular monoboron compounds to react with water to form boric acid (
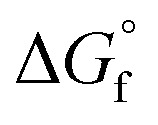
 = −1093 kJ mol^−1^), this degree of pH-independent stability towards water is impressive.

Seeking to gain insight into origins of the enhanced stability of cations 1–5, computational studies were also carried out. The cation geometries were optimized, and atomic natural bond order (NBO) partial charges of the new species were assigned using Gaussian 16.^13^ All structures were optimized under *S*_4_ symmetry using the wB97XD density functional method and the 6-31g(d) basis set, followed by evaluation of frequencies, then reoptimized using the cc-pvtz basis set. Subsequently, electrostatic potential maps were generated by Spartan'24 from single-point calculations.^14^ The partial charges on the B and N atoms in each cation are presented in Table 1. Additional computational details and coordinates of optimized structures are available in the ESI.†

**Table 1:** Computed partial charges for select atoms in cations 1–7, and the experimental B–N bond distances from crystal structures of 1–3, 5 & 6. Note that 6 and 7 are the Ryschkewitsch and Bohl cations, respectively

Computed partial charges								
Atom	1	2	3	4	5	6	7	
B	1.074	1.064	1.072	1.066	1.082	1.104	1.118	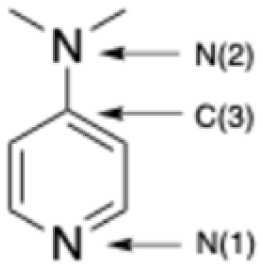
N(1)	−0.579	−0.582	−0.583	−0.581	−0.568	−0.543	−0.536	
C(3)	0.288	0.290	0.291	0.292	0.272	0.174	−0.035	
N(2)	−0.306	−0.315	−0.313	−0.318	−0.032	N/A	N/A	

**Experimental (upper) and computed (lower) B–N(1) distances (Å)**								
	1.599	1.561	1.556	N/A	1.561	1.585	N/A	
	1.566	1.564	1.566	1.565	1.565	1.576	1.580	

**Fig. 2:**
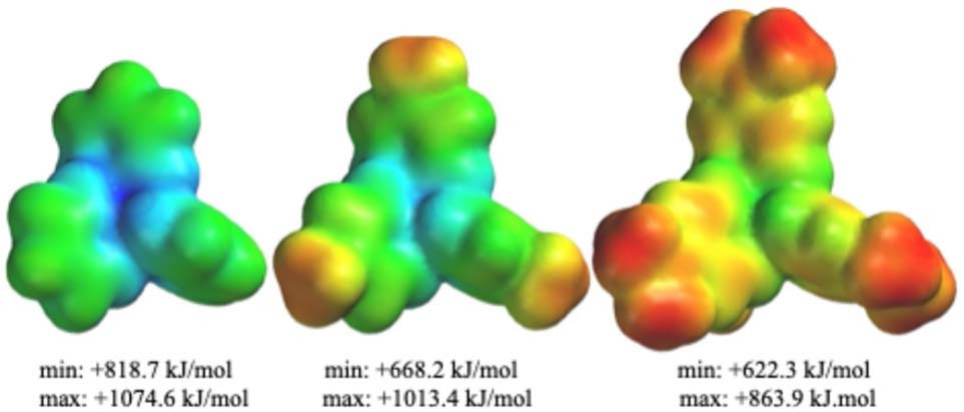
Electrostatic potential maps (Elstats) for (left to right) cations 7 (the Bohl cation), 6 (the Ryschkewitsch cation) and 1, parent cation of the present series. Note how the centroid of the structures becomes less blue [*e.g.*, less positive] from left to right, consistent with the boron center being provided more electron density by the coordinating pyridines. In addition, greater electron density in the π clouds of the pyridine rings from left to right is apparent. Common color scale: +622.3 (red) to +1074.6 (blue) kJ mol^−1^.

As is apparent from this data, the presence of a dialkylamine group in the pyridine 4-position enhances the partial negative charge on the pyridinyl nitrogen in 1–5 relative to those in cations 6 and 7. In turn, the *p*-methyl group on cation 6 enhances the negative charge on the pyridinyl nitrogen *versus* that on cation 7, as posited by Ryschkewitsch. But notably the 6*vs.*7 negative-charge difference of 0.007 is dwarfed by that of 0.043 which exists between that of the average value of cations 1–5*versus* cation 7. This substantiates our hypothesis that introducing an R_2_N^−^ substituent in the 4-position of pyridine would result in ligands better able to stabilize the high charge on a boronium (+3) center. Indeed, the computed negative charge on the pyridyl Ns progressively becomes more negative as the putative donor strength in the 4-position of the pyridine ring increases, and by the same token the positive charge on the boron decreases as the donor strength increases. These general trends can be visually appreciated by comparing the electrostatic potential maps for cations 1, 6, and 7 (Fig. 2). Consistent with the foregoing, the computed (Table 1) N–B bond distances in 1–5 are shorter than those in 6 and 7, suggesting stronger bonds between those atoms in the new set of cations.

**Fig. 3:**
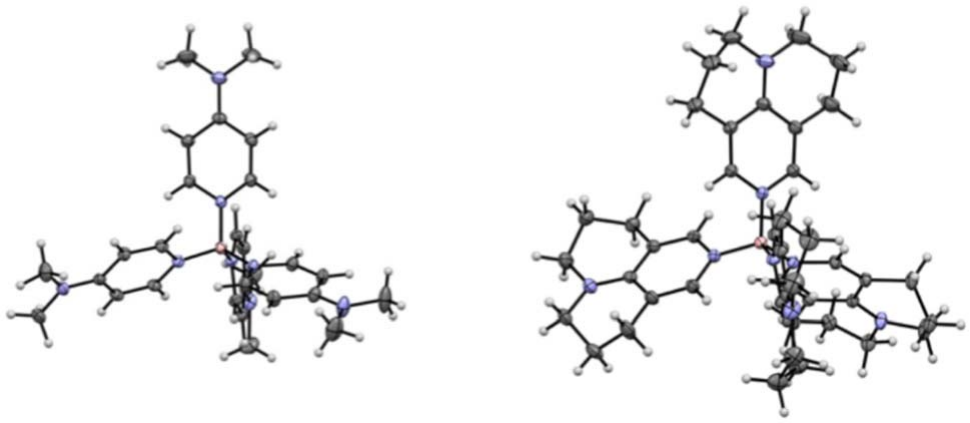
Structures of boronium (+3) cations 1 (left) and 5 (right). The associated Tf_2_N^−^ anions have been omitted for clarity. As the structure of 5 makes clear (when viewed in a space-filling version), the boron is capable of accommodating around it even four highly bulky ligands.

Colorless, X-ray quality single crystals of the Tf_2_N^−^ salts of cations 1, 2, 3 and 5 were obtained by recrystallization of the initial solid products from hot methanol or acetone-methanol mixtures, allowing us to acquire structures (Fig. 3 and Table 1) that enabled us to compare the B–N bond distances in the present boroniums with that in the (4-MePy)_4_B^3+^ (Ryschkewitsch) cation; note that years after the original Ryschkewitsch work, Vargas-Baca, Cowley, and co-workers successfully crystallized and obtained an X-ray structure on the bromide salt of the latter.^15^ In contrast, no comparison to the Bohl cation is possible since there is no published X-ray structure of any salt containing it.^8^ It also bears mention that while the Tf_2_N^−^ salt of cation 4 produced visually satisfactory crystals, they yielded hopelessly disordered structures even after multiple recrystallizations from different solvent media.

As shown in Table 1, the B–N bond distances in 1, 2, 3, and 5 are the same within experimental error, consistent with the five DMAP-family ligands having similar electron-donating strength to the boronium center. Further, these distances are significantly shorter than that between N and B in the Ryschkewitsch cation 6, validating the computational results (*vide supra*), and suggesting a weaker B–N bond in the latter. This is consistent with the longstanding understanding that amine groups are more “electron donating” (better *ortho*–*para* directors) than are alkyl groups. In accord with the foregoing, the C(3)–N(2) distances in 1, 2, 3, and 5 are such as to suggest that the capacity of their 4-amino groups to ‘push’ electron density into the pyridines were fundamentally the same. Again, complimenting these data are the computed charges (Table 1) for the boron and pyridine N atoms in boroniums 1–7. Note that the charges on the pyridine Ns in 1–4 are quite close, and that of 5 relatively close as well, but there is a sharp drop-off for those in 6 and 7. Even more significant, the positive charges on the Bs of 1–5 are close, but there is a noticeable increase for those in 6 and 7, the Ryschkewitsch and Bohl cations, respectively.

Thermal properties of the suite of the Tf_2_N^−^ salts of cations 1–5 were measured using both TGA and DSC. Temperatures (*T*_5_) were measured in duplicate to ensure consistency (Fig. S1†). Remarkably, the boronium (+3) cations exhibit thermal stabilities (Table 2) up to 278 °C, nearly 50 °C higher than boronium salts in which the cation contains at least one trialkylamine moiety. The salt of cation 5 exhibits the highest thermal stability (336 °C), on par with some of the highest reported thermal stabilities for both past reported boronium salts and other onium salts such as imidazolium, phosphonium, and pyrrolidinium cations.^16–22^ We speculate that the increase in *T*_5_ from cations 1–4 to cation 5 may indicate that the very large bulk of the latter leads to a delay in the onset of whatever mechanism is operative in its thermal decomposition.^23,24^

**Table 2:** All data is for Tf_2_N^−^ salts

Thermal characteristics		
Cation	*T*_5_ (^o^C)	*T*_m_ (^o^C)
1	291 ± 2	202
2	295 ± 3	149
3	278 ± 10	—
4	280 ± 2	—
5	336 ± 4	89

In addition to thermal stability, the phase behavior of salts such as the present boroniums is of interest to better understand how the charge distribution and chemical nature of the cation influence the overall properties of the material. The high charge on each cation, and consequentially higher number of associated anions, leads to salts with melting temperatures well above room temperature for the Tf_2_N^−^ salts of 1–5; this differs significantly from the characteristics of several known boronium (+1) Tf_2_N salts which are ionic liquids (having sub-100 °C *T*_m_). Across the board with 1–5, no phase transitions at low temperatures (*i.e.*, glass transitions) were observed. On the heating curves, the Tf_2_N^−^ salts of 1, 2, and 5 all exhibit clean melting behavior, at 202, 149, and 89 °C, respectively (Fig. S2†). We note that the *T*_m_ of 5(Tf_2_N)_3_ makes it ‘classifiable’ as an ionic liquid by the frequently used (but arbitrary) metric of being <100 °C.^24^ In addition to an initial solid–liquid transition, cation 5 exhibits a second endothermic peak at 198 °C, which appears to be an example of a high temperature liquid–liquid transition.^25^ Salts 3 and 4 also undergo endothermic transitions at elevated temperatures, though broader peaks are seen compared to those of 1, 2, and 5 (Fig. S2†). While less well-defined, two endothermic peaks can be distinguished from the heating curve of 3, much like 5, suggesting similar thermal behavior for the two samples.

Cyclic voltammetry (CV) was performed on cation 1 to determine a baseline electrochemical behavior for a boronium (+3) salt, something which to our knowledge has not previously been reported. Although our earlier studies on boronium salts focused on innately liquid materials, *i.e.*, ionic liquids, the high *T*_m_ values of the boronium (+3) salts prevented their direct study as neat electrolytes; accordingly, solutions in acetonitrile were used to investigate their electrochemical behavior, particularly with respect to electrochemical degradation or the buildup of solid–electrolyte interface (SEI) layers.^26,27^

**Fig. 4:**
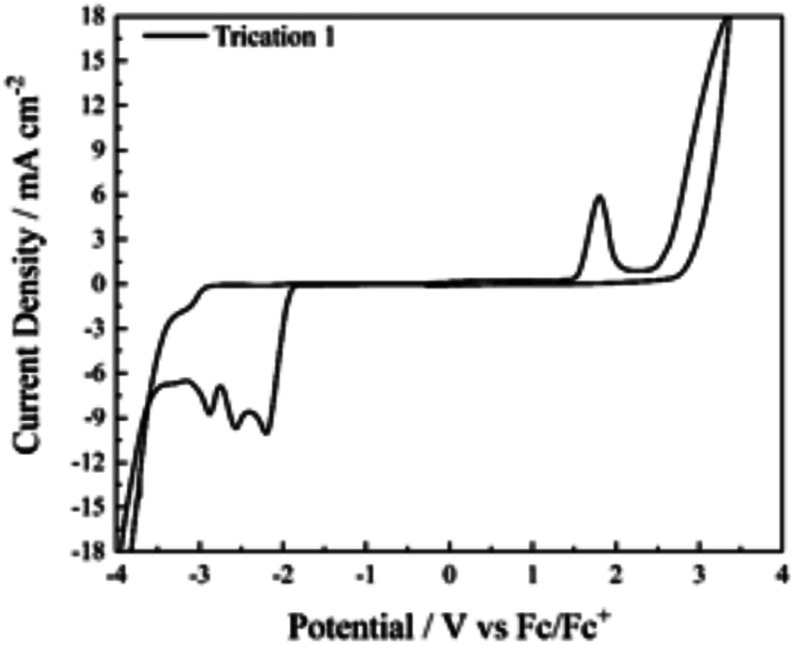
Cyclic voltammogram of the Tf_2_N^−^ salt of 1. Scans were conducted at 50 mV s^−1^ from *V*_ocp_, first negative, then positive. The three sequential one-electron reduction steps are clearly apparent.

**Fig. 5:**
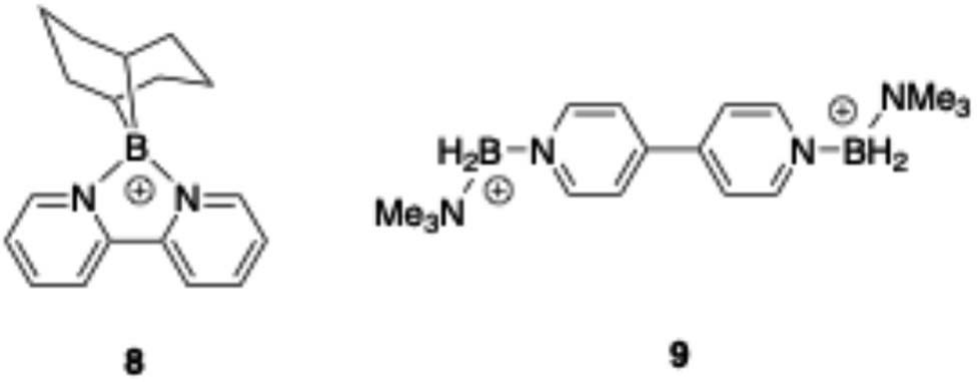
Structures of other boronium cations for which reduction potentials have been measured.

Sweeping negative from the open circuit potential (OCP) in the presence of cation 1 reveals three clearly separated, irreversible reductions at −2.19 V, −2.55 V, and −2.87 V (*vs.* Fc/Fc^+^) (Fig. 4). In context, we were surprised by these values since two other boronium cations of lesser positive charge (8 (ref. 28) and 9,^29^Fig. 5) which are also supported by pyridine-derivative ligands, are reduced at lower potentials (−1.62 and −0.673/−1.003 V, respectively). Intuitively, we expected the more positively charged material to reduce more readily.

Further by way of context, the structurally similar tetraphenylphosphonium (PPh_4_^+^) monocation exhibits a single reduction potential of −3.25 V (*vs.* Fc/Fc^+^). Clearly multiple factors, including the formal charge on the cation and the electronic nature of the ligands can contribute to the vastly different reduction potentials between these cations despite similarities in structure.^30^

On the return sweep in the CV of 1(Tf_2_N)_3_, a single oxidation was seen at 1.8 V (*vs.* Fc/Fc+) which is consistent with studies on neat boronium electrolytes with Tf_2_N^−^ anions. The presence of this peak, which is dependent on initial sweeps beyond the cathodic limit of the cell, is believed to be the oxidation of the surface–electrolyte interface (SEI) layer which assembles at the onset of cation degradation.^26,27,31,32^ Continuous sweeping of the electrode reveals reduced cathodic activity, likely due to surface passivation from SEI accumulation, while the intensity and position of the oxidative peak remains consistent. In addition, the Tf_2_N^−^ salts of 2 and 3 were also tested for electrochemical activity in acetonitrile (Fig. S3†). Unlike the Tf_2_N^−^ salt of cation 1, both compounds exhibited broad, single step reductions when sweeping from OCP to negative potentials.^33^ The oxidation peak observed on return sweeps was also present, though significantly altered in shape from what was observed for cation 1. The difference in behavior between these samples suggest potentially different decomposition pathways as the test cell approaches either the cathodic or anodic limiting potential, warranting further studies on the reductive products of any future boronium (+3) salts as well.^31^

## Conclusions

Developing stable boronium (+3) ions, especially with respect to water insensitivity, was a key objective of the present work and one which was clearly achieved. Doing so was made possible by using pyridine ligands with strongly electron-donating dialkylamine groups in the ring 4-position. By using strongly electron-donating 4-aminopyridines as ligands, bromide salts of boronium (+3) cations were formed that are stable in water on a pH-independent basis, and for long time periods, a heretofore unprecedented observation. The Tf_2_N^−^ salts of these cations also have high degrees of thermal stability and undergo clean 3-electron electrochemical reductions at potentials more negative than those typical of boronium (+1) cations, an unexpected outcome. This combination of attributes suggests that further studies on these salts as electrolytes or additives to existing electrolytic cell designs is warranted, as is exploring their potential for crystal engineering and the formation of hybrid organic–inorganic solids.

## Author contributions

The project was conceptualized by JHD. Synthetic work was done by MEC, GAM, RAO, and JHD. NMR studies were carried out by MEC and GAM. Electrochemical and thermal studies were conducted by, and the results analysed by, CDS, PCT, and DPD. X-ray crystal structures were acquired by MZ. Computations were carried out by EAS and AW. Funding was acquired by JHD, PCT, and DPD. All authors reviewed and corrected the manuscript as needed.

## Conflicts of interest

There are no conflicts to declare.

## Supplementary Material

RA-015-D5RA02301G-s001

RA-015-D5RA02301G-s002

## Data Availability

The data supporting this article have been included as part of the ESI.†

## References

[cit1] Piers W. E., Bourke S. C., Conroy K. D. (2005). Angew Chem. Int. Ed. Engl..

[cit2] Chitnis S. S., Robertson A. P. M., Burford N., Patrick B. O., McDonald R., Ferguson M. J. (2015). Chem. Sci..

[cit3] Corbo R., Ryan G. F., Haghighatbin M. A., Hogan C. F., Wilson D. J. D., Hulett M. D., Barnard P. J., Dutton J. L. (2016). Inorg. Chem..

[cit4] Ling I., Skelton B. W., Sobolev A. N., Alias Y., Khor Z. C., Raston C. L. (2020). New J. Chem..

[cit5] Teodoro J. S., Palmeira C. M., Rolo A. P. (2018). Methods Mol. Biol..

[cit6] Díaz Morales U. M., Corma-Canós A. (2018). Chem.–Eur. J..

[cit7] Kore B., Jamshidi M., Gardner J. M. (2024). Mater. Adv..

[cit8] Bohl R. D., Galloway G. L. (1969). J. Sci. Lab., Denison Univ..

[cit9] Makosky C. W., Galloway G. L., Ryschkewitsch G. E. (1967). Inorg. Chem..

[cit10] Tandom R., Unzner T., Nigst T. A., De Rycke N., Mayer P., Wendt B., David O. R. P., Zipse K. (2013). Chem.–Eur. J..

[cit11] Wilde J. H., Smith J. A., Dickie D. A., Harman W. D. (2019). J. Am. Chem. Soc..

[cit12] Forsythe P., Frampton R., Johnson C. D., Katritzky A. R. (1972). J. Chem. Soc., Perkin Trans. 2.

[cit13] Frisch M. J. , et al., Gaussian 16, Revision C.01, Wallingford, CT, 2016

[cit14] Wavefunction Inc., Irvine, CA, 2024

[cit15] Vargas-Baca I., Findlater M., Powell A., Vasudevan K. V., Cowley A. H. (2008). Dalton Trans..

[cit16] Fox P. A., Griffin S. T., Reichert W. M., Salter E. A., Smith A. B., Tickell M. D., Wicker B. F., Cioffi E. A., Davis Jr J. H., Rogers R. D., Wierzbicki A. (2005). Chem. Commun..

[cit17] Ruther T., Huynh T. D., Huang J., Hollenkamp A. F., Salter E. A., Wierzbicki A., Mattson K., Lewis A., Davis Jr J. H. (2009). Chem. Mater..

[cit18] Stachurski C. D., Davis Jr J. H., Cosby T., Crowley M. E., Larm N. E., Ballentine M. G., O'Brien R. A., Zeller M., Salter E. A., Wierzbicki A., Trulove P. C., Durkin D. P. (2023). Inorg. Chem..

[cit19] Fredlake C. P., Crosthwaite J. M., Hert D. G., Aki S. N. V. K., Brennecke J. F. (2004). J. Chem. Eng. Data.

[cit20] Del Sesto R. E., Corley C., Robertson A., Wilkes J. S. (2005). J. Organomet. Chem..

[cit21] Malarz K. M., Mrozek-Wilczkiewicz A., Musiol R., Zorębski E., Dzida M. (2017). ACS Sustainable Chem. Eng..

[cit22] Cao Y., Mu T. (2014). Ind. Eng. Chem. Res..

[cit23] Soltani M., McGeehee J. L., Stenson A. C., O'Brien R. A., Duranty E. R., Salter E. A., Wierzbicki A., Glover T. G., Davis Jr J. H. (2020). RSC Adv..

[cit24] Rabideau B. D., West K. N., Davis Jr J. H. (2018). Chem. Commun..

[cit25] Harris M. A., Kinsey T., Wagle D. V., Sangoro J. (2021). Proc. Natl. Acad. Sci. U. S. A..

[cit26] Clarke-Hannaford J., Breedon M., Rüther T., Spencer M. J. S. (2020). ACS Appl. Energy Mater..

[cit27] Clarke-Hannaford J., Breedon M., Rüther T., Johansson P., Spencer M. J. S. (2021). Chemistry.

[cit28] Hunig S., Wehner I. (1989). Heterocycles.

[cit29] Dorman S. C., O’Brien R. A., Lewis A. T., Salter E. A., Wierzbicki A., Hixon P. W., Sykora R. E., Mirjafari A., Davis Jr J. H. (2011). Chemical Commun..

[cit30] Lang C. M., Kim K., Guerra L., Kohl P. A. (2005). J. Phys. Chem. B.

[cit31] Clarke-Hannaford J., Breedon M., Rüther T., Spencer M. J. S. (2020). ACS Appl. Energy Mater..

[cit32] Clarke-Hannaford J., Breedon M., Rüther T., Johansson P., Spencer M. J. S. (2021). Batteries Supercaps.

[cit33] Stachurski C. D., Cho W., Kinnaman C. M., Zeller M., Davis Jr J. H., Larm N. E., Trulove P. C., Durkin D. P. (2024). J. Electrochem. Soc..

