# Supplementary material for: Salts of boronium (+3) ions with unprecedented hydrolytic stability. Design, synthesis, single-crystal X-ray structures, electrochemistry, and thermophysical properties
Source: RSC Adv. 2025 Jun 12;15(25):19947–53. doi: 10.1039/d5ra02301g (PMC12159592; doi:10.1039/d5ra02301g)
Supplement: RA-015-D5RA02301G-s001 [file RA-015-D5RA02301G-s001.pdf]

Supplementary Information (SI) for RSC Advances.
This journal is © The Royal Society of Chemistry 2025

Salts of boronium (+3) ions with unprecedented hydrolytic stability. Design, synthesis, single-crystal X-ray structures, electrochemistry, and thermophysical properties

Margaret E. Crowley,[a] Christopher D. Stachurski,*[b] James H. Davis, Jr.,*[a] Matthias Zeller,[c] Gabriel A. Merchant,[a] E. A. Salter,[a] A. Wierzbicki,[a] Richard A. O'Brien,[a] Paul C. Trulove,[b] and David P. Durkin,[b]

a Department of Chemistry, University of South Alabama b
Department of Chemistry, United States Naval Academy c
Department of Chemistry, Purdue University

Supporting information

Table of Contents

Page	Content
3	Figure S1
4	Figure S2
5	Figure S3
5	Synthetic and safety considerations
6	Compiled NMR data
8	Original NMR spectra
19	Crystal structures
21	Computational details and data
37	pH vs. T stability NMRs, compound 1Br3
50	pH vs. T stability NMRs, compound 2Br3
61	CCD deposition numbers
63	Check cif files

Figure S1. Thermal gravimetric analysis traces for the Tf_2N^- salts of boroniums **1-5**.

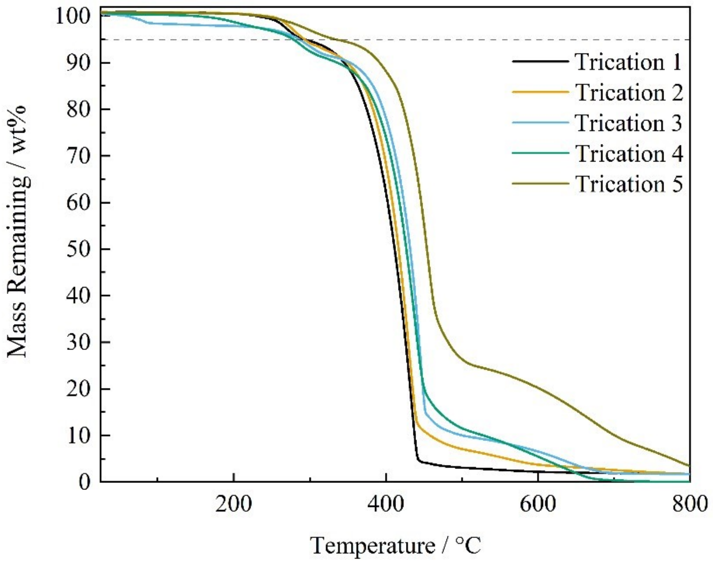

Thermal decomposition curves of the Tf_2N^- salts of boroniums **1-5**.

Figure S2. Differential scanning calorimetry traces from the Tf_2N^- salts of boroniums **1-5**.

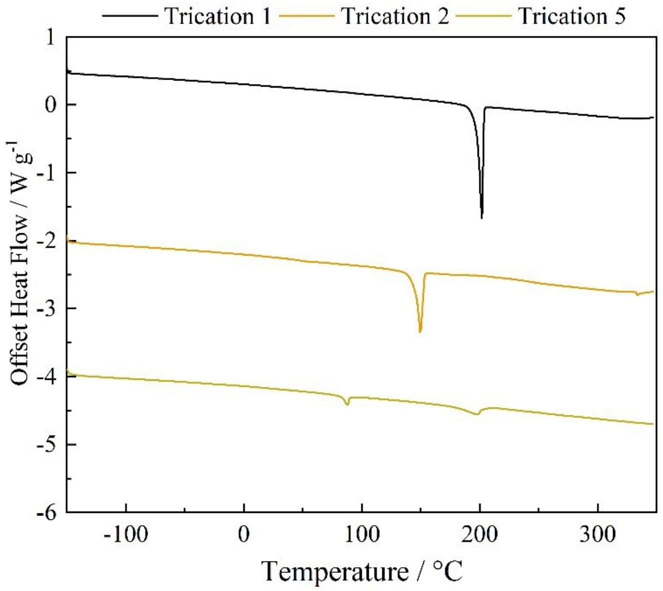

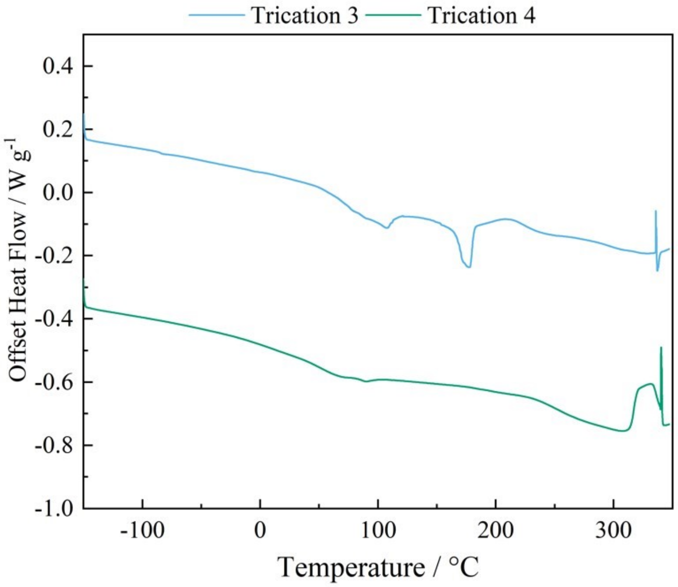

Exo Up

Differential scanning calorimetry traces of the Tf_2N^- salts of **1**, **2**, and **5** (upper) and **3** and **4** (lower). Samples were preconditioned at 50 °C, then cooled to -150 °C before heating to 350 °C at 10 °C min^{-1}.

Figure S3. Cyclic voltammograms of the Tf_2N^- salts of boroniums **2** (left) and 3 (right).

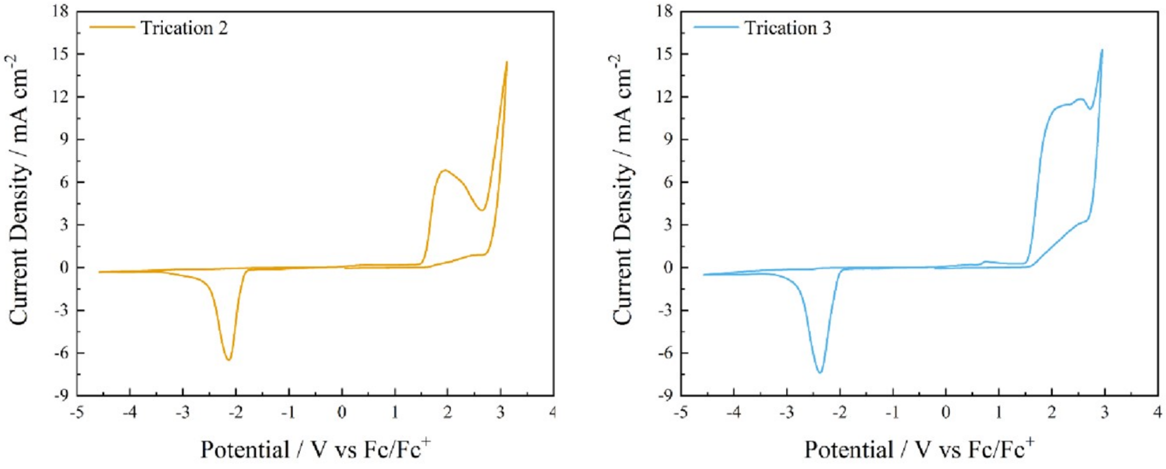

Synthetic comments/safety considerations

Chlorobenzene boils at 132 °C at ambient pressure. Consequently, the addition of the Me_2SBBr_3 through a side neck during reflux must be done carefully; In our experience, the use of a funnel with a long, wide stem allows the addition to be conducted safely. However, portions of added solid should be modest, because Me_2S is instantly released by the solid upon contact with the hot solvent and the former will vent through the open neck. Clearly this must be done in a well-functioning hood! As an important aside – in our hands, doing the solid addition at temperatures below the boiling point of the chlorobenzene resulted in the formation of sticky, intractable masses which froze the magnetic stirbar in place, and which were very difficult to work up.

Compiled NMR data: ^{1}H, ^{13}C, ^{10}B*, ^{19}F

All spectra were acquired on a JEOL JNM-ECA 500 in 5 mm tubes. All deuterated solvents purchased from MilliporeSigma, Cambridge Isotope Laboratories, or Oakwood Chemical, and used as received.

*In our experience, ^{10}B NMR spectra of boronium ions have narrower line widths and smoother baselines than do those produced by (more commonly used) ^{11}B NMR. No baseline correction was used in these spectra.

All NMR data are for the depicted cation paired with the Tf_2N anion. Coupling constant for multiplets in the 1H spectra are shown on the included spectra.

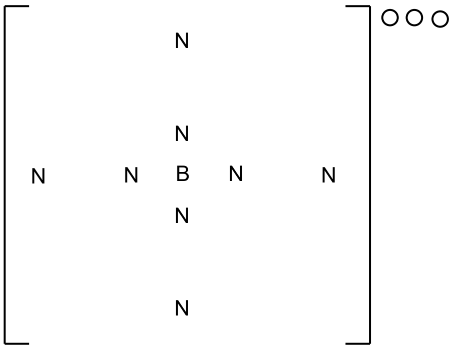

Compound 1. ^{1}H (470 MHz, acetone-d_6) ppm: 3.31, s, 24H, NMe_2; 7.10, d (J = 7.11 Hz), 8H, CH; 8.01, d (J = 8.03 Hz), 8H. ^{13}C (126 MHz, acetone-d_6) ppm: 40.34, NMe_2; 109.48; 121.2 (quartet), CF_3; 143.74, 157.93. ^{10}B (54MHz, acetone-d_6) ppm: s, 1.81; ^{19}F (470 MHz, acetone-d_6) ppm: s, -79.70.

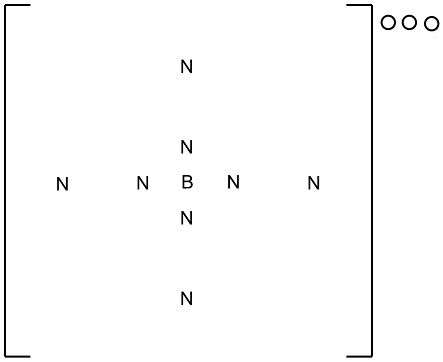

Compound 2. ^{1}H (500 MHz, chloroform-d) ppm: 1.72, overlapping, unresolved multiplets, 6H, 3 x CH_2; 3.61, unresolved multiplet, 4H, 2 x CH_2; 6.92, d (J = 7.23 Hz), 2H, CH; 7.56, d (J = 7.99 Hz), 2H, CH. ^{13}C (126 MHz, chloroform-d) ppm: 23.55, CH_2; 25.29, CH_2; 108.94, CH; 119.5 (quartet), CF_3; 142.69, CH; 155.45, CH. ^{10}B (54MHz, chloroform-d) ppm: s, 1.46; ^{19}F (470 MHz, chloroform-d) ppm: s, -79.65.

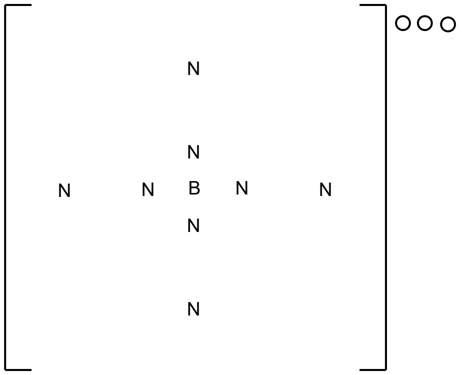

Compound 3. ^{1}H (470 MHz, acetone-d_6) ppm: 2.12, unresolved, overlapping m, 4H, CH_2; 3.54 (J = 3.63 Hz), 3.64 (J = 3.52 Hz), m, 2H & 2H, CH_2; 6.90, d (J = 6.96 Hz), 4H, CH; 8.11, d (J = 8.22 Hz), 4H, CH. ^{13}C (126 MHz, acetone-d_6) 29.49, 29.54 CH_2; 48.57,

49.53, CH_2; 108.38, C; 110.21 CH; 121.0 (quartet), CF_3; 142.48, 143.57 CH; 155.09, CH. ^{10}B (54MHz, acetone-d_6) ppm: s, 1.85; ^{19}F (470 MHz, acetone-d_6) ppm: s, -79.66.

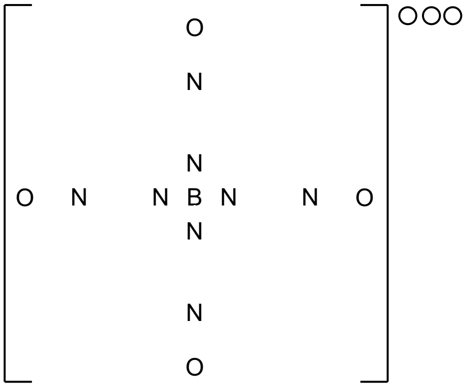

Compound 4. ^1H (470 MHz, acetone-d_6) ppm: 3.81, s [overlapping unresolved multiplets], 8H, CH_2; 7.32, d (J = 7.29 Hz), 4H, CH; 8.15, d (J = 8.12 Hz), 4H, CH. ^{13}C (126 MHz, acetone-d_6) ppm: 47.45, CH_2; 66.57, CH_2; 109.48, CH; 144.52, C; 157.67, CH; 120.8 (quartet), CF_3. ^{10}B (54MHz, acetone-d_6) ppm: s, 1.64; ^{19}F (470 MHz, acetone-d_6) ppm: s, -79.66.

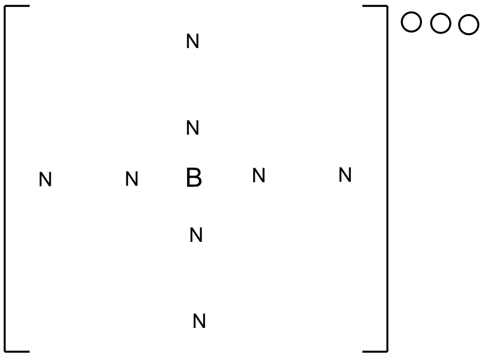

Compound 5. ^1H (470 MHz, acetone-d_6) ppm: 1.96, m, 16H, CH_2; 2.69, m, 16H, CH_2; 3.55, m, 16H, CH_2; 7.57, s, 8H, CH. ^{13}C (126 MHz, acetone-d_6) ppm: 19.86, CH_2; 24.47, CH_2; 25.50, CH_2; 50.45, CH_2; 118.55, C; 121.7 (quartet), CF_3; 135.48, CH; 139.31, CH; 153.20, CH. ^{10}B (54MHz, acetone-d_6) ppm: s, 1.45; ^{19}F (470 MHz, acetone-d_6) ppm: s, -79.67.

Original NMR Spectra (Tf_2N^- salts)

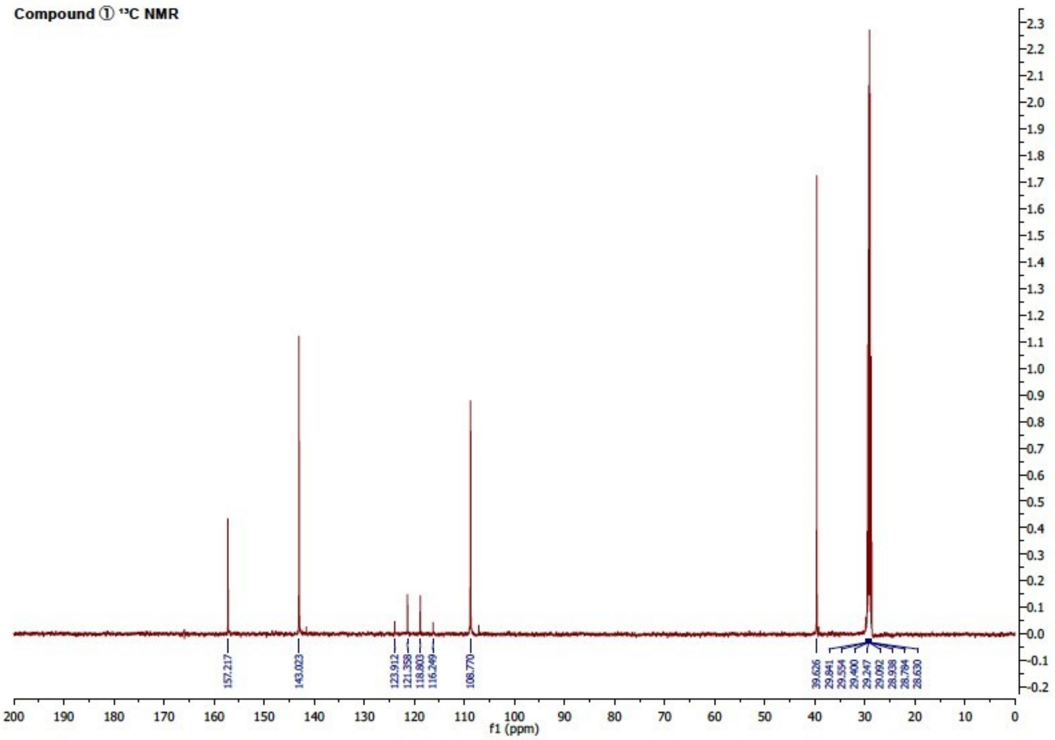

Compound ① ¹H NMR

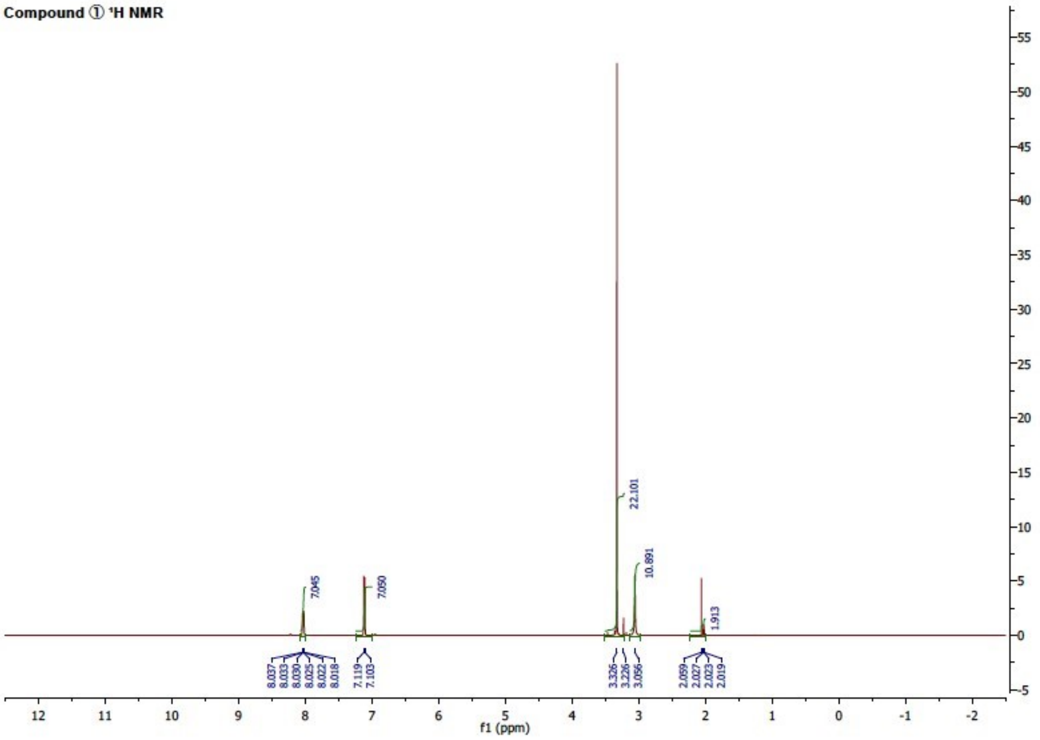

Compound ① ¹¹B NMR

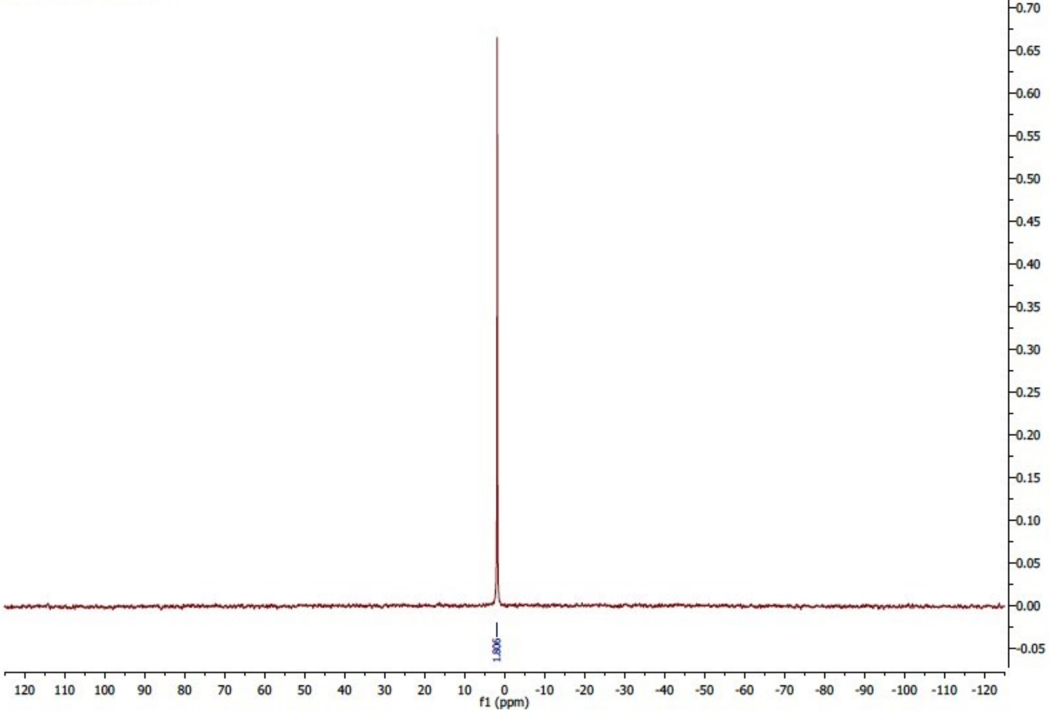

Compound ① ^{19}F NMR

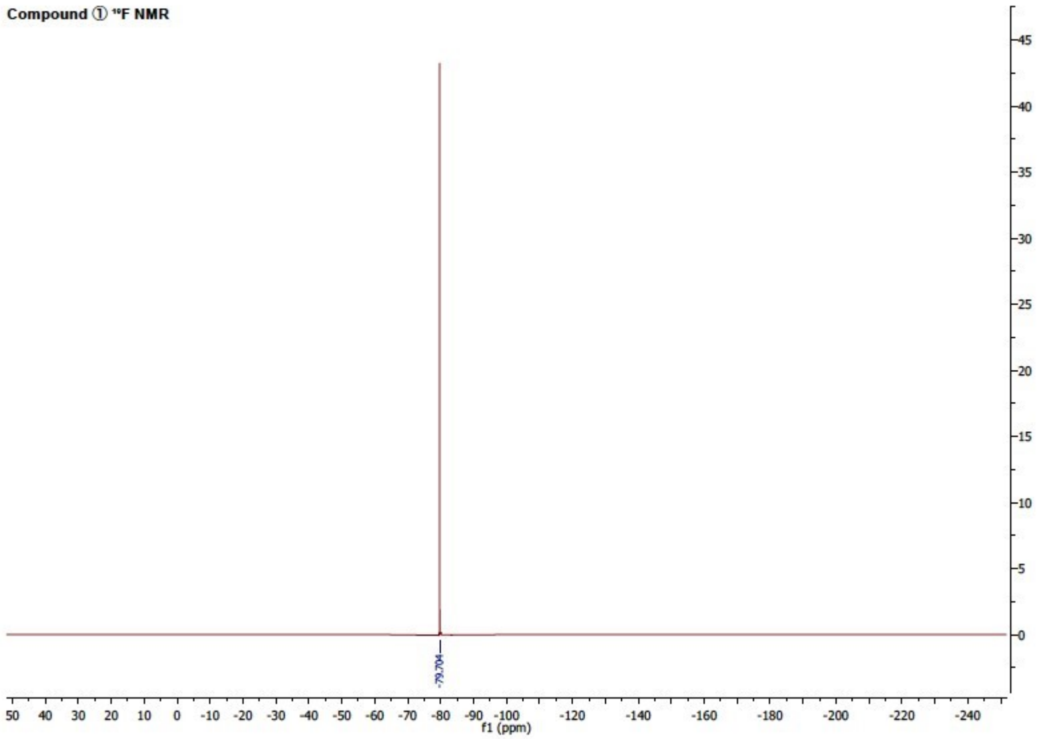

Compound ② ^{13}C NMR

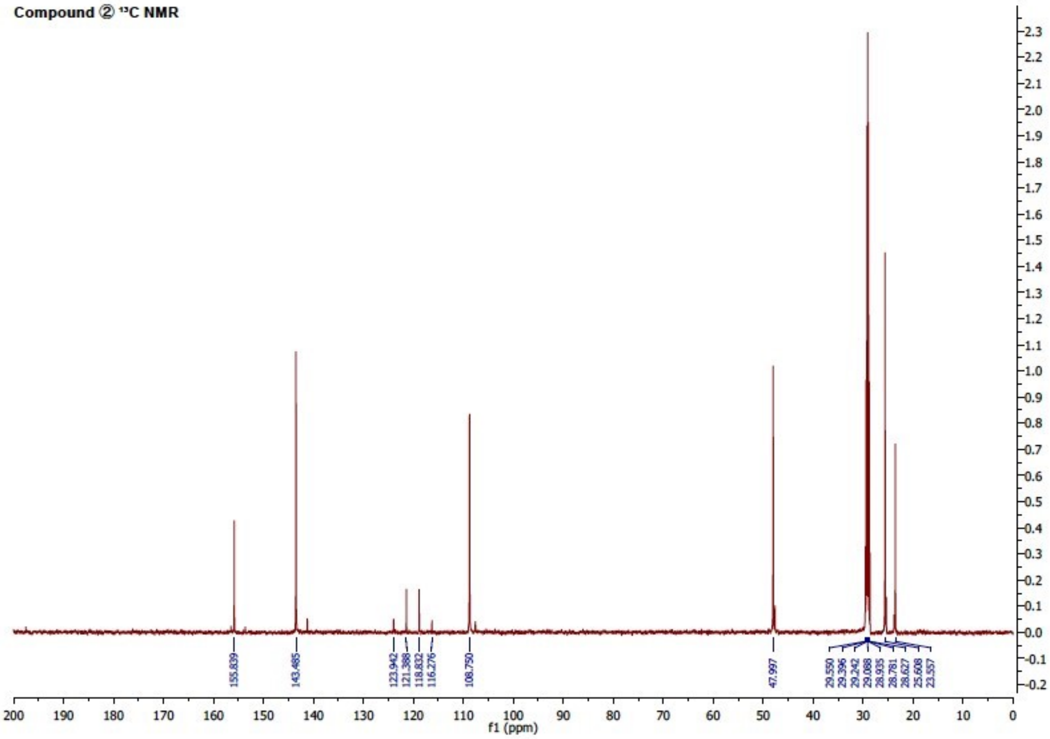

Compound ② ^{1}H NMR

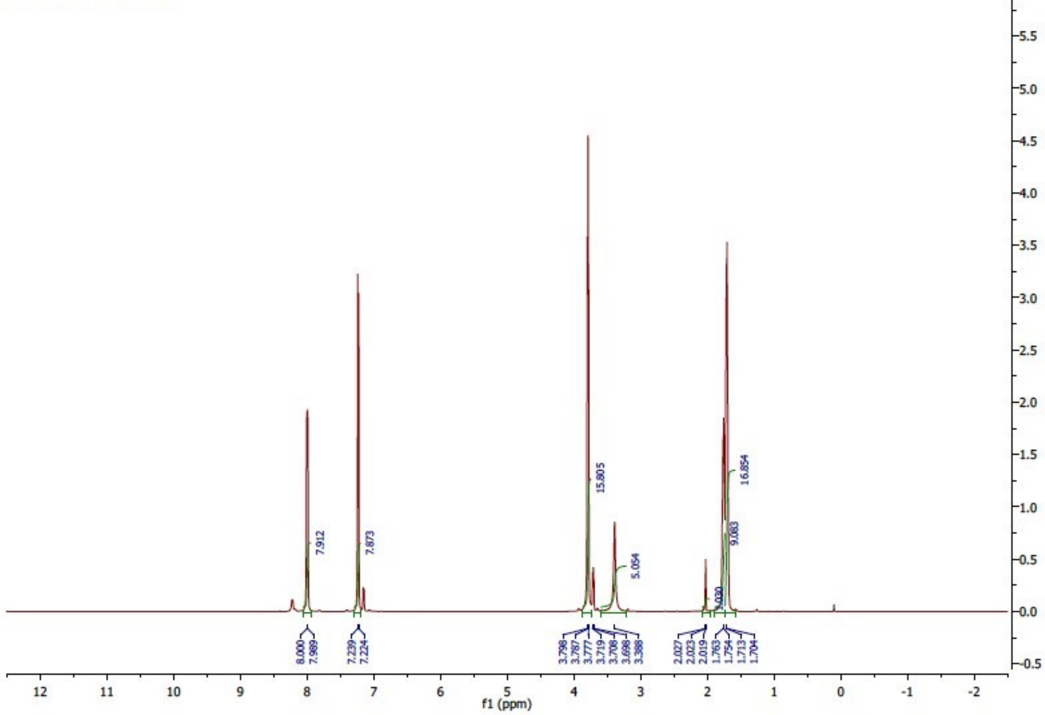

Compound ② ^{10}B NMR

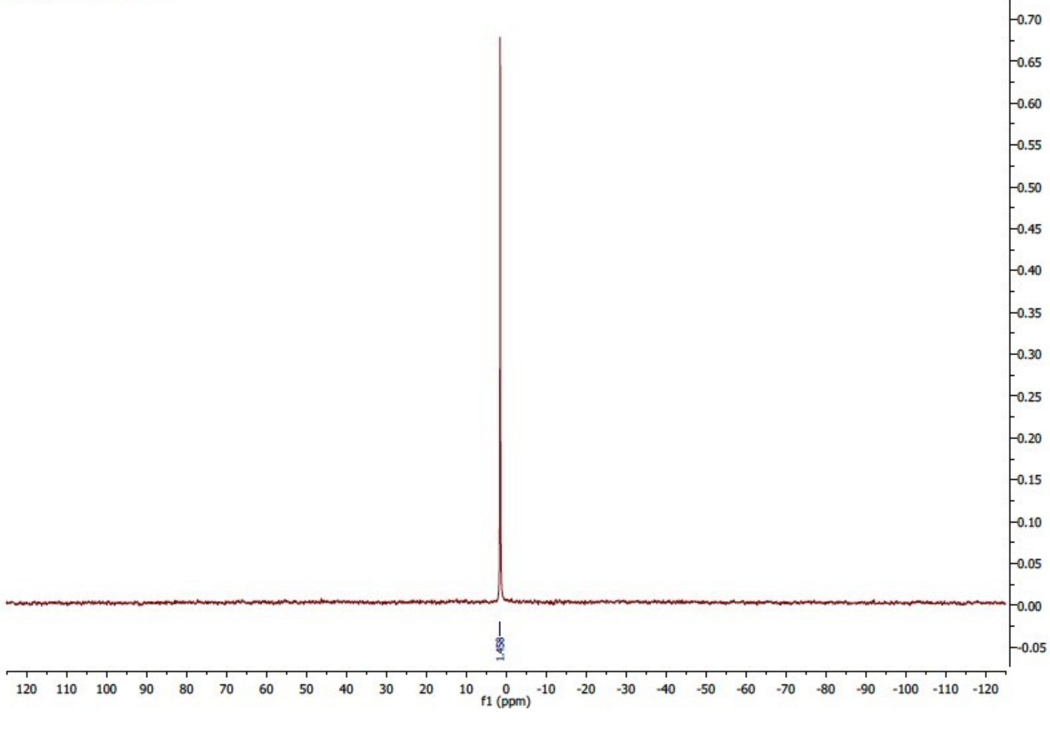

Compound ② ¹⁹F NMR

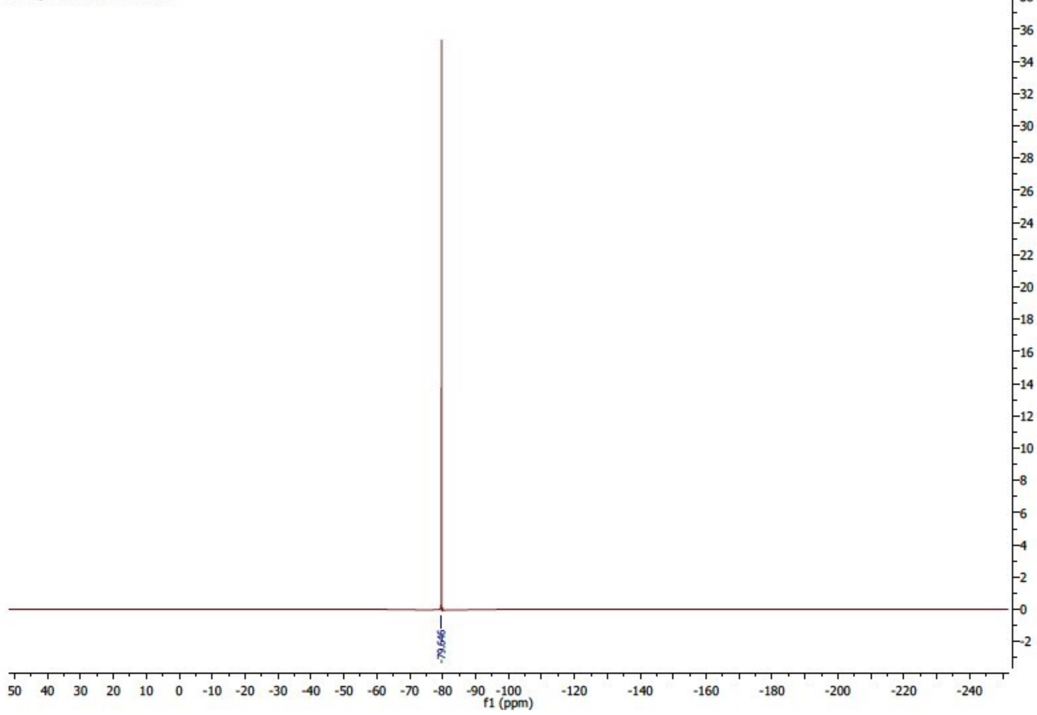

Compound ③ ¹³C NMR

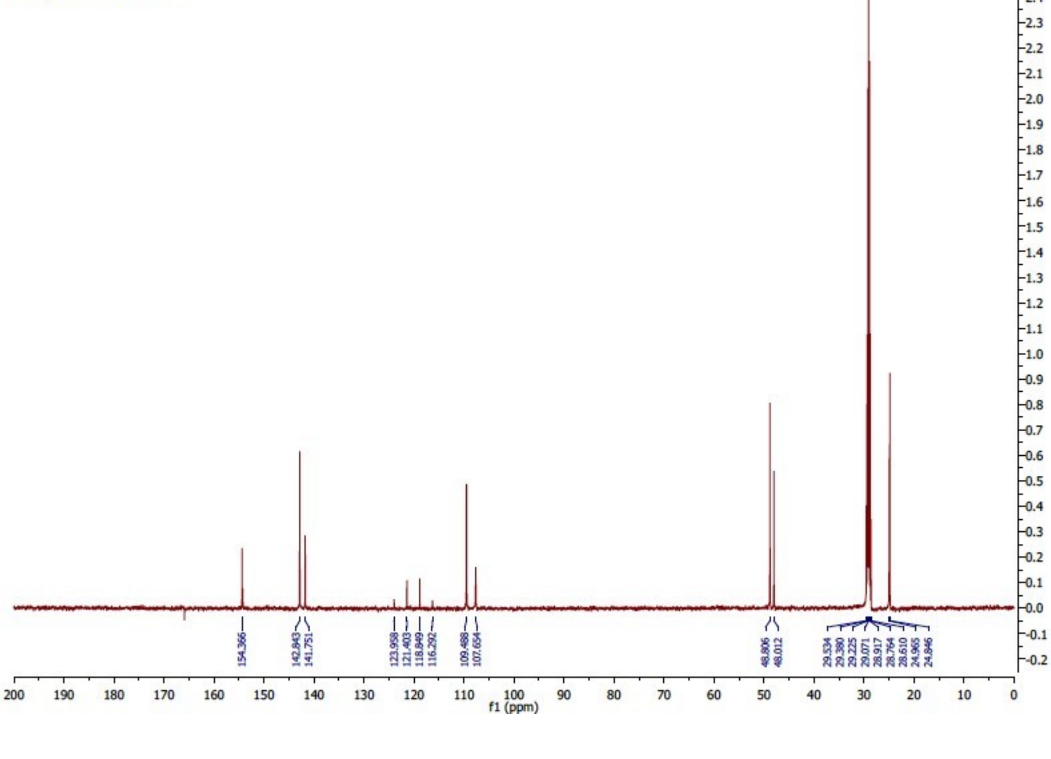

Compound ③ ¹H NMR

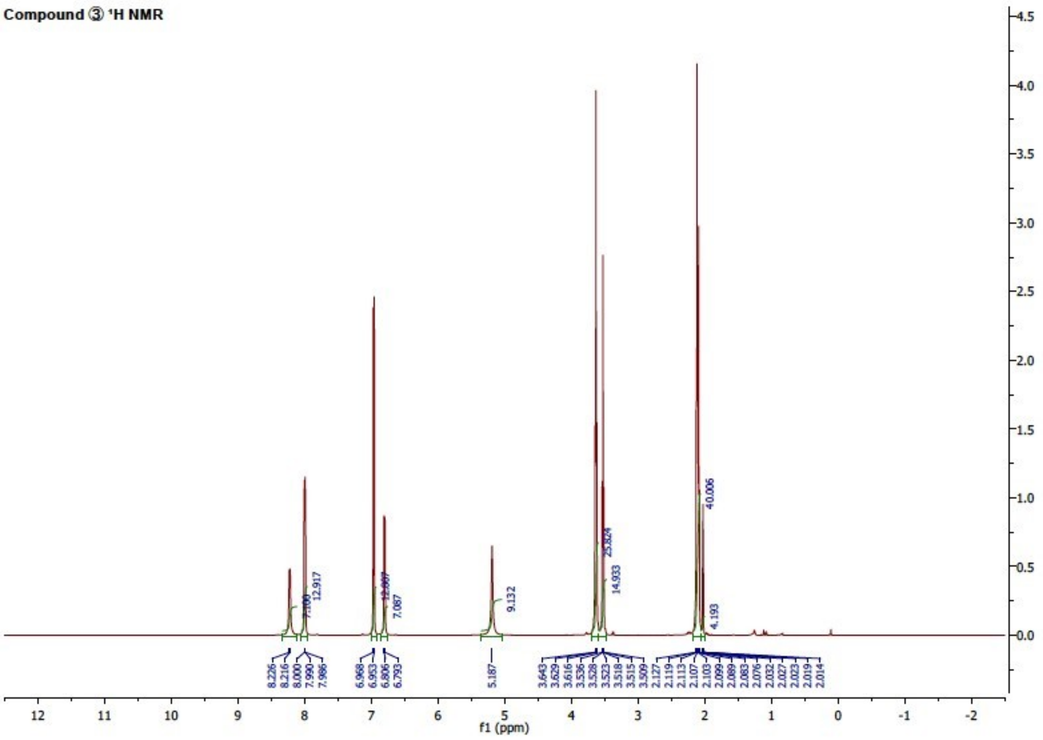

Compound ③ ¹¹B NMR

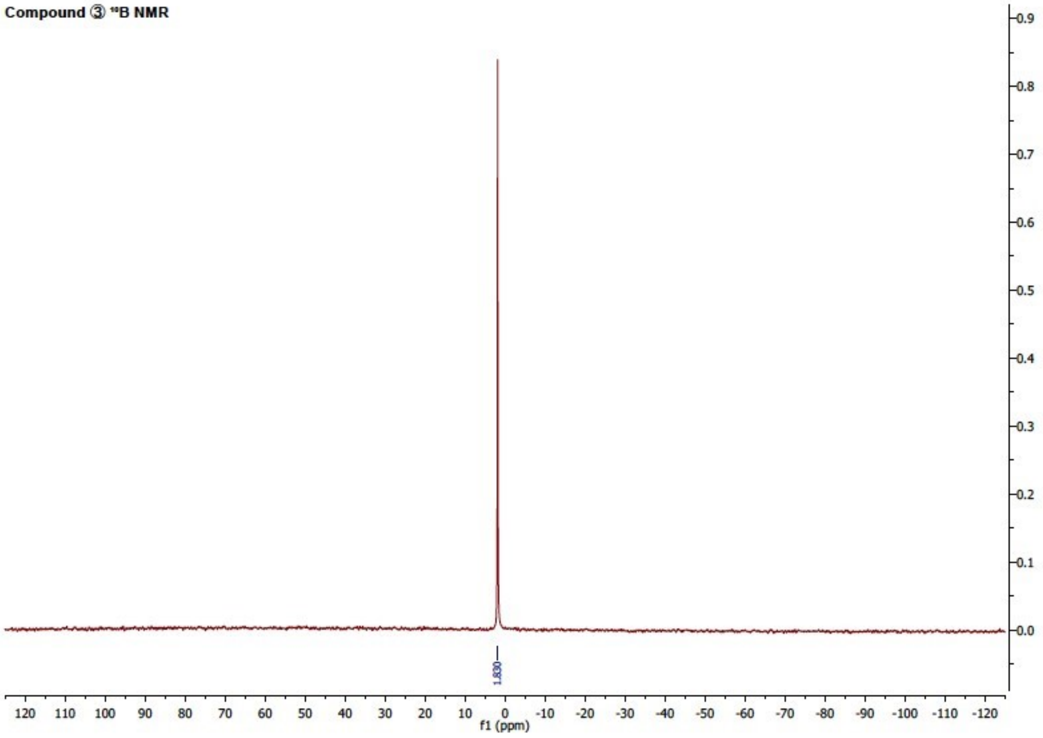

Compound ③ ^{19}F NMR

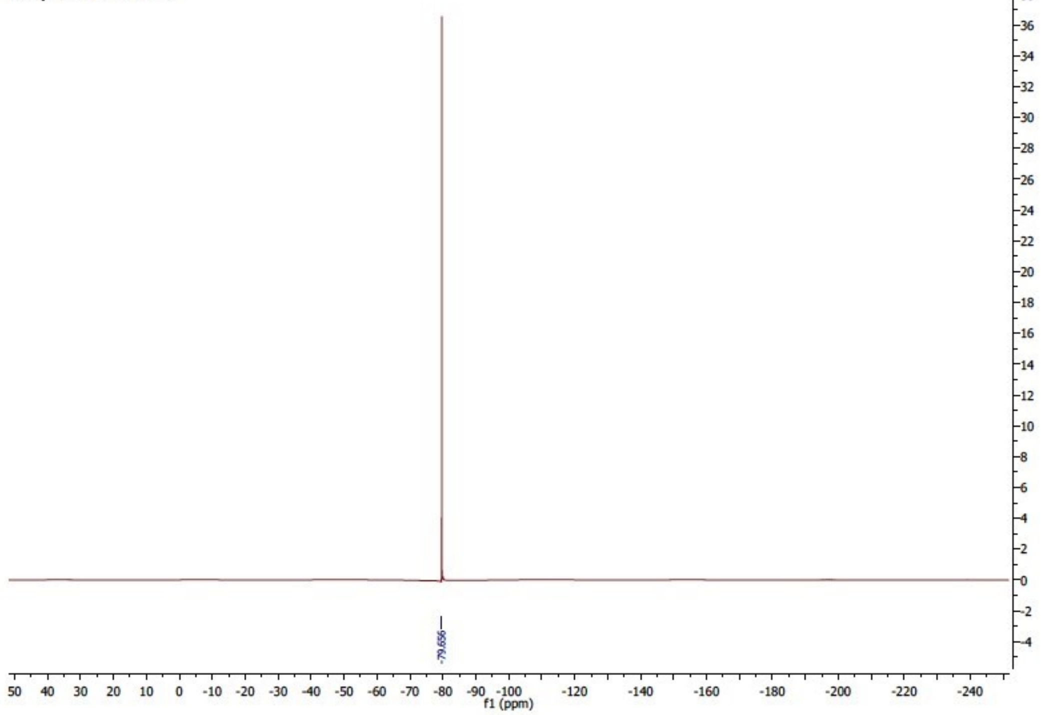

Compound ④ ^{13}C NMR

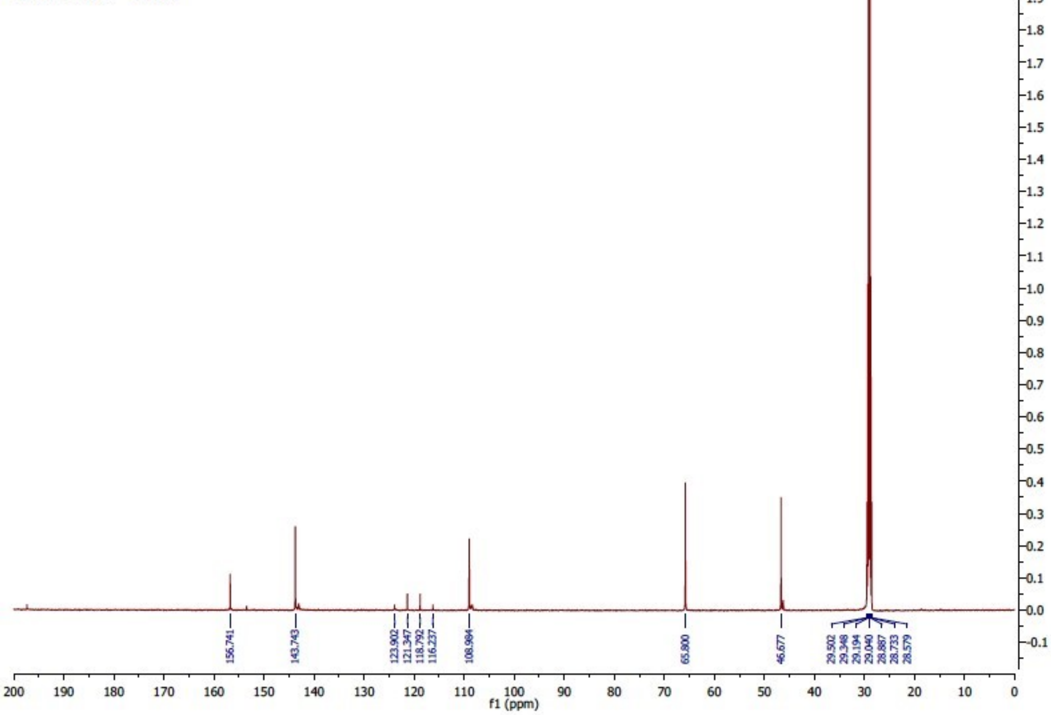

Compound ④ ^{1}H NMR

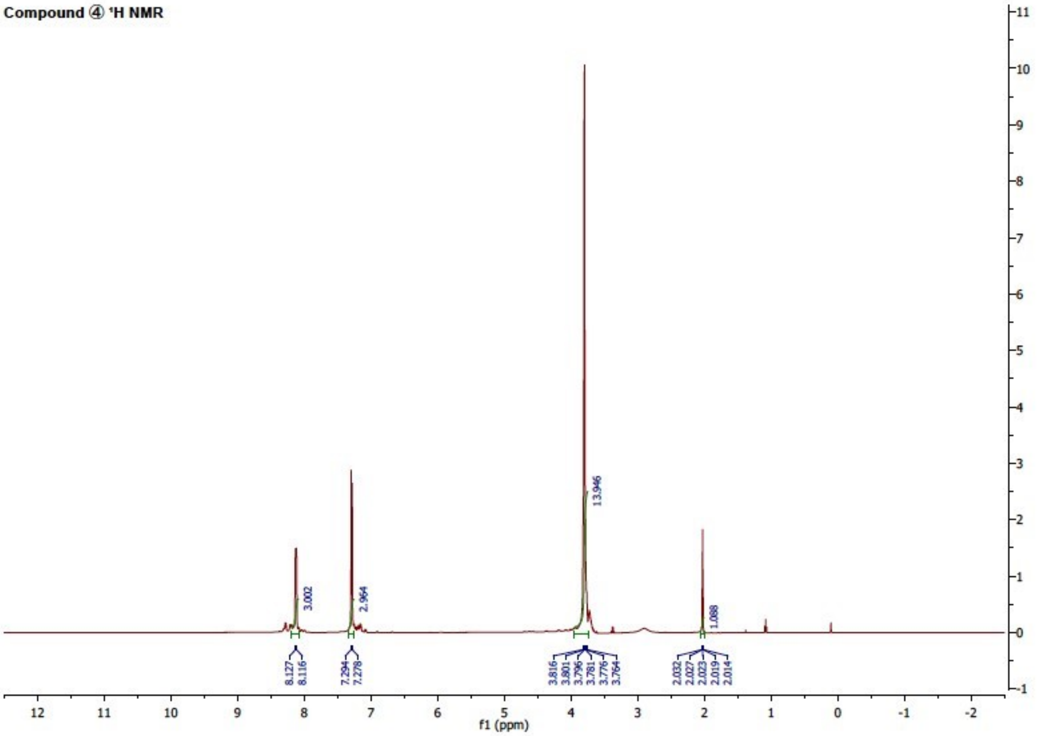

Compound ④ ^{10}B NMR

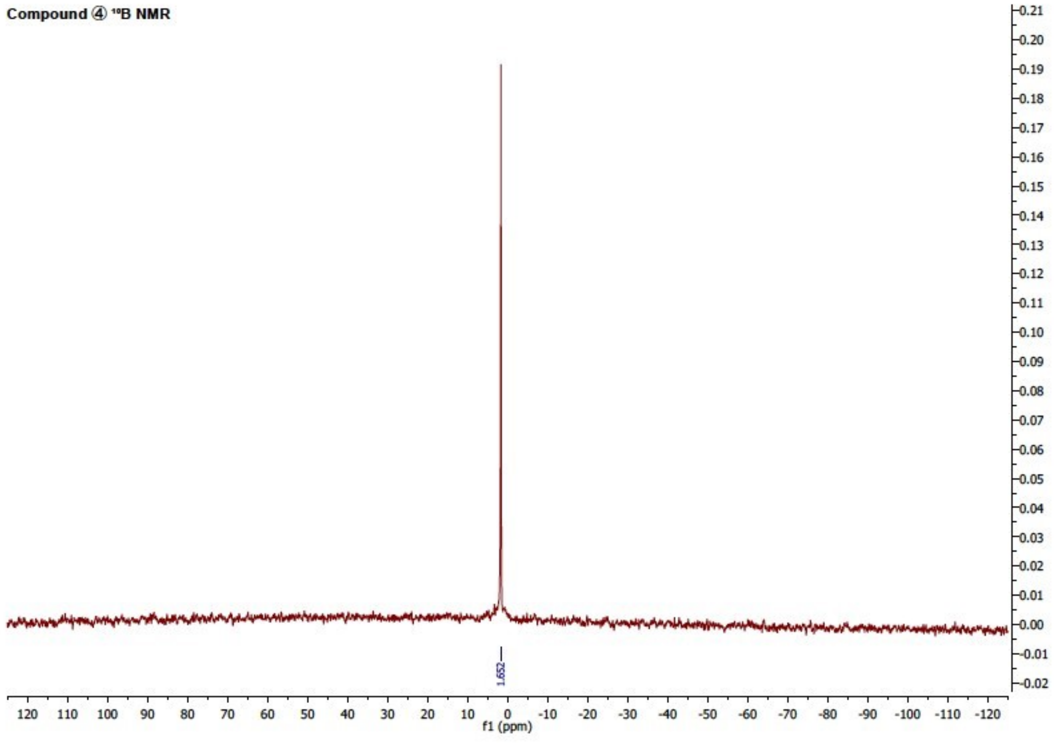

Compound ④ ¹⁹F NMR

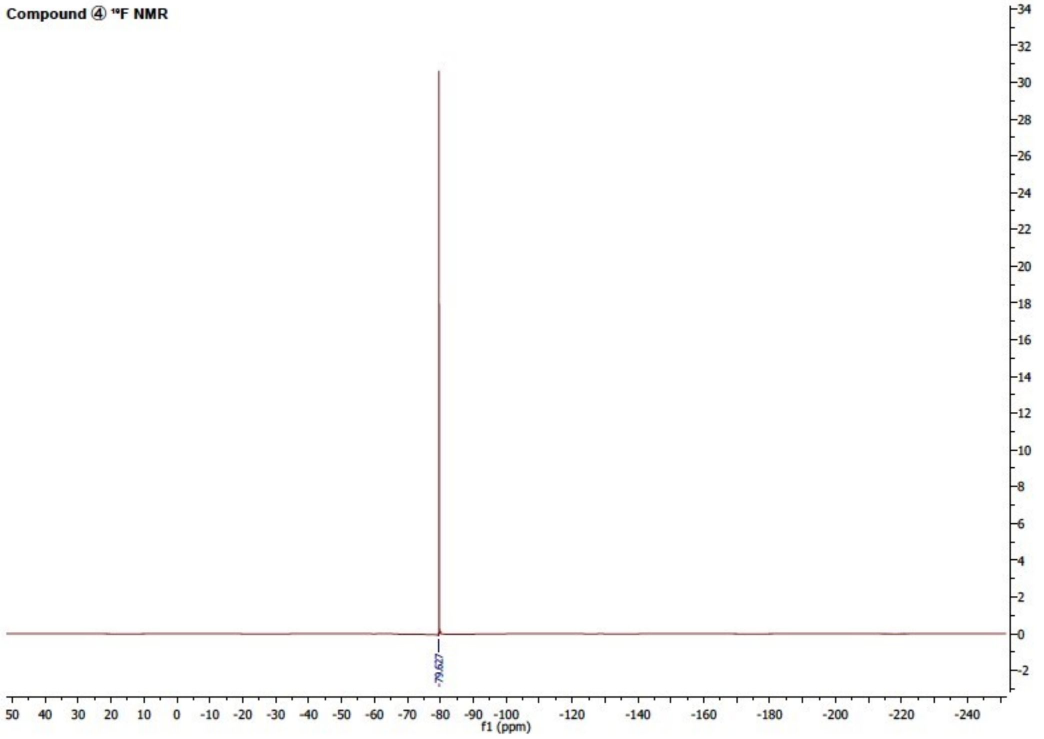

Compound ⑤ ¹³C NMR

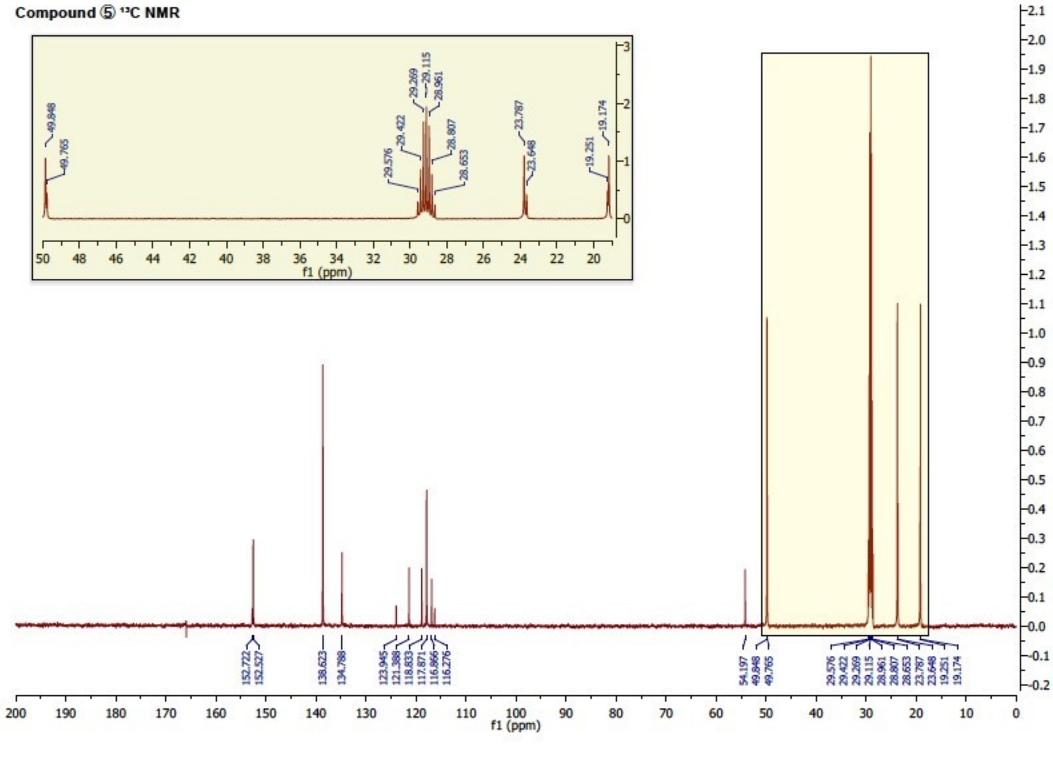

Compound ⑤ ¹H NMR

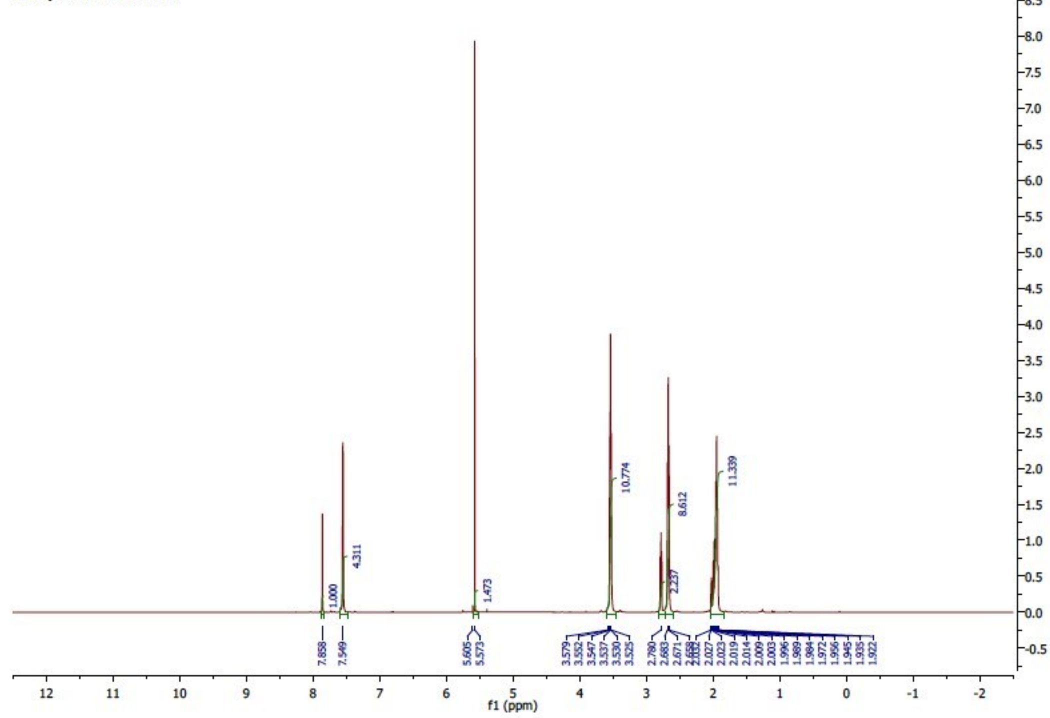

Compound ⑤ ¹¹B NMR

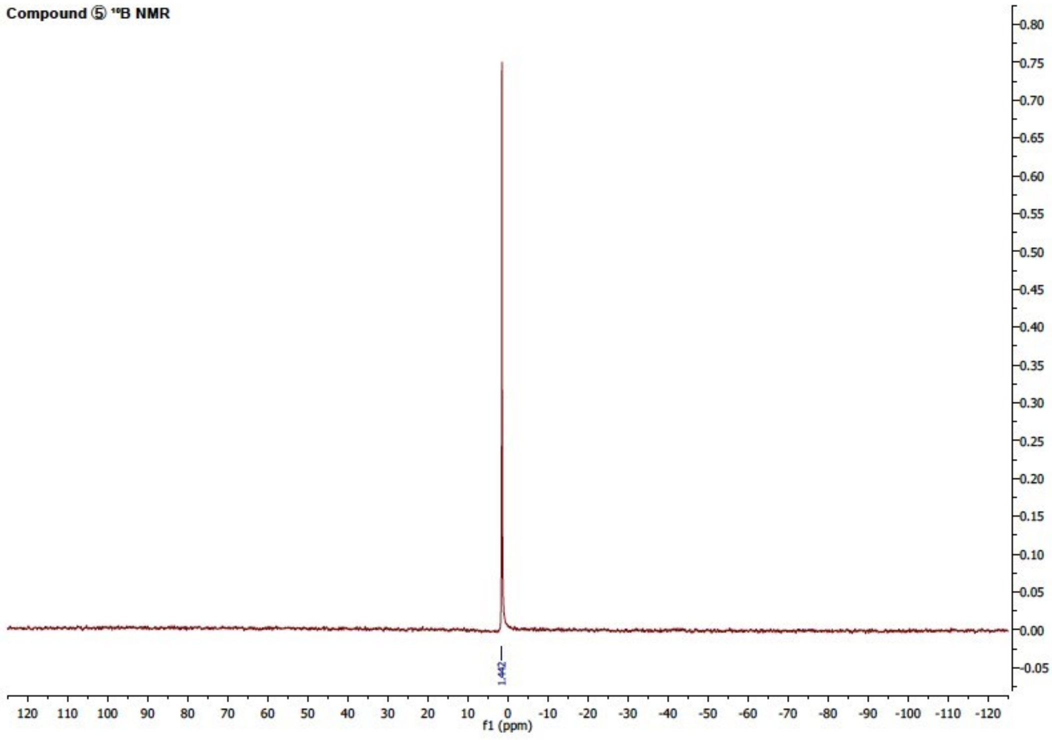

Compound ⑤ ^{19}F NMR

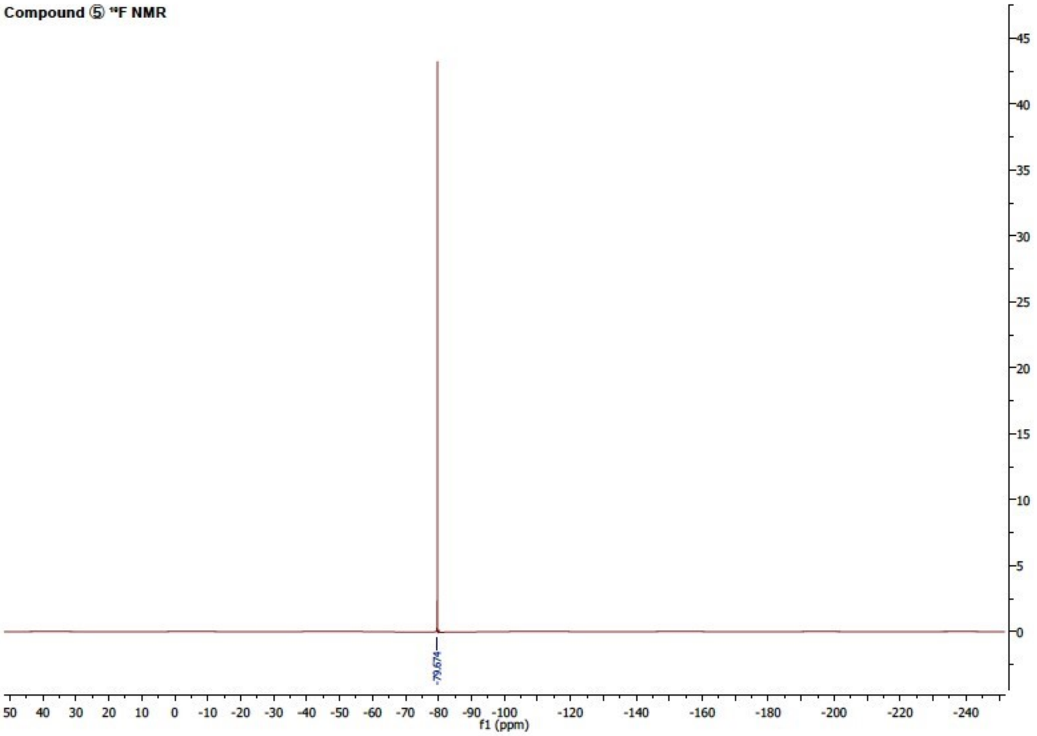

Crystal Structures
(Tf_2N^- anions displayed in muted colours)

Compound 1

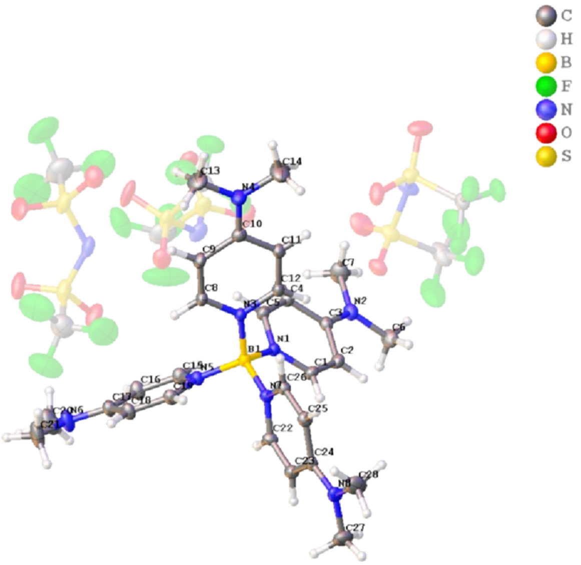

Compound 2

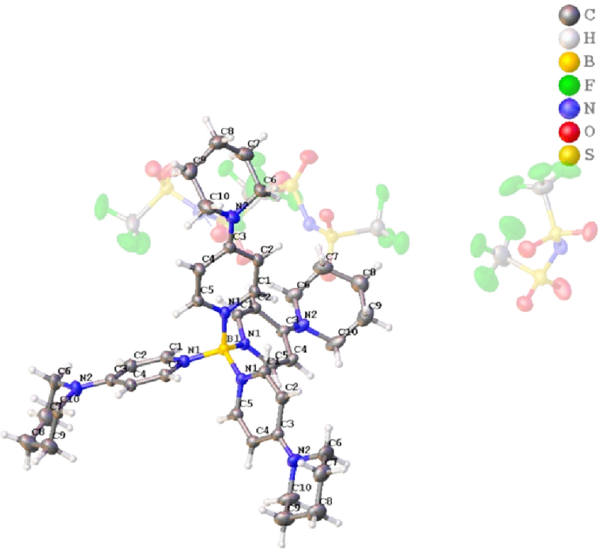

Compound 3

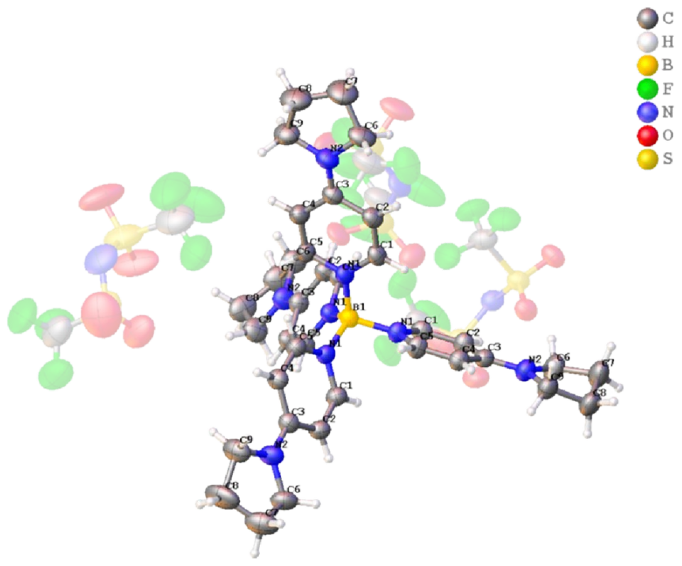

Compound 4 – no crystals suitable for single-crystal structure determination were acquired despite multiple attempts to do so.

Compound 5

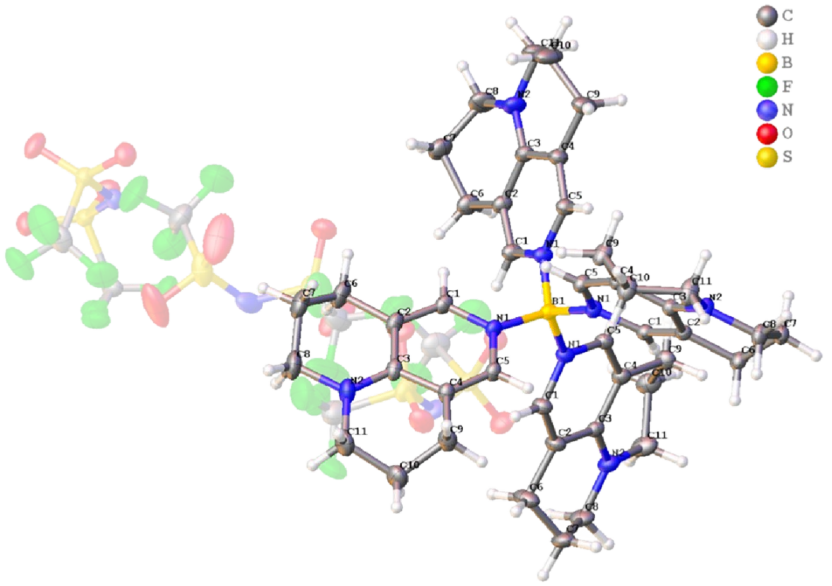

Computational Details

Quantum Calculations

Cations 1-7 (Figure 1 in main manuscript) were optimized in the gas phase using the wB97XD density functional method and the 6-31g(d) basis set as implemented in Gaussian 16 (rev C.01), with S4 symmetry imposed. Vibrational frequency calculations confirmed the structures as stable minima; the structures were then reoptimized using the wB97XD/cc-pvtz computational model, followed again by frequency evaluation which confirmed all real frequencies. Cartesian coordinates for the optimized structures are listed below. After the optimizations, atomic natural bond order (NBO) partial charges were assigned using Gaussian 16 (POP=NPA). Electrostatic potential maps were generated by Spartan'24 using wavefunctions from wB97XD/cc-pvtz single-point calculations.

TRICATION 1 E(RwB97XD) = -1553.25471499 a.u.

Center Number	Atomic Number	Atomic Type	Coordinates (Angstroms) X	Y	Z
1	5	0	0.000000	0.000000	0.000000
2	7	0	1.180575	0.422659	0.938778
3	6	0	0.933056	1.261753	1.987078
4	6	0	2.465967	0.000000	0.794022
5	6	0	1.885637	1.698952	2.843171
6	1	0	-0.092983	1.574972	2.114529
7	6	0	3.479204	0.385477	1.607746
8	1	0	2.662478	-0.690584	-0.009628
9	6	0	3.243478	1.282022	2.691302
10	1	0	1.588552	2.359967	3.640751
11	1	0	4.461823	-0.012210	1.414983
12	7	0	-0.422659	1.180575	-0.938778
13	6	0	-1.261753	0.933056	-1.987078
14	6	0	0.000000	2.465967	-0.794022
15	6	0	-1.698952	1.885637	-2.843171
16	1	0	-1.574972	-0.092983	-2.114529
17	6	0	-0.385477	3.479204	-1.607746
18	1	0	0.690584	2.662478	0.009628
19	6	0	-1.282022	3.243478	-2.691302
20	1	0	-2.359967	1.588552	-3.640751
21	1	0	0.012210	4.461823	-1.414983
22	7	0	-1.180575	-0.422659	0.938778

23	6	0	-2.465967	0.000000	0.794022
24	6	0	-0.933056	-1.261753	1.987078
25	6	0	-3.479204	-0.385477	1.607746
26	1	0	-2.662478	0.690584	-0.009628
27	6	0	-1.885637	-1.698952	2.843171
28	1	0	0.092983	-1.574972	2.114529
29	6	0	-3.243478	-1.282022	2.691302
30	1	0	-4.461823	0.012210	1.414983
31	1	0	-1.588552	-2.359967	3.640751
32	7	0	0.422659	-1.180575	-0.938778
33	6	0	-0.000000	-2.465967	-0.794022
34	6	0	1.261753	-0.933056	-1.987078
35	6	0	0.385477	-3.479204	-1.607746
36	1	0	-0.690584	-2.662478	0.009628
37	6	0	1.698952	-1.885637	-2.843171
38	1	0	1.574972	0.092983	-2.114529
39	6	0	1.282022	-3.243478	-2.691302
40	1	0	-0.012210	-4.461823	-1.414983
41	1	0	2.359967	-1.588552	-3.640751
42	7	0	-4.204427	-1.689209	3.499870
43	7	0	-1.689209	4.204427	-3.499870
44	7	0	4.204427	1.689209	3.499870
45	7	0	1.689209	-4.204427	-3.499870
46	6	0	2.602241	-3.913707	-4.606610
47	1	0	3.543609	-3.508468	-4.236191
48	1	0	2.816154	-4.833126	-5.138448
49	1	0	2.151041	-3.212092	-5.307987
50	6	0	1.230951	-5.582032	-3.311199
51	1	0	1.519434	-5.954184	-2.328467
52	1	0	0.149307	-5.650078	-3.426596
53	1	0	1.692417	-6.213531	-4.061005
54	6	0	-3.913707	-2.602241	4.606610
55	1	0	-3.212092	-2.151041	5.307987
56	1	0	-4.833126	-2.816154	5.138448
57	1	0	-3.508468	-3.543609	4.236191
58	6	0	-5.582032	-1.230951	3.311199
59	1	0	-5.650078	-0.149307	3.426596
60	1	0	-5.954184	-1.519434	2.328467
61	1	0	-6.213531	-1.692417	4.061005
62	6	0	-2.602241	3.913707	-4.606610
63	1	0	-2.151041	3.212092	-5.307987
64	1	0	-3.543609	3.508468	-4.236191
65	1	0	-2.816154	4.833126	-5.138448

66	6	0	-1.230951	5.582032	-3.311199
67	1	0	-1.519434	5.954184	-2.328467
68	1	0	-0.149307	5.650078	-3.426596
69	1	0	-1.692417	6.213531	-4.061005
70	6	0	3.913707	2.602241	4.606610
71	1	0	3.508468	3.543609	4.236191
72	1	0	3.212092	2.151041	5.307987
73	1	0	4.833126	2.816154	5.138448
74	6	0	5.582032	1.230951	3.311199
75	1	0	6.213531	1.692417	4.061005
76	1	0	5.650078	0.149307	3.426596
77	1	0	5.954184	1.519434	2.328467

TRICATION 2 E(RwB97XD) = -2020.25997780 a.u.

Center Number	Atomic Number	Atomic Type	Coordinates (Angstroms) X	Y	Z
1	5	0	0.000000	0.000000	0.000000
2	7	0	1.181852	0.417457	0.935237
3	6	0	0.946891	1.255294	1.986821
4	6	0	2.468348	0.000000	0.786111
5	6	0	1.906745	1.704102	2.826246
6	1	0	-0.079717	1.558690	2.132592
7	6	0	3.487724	0.398296	1.582902
8	1	0	2.661074	-0.701604	-0.009100
9	6	0	3.277775	1.319693	2.658083
10	1	0	1.595795	2.355044	3.624595
11	1	0	4.459228	-0.015294	1.376633
12	7	0	-0.417457	1.181852	-0.935237
13	6	0	-1.255294	0.946891	-1.986821
14	6	0	0.000000	2.468348	-0.786111
15	6	0	-1.704102	1.906745	-2.826246
16	1	0	-1.558690	-0.079717	-2.132592
17	6	0	-0.398296	3.487724	-1.582902
18	1	0	0.701604	2.661074	0.009100
19	6	0	-1.319693	3.277775	-2.658083
20	1	0	-2.355044	1.595795	-3.624595
21	1	0	0.015294	4.459228	-1.376633
22	7	0	-1.181852	-0.417457	0.935237
23	6	0	-2.468348	0.000000	0.786111
24	6	0	-0.946891	-1.255294	1.986821
25	6	0	-3.487724	-0.398296	1.582902

26	1	0	-2.661074	0.701604	-0.009100
27	6	0	-1.906745	-1.704102	2.826246
28	1	0	0.079717	-1.558690	2.132592
29	6	0	-3.277775	-1.319693	2.658083
30	1	0	-4.459228	0.015294	1.376633
31	1	0	-1.595795	-2.355044	3.624595
32	7	0	0.417457	-1.181852	-0.935237
33	6	0	-0.000000	-2.468348	-0.786111
34	6	0	1.255294	-0.946891	-1.986821
35	6	0	0.398296	-3.487724	-1.582902
36	1	0	-0.701604	-2.661074	0.009100
37	6	0	1.704102	-1.906745	-2.826246
38	1	0	1.558690	0.079717	-2.132592
39	6	0	1.319693	-3.277775	-2.658083
40	1	0	-0.015294	-4.459228	-1.376633
41	1	0	2.355044	-1.595795	-3.624595
42	6	0	5.655176	1.339057	3.321448
43	6	0	4.055365	2.644879	4.591688
44	6	0	6.043970	0.529286	4.554260
45	1	0	6.259139	2.245974	3.258147
46	1	0	5.822975	0.782710	2.407967
47	6	0	4.385007	1.885384	5.873121
48	1	0	4.732111	3.492278	4.468644
49	1	0	3.048887	3.043416	4.612922
50	1	0	7.091515	0.244443	4.466811
51	1	0	5.459266	-0.394030	4.573282
52	1	0	4.258856	2.560119	6.718734
53	1	0	3.666075	1.071727	5.999466
54	6	0	-5.655176	-1.339057	3.321448
55	6	0	-4.055365	-2.644879	4.591688
56	6	0	-6.043970	-0.529286	4.554260
57	1	0	-6.259139	-2.245974	3.258147
58	1	0	-5.822975	-0.782710	2.407967
59	6	0	-4.385007	-1.885384	5.873121
60	1	0	-4.732111	-3.492278	4.468644
61	1	0	-3.048887	-3.043416	4.612922
62	1	0	-7.091515	-0.244443	4.466811
63	1	0	-5.459266	0.394030	4.573282
64	1	0	-4.258856	-2.560119	6.718734
65	1	0	-3.666075	-1.071727	5.999466
66	6	0	-2.644879	4.055365	-4.591688
67	6	0	-1.339057	5.655176	-3.321448
68	6	0	-1.885384	4.385007	-5.873121

69	1	0	-3.492278	4.732111	-4.468644
70	1	0	-3.043416	3.048887	-4.612922
71	6	0	-0.529286	6.043970	-4.554260
72	1	0	-2.245974	6.259139	-3.258147
73	1	0	-0.782710	5.822975	-2.407967
74	1	0	-2.560119	4.258856	-6.718734
75	1	0	-1.071727	3.666075	-5.999466
76	1	0	-0.244443	7.091515	-4.466811
77	1	0	0.394030	5.459266	-4.573282
78	6	0	2.644879	-4.055365	-4.591688
79	6	0	1.339057	-5.655176	-3.321448
80	6	0	1.885384	-4.385007	-5.873121
81	1	0	3.492278	-4.732111	-4.468644
82	1	0	3.043416	-3.048887	-4.612922
83	6	0	0.529286	-6.043970	-4.554260
84	1	0	2.245974	-6.259139	-3.258147
85	1	0	0.782710	-5.822975	-2.407967
86	1	0	2.560119	-4.258856	-6.718734
87	1	0	1.071727	-3.666075	-5.999466
88	1	0	0.244443	-7.091515	-4.466811
89	1	0	-0.394030	-5.459266	-4.573282
90	7	0	4.252123	1.771107	3.424215
91	7	0	1.771107	-4.252123	-3.424215
92	7	0	-1.771107	4.252123	-3.424215
93	7	0	-4.252123	-1.771107	3.424215
94	6	0	5.807190	1.334164	5.828847
95	6	0	-1.334164	5.807190	-5.828847
96	6	0	1.334164	-5.807190	-5.828847
97	6	0	-5.807190	-1.334164	5.828847
98	1	0	5.999440	0.717722	6.704753
99	1	0	6.516796	2.164214	5.868082
100	1	0	0.717722	-5.999440	-6.704753
101	1	0	2.164214	-6.516796	-5.868082
102	1	0	-6.516796	-2.164214	5.868082
103	1	0	-5.999440	-0.717722	6.704753
104	1	0	-2.164214	6.516796	-5.868082
105	1	0	-0.717722	5.999440	-6.704753

TRICATION 3 E(RwB97XD) = -1862.99984001 a.u.

Center Number	Atomic Number	Atomic Type	Coordinates (Angstroms) X	Y	Z

1	5	0	0.000000	0.000000	0.000000
2	7	0	-1.181100	-0.419695	0.937942
3	6	0	-0.934883	-1.256250	1.989790
4	6	0	-2.467732	-0.000000	0.786237
5	6	0	-1.888413	-1.692231	2.844109
6	1	0	0.091045	-1.569608	2.117551
7	6	0	-3.483057	-0.386456	1.595230
8	1	0	-2.660116	0.691125	-0.017968
9	6	0	-3.246938	-1.278199	2.683854
10	1	0	-1.600627	-2.359327	3.640642
11	1	0	-4.467133	0.011748	1.408311
12	7	0	0.419695	-1.181100	-0.937942
13	6	0	1.256250	-0.934883	-1.989790
14	6	0	-0.000000	-2.467732	-0.786237
15	6	0	1.692231	-1.888413	-2.844109
16	1	0	1.569608	0.091045	-2.117551
17	6	0	0.386456	-3.483057	-1.595230
18	1	0	-0.691125	-2.660116	0.017968
19	6	0	1.278199	-3.246938	-2.683854
20	1	0	2.359327	-1.600627	-3.640642
21	1	0	-0.011748	-4.467133	-1.408311
22	7	0	1.181100	0.419695	0.937942
23	6	0	2.467732	-0.000000	0.786237
24	6	0	0.934883	1.256250	1.989790
25	6	0	3.483057	0.386456	1.595230
26	1	0	2.660116	-0.691125	-0.017968
27	6	0	1.888413	1.692231	2.844109
28	1	0	-0.091045	1.569608	2.117551
29	6	0	3.246938	1.278199	2.683854
30	1	0	4.467133	-0.011748	1.408311
31	1	0	1.600627	2.359327	3.640642
32	7	0	-0.419695	1.181100	-0.937942
33	6	0	0.000000	2.467732	-0.786237
34	6	0	-1.256250	0.934883	-1.989790
35	6	0	-0.386456	3.483057	-1.595230
36	1	0	0.691125	2.660116	0.017968
37	6	0	-1.692231	1.888413	-2.844109
38	1	0	-1.569608	-0.091045	-2.117551
39	6	0	-1.278199	3.246938	-2.683854
40	1	0	0.011748	4.467133	-1.408311
41	1	0	-2.359327	1.600627	-3.640642
42	6	0	-2.543234	3.996772	-4.664882
43	6	0	-1.331287	5.628046	-3.321159

44	6	0	-2.466857	5.326384	-5.405647
45	1	0	-2.176445	3.161710	-5.259437
46	1	0	-3.560464	3.775797	-4.334586
47	6	0	-2.270165	6.343743	-4.284752
48	1	0	-1.473762	5.935209	-2.286419
49	1	0	-0.283786	5.775374	-3.592736
50	1	0	-3.358803	5.510828	-5.997120
51	1	0	-1.610115	5.329697	-6.079171
52	1	0	-3.218415	6.557975	-3.792195
53	1	0	-1.852688	7.284419	-4.631483
54	6	0	-3.996772	-2.543234	4.664882
55	6	0	-5.628046	-1.331287	3.321159
56	6	0	-5.326384	-2.466857	5.405647
57	1	0	-3.775797	-3.560464	4.334586
58	1	0	-3.161710	-2.176445	5.259437
59	6	0	-6.343743	-2.270165	4.284752
60	1	0	-5.775374	-0.283786	3.592736
61	1	0	-5.935209	-1.473762	2.286419
62	1	0	-5.329697	-1.610115	6.079171
63	1	0	-5.510828	-3.358803	5.997120
64	1	0	-6.557975	-3.218415	3.792195
65	1	0	-7.284419	-1.852688	4.631483
66	6	0	5.628046	1.331287	3.321159
67	6	0	3.996772	2.543234	4.664882
68	6	0	6.343743	2.270165	4.284752
69	1	0	5.775374	0.283786	3.592736
70	1	0	5.935209	1.473762	2.286419
71	6	0	5.326384	2.466857	5.405647
72	1	0	3.775797	3.560464	4.334586
73	1	0	3.161710	2.176445	5.259437
74	1	0	7.284419	1.852688	4.631483
75	1	0	6.557975	3.218415	3.792195
76	1	0	5.510828	3.358803	5.997120
77	1	0	5.329697	1.610115	6.079171
78	6	0	2.543234	-3.996772	-4.664882
79	6	0	1.331287	-5.628046	-3.321159
80	6	0	2.466857	-5.326384	-5.405647
81	1	0	2.176445	-3.161710	-5.259437
82	1	0	3.560464	-3.775797	-4.334586
83	6	0	2.270165	-6.343743	-4.284752
84	1	0	1.473762	-5.935209	-2.286419
85	1	0	0.283786	-5.775374	-3.592736
86	1	0	3.358803	-5.510828	-5.997120

87	1	0	1.610115	-5.329697	-6.079171
88	1	0	3.218415	-6.557975	-3.792195
89	1	0	1.852688	-7.284419	-4.631483
90	7	0	-4.204838	-1.681966	3.485755
91	7	0	1.681966	-4.204838	-3.485755
92	7	0	4.204838	1.681966	3.485755
93	7	0	-1.681966	4.204838	-3.485755

TRICATION 4 E(RwB97XD) = -2163.79431553 a.u.

Center Number	Atomic Number	Atomic Type	Coordinates (Angstroms) X	Y	Z
1	5	0	0.000000	0.000000	0.000000
2	7	0	1.178662	0.425707	0.936889
3	6	0	0.935524	1.263054	1.986785
4	6	0	2.463779	0.000000	0.803719
5	6	0	1.886244	1.697310	2.844689
6	1	0	-0.090047	1.576474	2.118284
7	6	0	3.473869	0.383552	1.620331
8	1	0	2.663802	-0.697201	0.006435
9	6	0	3.253083	1.291276	2.703589
10	1	0	1.567200	2.347350	3.640633
11	1	0	4.444428	-0.037463	1.424812
12	7	0	-0.425707	1.178662	-0.936889
13	6	0	-1.263054	0.935524	-1.986785
14	6	0	0.000000	2.463779	-0.803719
15	6	0	-1.697310	1.886244	-2.844689
16	1	0	-1.576474	-0.090047	-2.118284
17	6	0	-0.383552	3.473869	-1.620331
18	1	0	0.697201	2.663802	-0.006435
19	6	0	-1.291276	3.253083	-2.703589
20	1	0	-2.347350	1.567200	-3.640633
21	1	0	0.037463	4.444428	-1.424812
22	7	0	-1.178662	-0.425707	0.936889
23	6	0	-2.463779	0.000000	0.803719
24	6	0	-0.935524	-1.263054	1.986785
25	6	0	-3.473869	-0.383552	1.620331
26	1	0	-2.663802	0.697201	0.006435
27	6	0	-1.886244	-1.697310	2.844689
28	1	0	0.090047	-1.576474	2.118284
29	6	0	-3.253083	-1.291276	2.703589
30	1	0	-4.444428	0.037463	1.424812

31	1	0	-1.567200	-2.347350	3.640633
32	7	0	0.425707	-1.178662	-0.936889
33	6	0	-0.000000	-2.463779	-0.803719
34	6	0	1.263054	-0.935524	-1.986785
35	6	0	0.383552	-3.473869	-1.620331
36	1	0	-0.697201	-2.663802	-0.006435
37	6	0	1.697310	-1.886244	-2.844689
38	1	0	1.576474	0.090047	-2.118284
39	6	0	1.291276	-3.253083	-2.703589
40	1	0	-0.037463	-4.444428	-1.424812
41	1	0	2.347350	-1.567200	-3.640633
42	6	0	4.003724	2.579331	4.674260
43	6	0	5.614880	1.256591	3.424071
44	6	0	5.027205	3.709712	4.679781
45	1	0	4.122953	1.970537	5.572518
46	1	0	3.006707	3.001880	4.672833
47	6	0	6.557984	2.453064	3.492781
48	1	0	5.802783	0.589602	4.267563
49	1	0	5.797160	0.713178	2.505394
50	1	0	4.919052	4.285065	5.596293
51	1	0	4.849113	4.376317	3.825785
52	1	0	7.584787	2.096714	3.529415
53	1	0	6.435698	3.074257	2.595902
54	6	0	-4.003724	-2.579331	4.674260
55	6	0	-5.614880	-1.256591	3.424071
56	6	0	-5.027205	-3.709712	4.679781
57	1	0	-4.122953	-1.970537	5.572518
58	1	0	-3.006707	-3.001880	4.672833
59	6	0	-6.557984	-2.453064	3.492781
60	1	0	-5.802783	-0.589602	4.267563
61	1	0	-5.797160	-0.713178	2.505394
62	1	0	-4.919052	-4.285065	5.596293
63	1	0	-4.849113	-4.376317	3.825785
64	1	0	-7.584787	-2.096714	3.529415
65	1	0	-6.435698	-3.074257	2.595902
66	6	0	-1.256591	5.614880	-3.424071
67	6	0	-2.579331	4.003724	-4.674260
68	6	0	-2.453064	6.557984	-3.492781
69	1	0	-0.589602	5.802783	-4.267563
70	1	0	-0.713178	5.797160	-2.505394
71	6	0	-3.709712	5.027205	-4.679781
72	1	0	-1.970537	4.122953	-5.572518
73	1	0	-3.001880	3.006707	-4.672833

74	1	0	-2.096714	7.584787	-3.529415
75	1	0	-3.074257	6.435698	-2.595902
76	1	0	-4.285065	4.919052	-5.596293
77	1	0	-4.376317	4.849113	-3.825785
78	6	0	1.256591	-5.614880	-3.424071
79	6	0	2.579331	-4.003724	-4.674260
80	6	0	2.453064	-6.557984	-3.492781
81	1	0	0.589602	-5.802783	-4.267563
82	1	0	0.713178	-5.797160	-2.505394
83	6	0	3.709712	-5.027205	-4.679781
84	1	0	1.970537	-4.122953	-5.572518
85	1	0	3.001880	-3.006707	-4.672833
86	1	0	2.096714	-7.584787	-3.529415
87	1	0	3.074257	-6.435698	-2.595902
88	1	0	4.285065	-4.919052	-5.596293
89	1	0	4.376317	-4.849113	-3.825785
90	7	0	4.215358	1.704826	3.509311
91	7	0	1.704826	-4.215358	-3.509311
92	7	0	-1.704826	4.215358	-3.509311
93	7	0	-4.215358	-1.704826	3.509311
94	8	0	-3.206514	6.332327	-4.650174
95	8	0	3.206514	-6.332327	-4.650174
96	8	0	-6.332327	-3.206514	4.650174
97	8	0	6.332327	3.206514	4.650174

TRICATION 5 E(RwB97XD) = -2172.77690483 a.u.

Center Number	Atomic Number	Atomic Type	Coordinates (Angstroms) X	Y	Z
1	5	0	0.000000	0.000000	0.000000
2	7	0	1.167758	0.452994	0.937715
3	6	0	0.893531	1.278876	1.987603
4	6	0	2.462803	0.071966	0.781146
5	6	0	1.824108	1.739052	2.859999
6	1	0	-0.143633	1.556857	2.113054
7	6	0	3.481430	0.490930	1.576553
8	1	0	2.669888	-0.609620	-0.028673
9	6	0	3.190964	1.362107	2.670031
10	7	0	4.143465	1.794657	3.482265
11	6	0	1.463655	2.625539	4.017061
12	1	0	0.551080	3.180313	3.804044
13	1	0	1.264206	2.009122	4.897506

14	6	0	2.625657	3.565607	4.312666
15	1	0	2.776689	4.249064	3.475339
16	1	0	2.419353	4.171727	5.191719
17	6	0	3.888263	2.763587	4.554037
18	1	0	3.834730	2.223868	5.502721
19	1	0	4.754989	3.421508	4.603615
20	6	0	4.899994	0.055298	1.342005
21	1	0	4.923145	-0.910543	0.839322
22	1	0	5.395184	0.771620	0.681282
23	6	0	5.641109	-0.000000	2.671856
24	1	0	6.691995	-0.234812	2.519632
25	1	0	5.220757	-0.785023	3.302600
26	6	0	5.533441	1.336421	3.377574
27	1	0	6.113333	2.103265	2.858158
28	1	0	5.923924	1.265353	4.391877
29	7	0	-0.452994	1.167758	-0.937715
30	6	0	-1.278876	0.893531	-1.987603
31	6	0	-0.071966	2.462803	-0.781146
32	6	0	-1.739052	1.824108	-2.859999
33	1	0	-1.556857	-0.143633	-2.113054
34	6	0	-0.490930	3.481430	-1.576553
35	1	0	0.609620	2.669888	0.028673
36	6	0	-1.362107	3.190964	-2.670031
37	6	0	-2.625539	1.463655	-4.017061
38	6	0	-0.055298	4.899994	-1.342005
39	7	0	-1.794657	4.143465	-3.482265
40	1	0	-2.009122	1.264206	-4.897506
41	1	0	-3.180313	0.551080	-3.804044
42	6	0	-3.565607	2.625657	-4.312666
43	1	0	-0.771620	5.395184	-0.681282
44	1	0	0.910543	4.923145	-0.839322
45	6	0	0.000000	5.641109	-2.671856
46	6	0	-2.763587	3.888263	-4.554037
47	6	0	-1.336421	5.533441	-3.377574
48	1	0	-4.249064	2.776689	-3.475339
49	1	0	-4.171727	2.419353	-5.191719
50	1	0	0.785023	5.220757	-3.302600
51	1	0	0.234812	6.691995	-2.519632
52	1	0	-3.421508	4.754989	-4.603615
53	1	0	-2.223868	3.834730	-5.502721
54	1	0	-1.265353	5.923924	-4.391877
55	1	0	-2.103265	6.113333	-2.858158
56	7	0	0.452994	-1.167758	-0.937715

57	6	0	1.278876	-0.893531	-1.987603
58	6	0	0.071966	-2.462803	-0.781146
59	6	0	1.739052	-1.824108	-2.859999
60	1	0	1.556857	0.143633	-2.113054
61	6	0	0.490930	-3.481430	-1.576553
62	1	0	-0.609620	-2.669888	0.028673
63	6	0	1.362107	-3.190964	-2.670031
64	6	0	2.625539	-1.463655	-4.017061
65	6	0	0.055298	-4.899994	-1.342005
66	7	0	1.794657	-4.143465	-3.482265
67	1	0	2.009122	-1.264206	-4.897506
68	1	0	3.180313	-0.551080	-3.804044
69	6	0	3.565607	-2.625657	-4.312666
70	1	0	0.771620	-5.395184	-0.681282
71	1	0	-0.910543	-4.923145	-0.839322
72	6	0	-0.000000	-5.641109	-2.671856
73	6	0	2.763587	-3.888263	-4.554037
74	6	0	1.336421	-5.533441	-3.377574
75	1	0	4.171727	-2.419353	-5.191719
76	1	0	4.249064	-2.776689	-3.475339
77	1	0	-0.234812	-6.691995	-2.519632
78	1	0	-0.785023	-5.220757	-3.302600
79	1	0	3.421508	-4.754989	-4.603615
80	1	0	2.223868	-3.834730	-5.502721
81	1	0	1.265353	-5.923924	-4.391877
82	1	0	2.103265	-6.113333	-2.858158
83	7	0	-1.167758	-0.452994	0.937715
84	6	0	-2.462803	-0.071966	0.781146
85	6	0	-0.893531	-1.278876	1.987603
86	6	0	-3.481430	-0.490930	1.576553
87	1	0	-2.669888	0.609620	-0.028673
88	6	0	-1.824108	-1.739052	2.859999
89	1	0	0.143633	-1.556857	2.113054
90	6	0	-3.190964	-1.362107	2.670031
91	6	0	-4.899994	-0.055298	1.342005
92	6	0	-1.463655	-2.625539	4.017061
93	7	0	-4.143465	-1.794657	3.482265
94	1	0	-4.923145	0.910543	0.839322
95	1	0	-5.395184	-0.771620	0.681282
96	6	0	-5.641109	0.000000	2.671856
97	1	0	-0.551080	-3.180313	3.804044
98	1	0	-1.264206	-2.009122	4.897506
99	6	0	-2.625657	-3.565607	4.312666

100	6	0	-5.533441	-1.336421	3.377574
101	6	0	-3.888263	-2.763587	4.554037
102	1	0	-6.691995	0.234812	2.519632
103	1	0	-5.220757	0.785023	3.302600
104	1	0	-2.419353	-4.171727	5.191719
105	1	0	-2.776689	-4.249064	3.475339
106	1	0	-6.113333	-2.103265	2.858158
107	1	0	-5.923924	-1.265353	4.391877
108	1	0	-3.834730	-2.223868	5.502721
109	1	0	-4.754989	-3.421508	4.603615

TRICATION 6 E(RwB97XD) = -1174.48808079 a.u.

Center Number	Atomic Number	Atomic Type	Coordinates (Angstroms) X	Y	Z
1	5	0	0.000000	0.000000	0.000000
2	7	0	-0.435638	1.180841	0.948306
3	6	0	-1.287774	0.908256	1.972464
4	6	0	-0.000000	2.454924	0.817245
5	6	0	-1.725672	1.869814	2.837860
6	1	0	-1.610684	-0.117577	2.075134
7	6	0	-0.404229	3.457812	1.659467
8	1	0	0.698083	2.660352	0.022636
9	6	0	-1.293059	3.197189	2.705413
10	1	0	-2.408812	1.589604	3.627515
11	1	0	-0.018995	4.455011	1.500216
12	7	0	-1.180841	-0.435638	-0.948306
13	6	0	-0.908256	-1.287774	-1.972464
14	6	0	-2.454924	-0.000000	-0.817245
15	6	0	-1.869814	-1.725672	-2.837860
16	1	0	0.117577	-1.610684	-2.075134
17	6	0	-3.457812	-0.404229	-1.659467
18	1	0	-2.660352	0.698083	-0.022636
19	6	0	-3.197189	-1.293059	-2.705413
20	1	0	-1.589604	-2.408812	-3.627515
21	1	0	-4.455011	-0.018995	-1.500216
22	7	0	0.435638	-1.180841	0.948306
23	6	0	-0.000000	-2.454924	0.817245
24	6	0	1.287774	-0.908256	1.972464
25	6	0	0.404229	-3.457812	1.659467
26	1	0	-0.698083	-2.660352	0.022636
27	6	0	1.725672	-1.869814	2.837860

28	1	0	1.610684	0.117577	2.075134
29	6	0	1.293059	-3.197189	2.705413
30	1	0	0.018995	-4.455011	1.500216
31	1	0	2.408812	-1.589604	3.627515
32	7	0	1.180841	0.435638	-0.948306
33	6	0	2.454924	-0.000000	-0.817245
34	6	0	0.908256	1.287774	-1.972464
35	6	0	3.457812	0.404229	-1.659467
36	1	0	2.660352	-0.698083	-0.022636
37	6	0	1.869814	1.725672	-2.837860
38	1	0	-0.117577	1.610684	-2.075134
39	6	0	3.197189	1.293059	-2.705413
40	1	0	4.455011	0.018995	-1.500216
41	1	0	1.589604	2.408812	-3.627515
42	6	0	4.256766	1.734703	-3.647781
43	1	0	4.104993	2.766242	-3.960911
44	1	0	5.250992	1.623773	-3.222502
45	1	0	4.209949	1.116258	-4.549427
46	6	0	-1.734703	4.256766	3.647781
47	1	0	-2.766242	4.104993	3.960911
48	1	0	-1.116258	4.209949	4.549427
49	1	0	-1.623773	5.250992	3.222502
50	6	0	1.734703	-4.256766	3.647781
51	1	0	1.116258	-4.209949	4.549427
52	1	0	1.623773	-5.250992	3.222502
53	1	0	2.766242	-4.104993	3.960911
54	6	0	-4.256766	-1.734703	-3.647781
55	1	0	-5.250992	-1.623773	-3.222502
56	1	0	-4.209949	-1.116258	-4.549427
57	1	0	-4.104993	-2.766242	-3.960911

TRICATION 7 E(RwB97XD) = -1017.15493299 a.u.

Center Number	Atomic Number	Atomic Type	Coordinates (Angstroms) X	Y	Z
1	5	0	0.000000	0.000000	0.000000
2	7	0	0.723450	1.032369	0.953650
3	6	0	-0.000000	1.579822	1.965823
4	6	0	2.025274	1.378889	0.834356
5	6	0	0.540244	2.481699	2.845423
6	1	0	-1.031326	1.269745	2.051384
7	6	0	2.622823	2.275432	1.689475

8	1	0	2.594316	0.913515	0.046898
9	6	0	1.876474	2.844148	2.710401
10	1	0	-0.077685	2.891261	3.631504
11	1	0	3.667236	2.516589	1.554110
12	1	0	2.326775	3.550979	3.394846
13	7	0	-1.032369	0.723450	-0.953650
14	6	0	-1.579822	-0.000000	-1.965823
15	6	0	-1.378889	2.025274	-0.834356
16	6	0	-2.481699	0.540244	-2.845423
17	1	0	-1.269745	-1.031326	-2.051384
18	6	0	-2.275432	2.622823	-1.689475
19	1	0	-0.913515	2.594316	-0.046898
20	6	0	-2.844148	1.876474	-2.710401
21	1	0	-2.891261	-0.077685	-3.631504
22	1	0	-2.516589	3.667236	-1.554110
23	1	0	-3.550979	2.326775	-3.394846
24	7	0	-0.723450	-1.032369	0.953650
25	6	0	-2.025274	-1.378889	0.834356
26	6	0	-0.000000	-1.579822	1.965823
27	6	0	-2.622823	-2.275432	1.689475
28	1	0	-2.594316	-0.913515	0.046898
29	6	0	-0.540244	-2.481699	2.845423
30	1	0	1.031326	-1.269745	2.051384
31	6	0	-1.876474	-2.844148	2.710401
32	1	0	-3.667236	-2.516589	1.554110
33	1	0	0.077685	-2.891261	3.631504
34	1	0	-2.326775	-3.550979	3.394846
35	7	0	1.032369	-0.723450	-0.953650
36	6	0	1.378889	-2.025274	-0.834356
37	6	0	1.579822	-0.000000	-1.965823
38	6	0	2.275432	-2.622823	-1.689475
39	1	0	0.913515	-2.594316	-0.046898
40	6	0	2.481699	-0.540244	-2.845423
41	1	0	1.269745	1.031326	-2.051384
42	6	0	2.844148	-1.876474	-2.710401
43	1	0	2.516589	-3.667236	-1.554110
44	1	0	2.891261	0.077685	-3.631504
45	1	0	3.550979	-2.326775	-3.394846

NMRs (pH stability study, compound $1Br_3$) Labelling keys

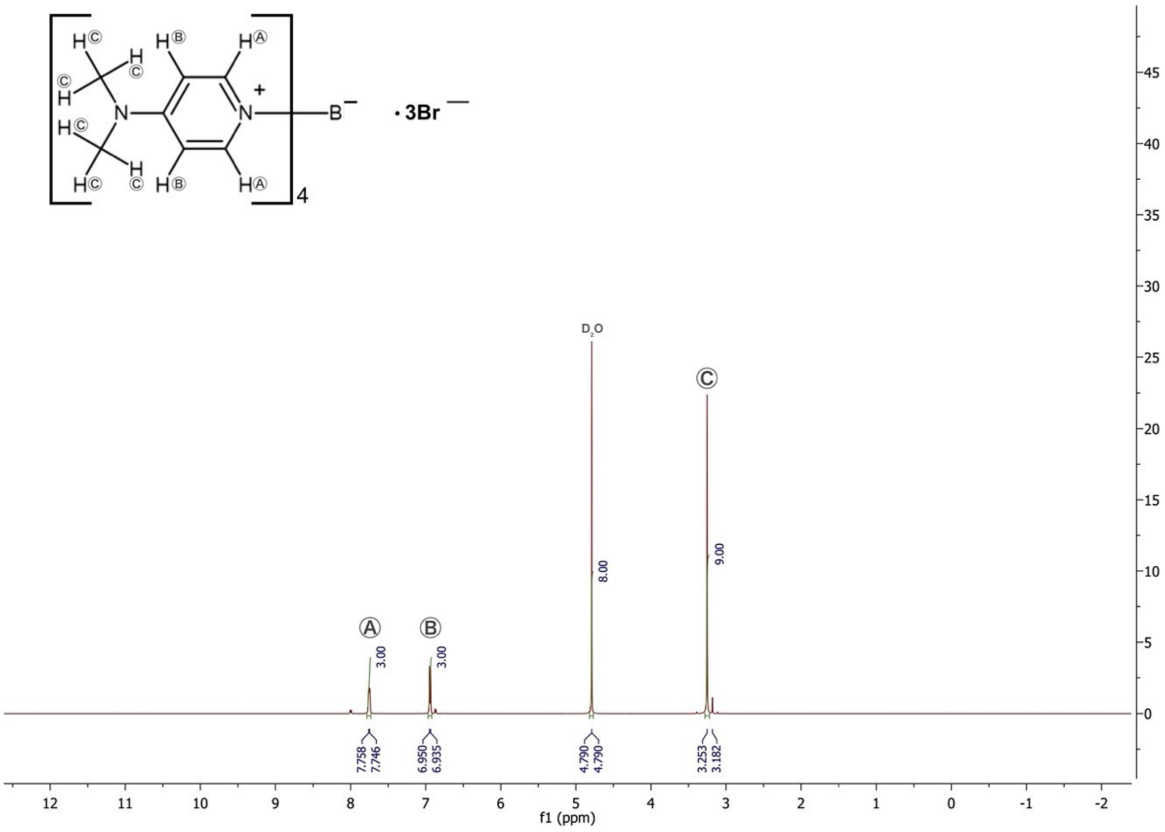

·3Br
4
D2O
A
B
C
3.00
3.00
8.00
9.00
7.758
7.746
6.950
6.935
4.790
4.790
3.253
3.182
f1 (ppm)

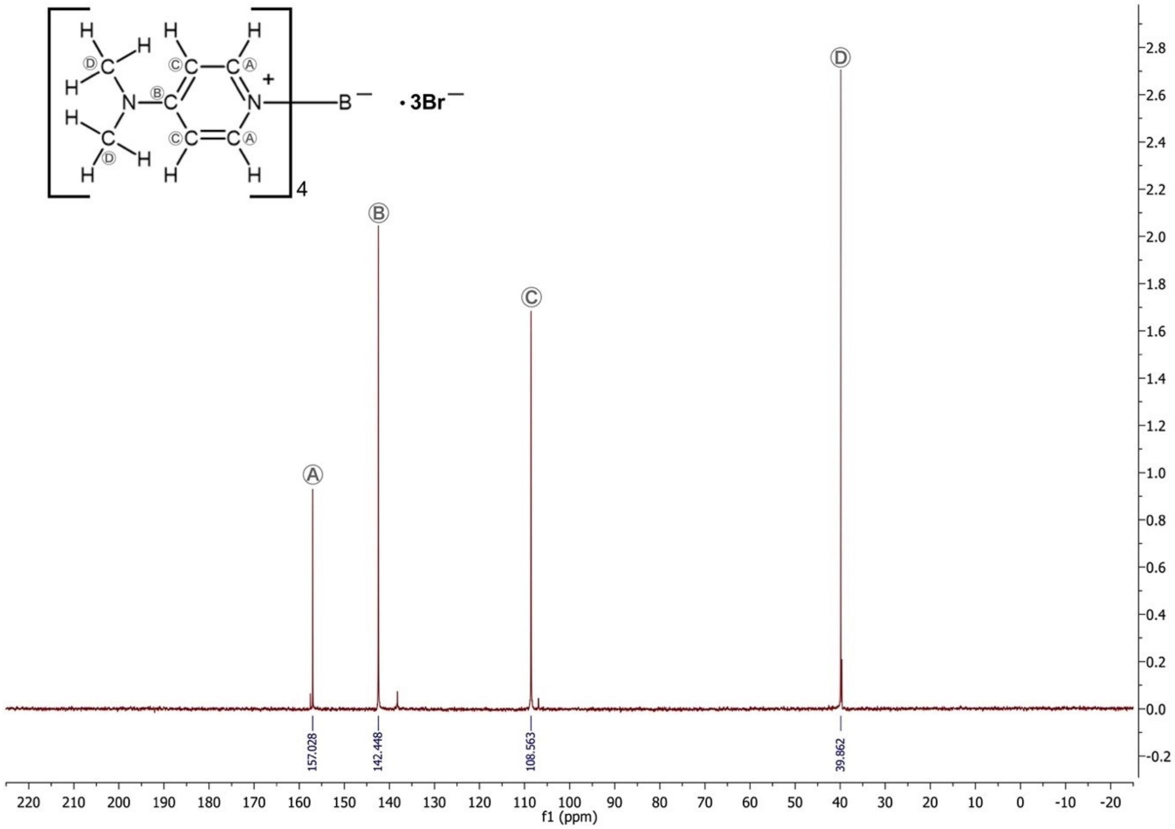

H
H
H
D
C
H
N
B
C
C
C
C
C
A
A
A
+
N
B
·3Br
4
B
C
D
157.028
142.448
108.563
39.862
f1 (ppm)

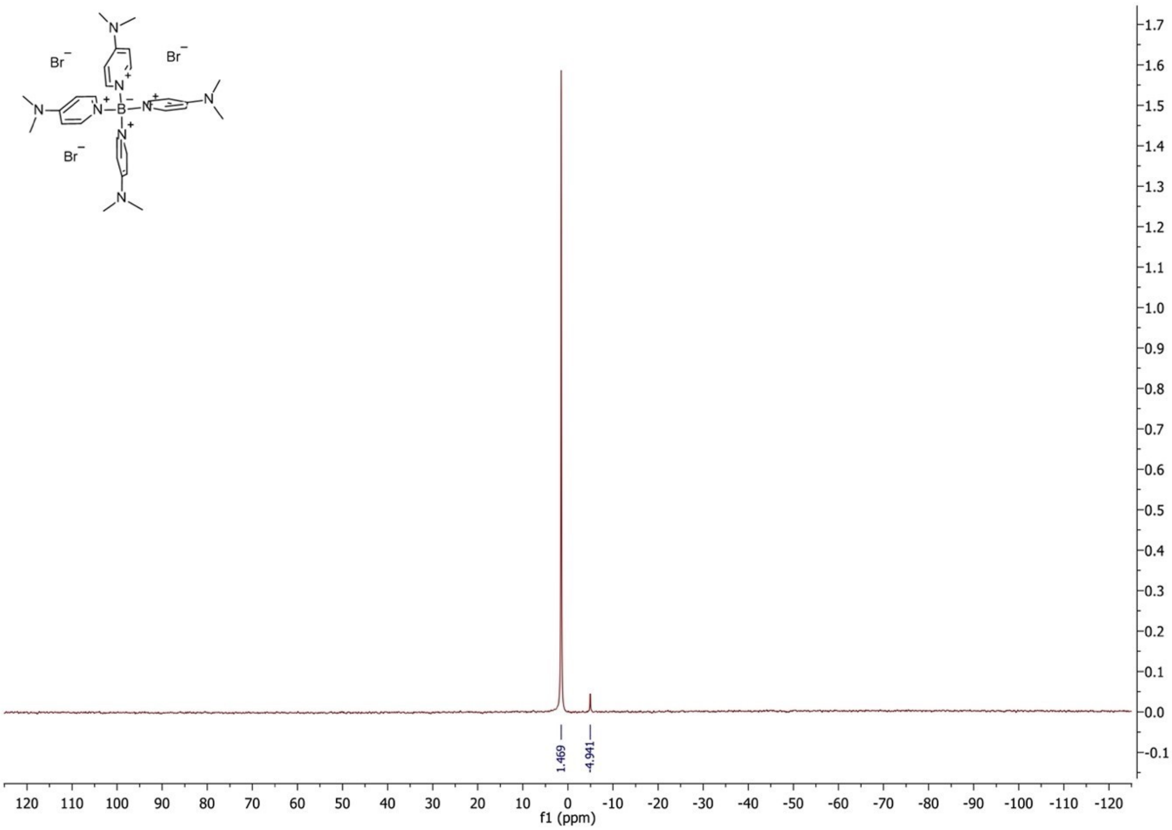
Br⁻
Br⁻
Br⁻
N
B
1.469
-4.941
f1 (ppm)

^{1}H-NMR, T = O

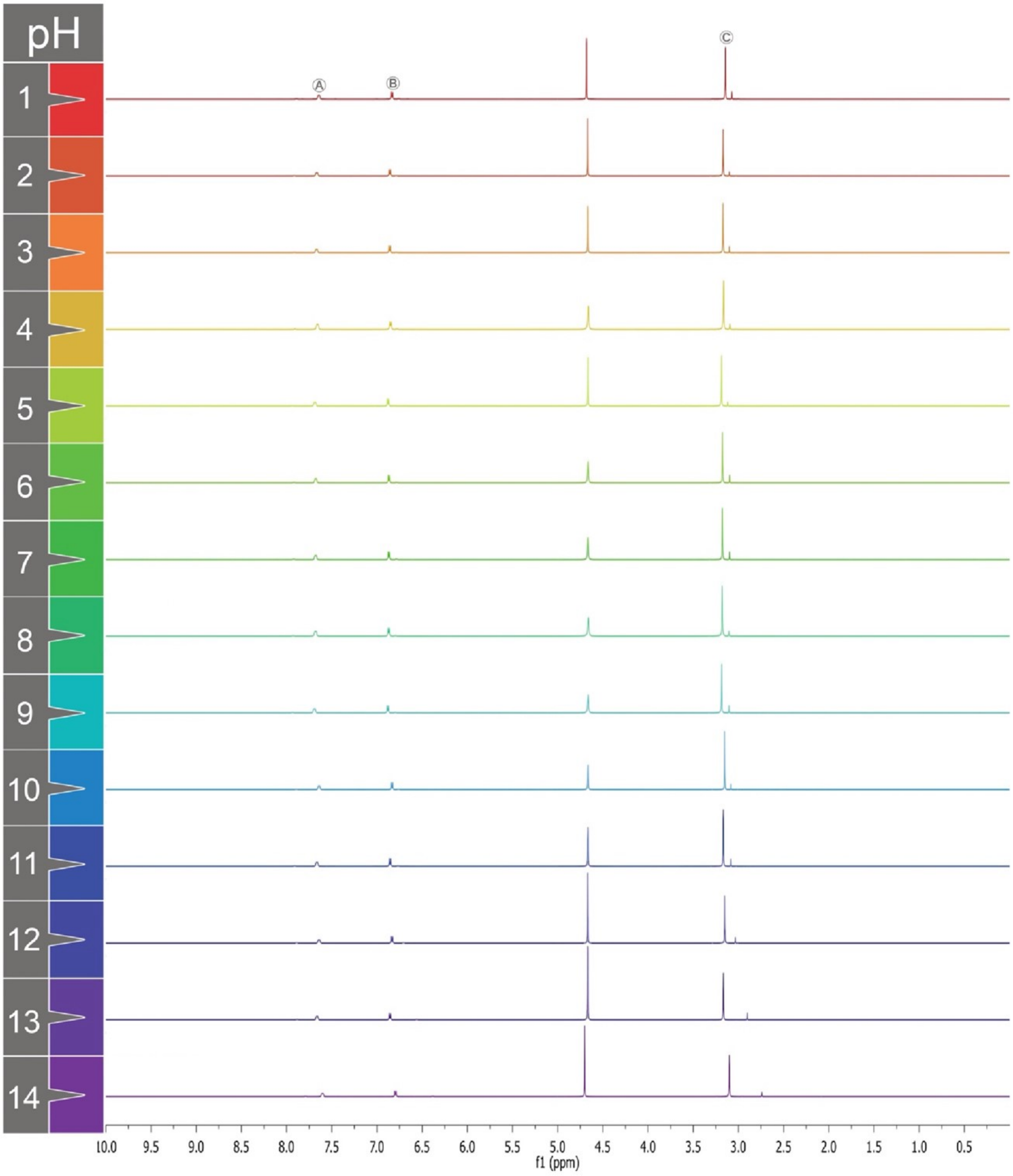

pH
1
2
3
4
5
6
7
8
9
10
11
12
13
14
A
B
C
10.0 9.5 9.0 8.5 8.0 7.5 7.0 6.5 6.0 5.5 5.0 4.5 4.0 3.5 3.0 2.5 2.0 1.5 1.0 0.5
f1 (ppm)

^{13}C-NMR, T = 0

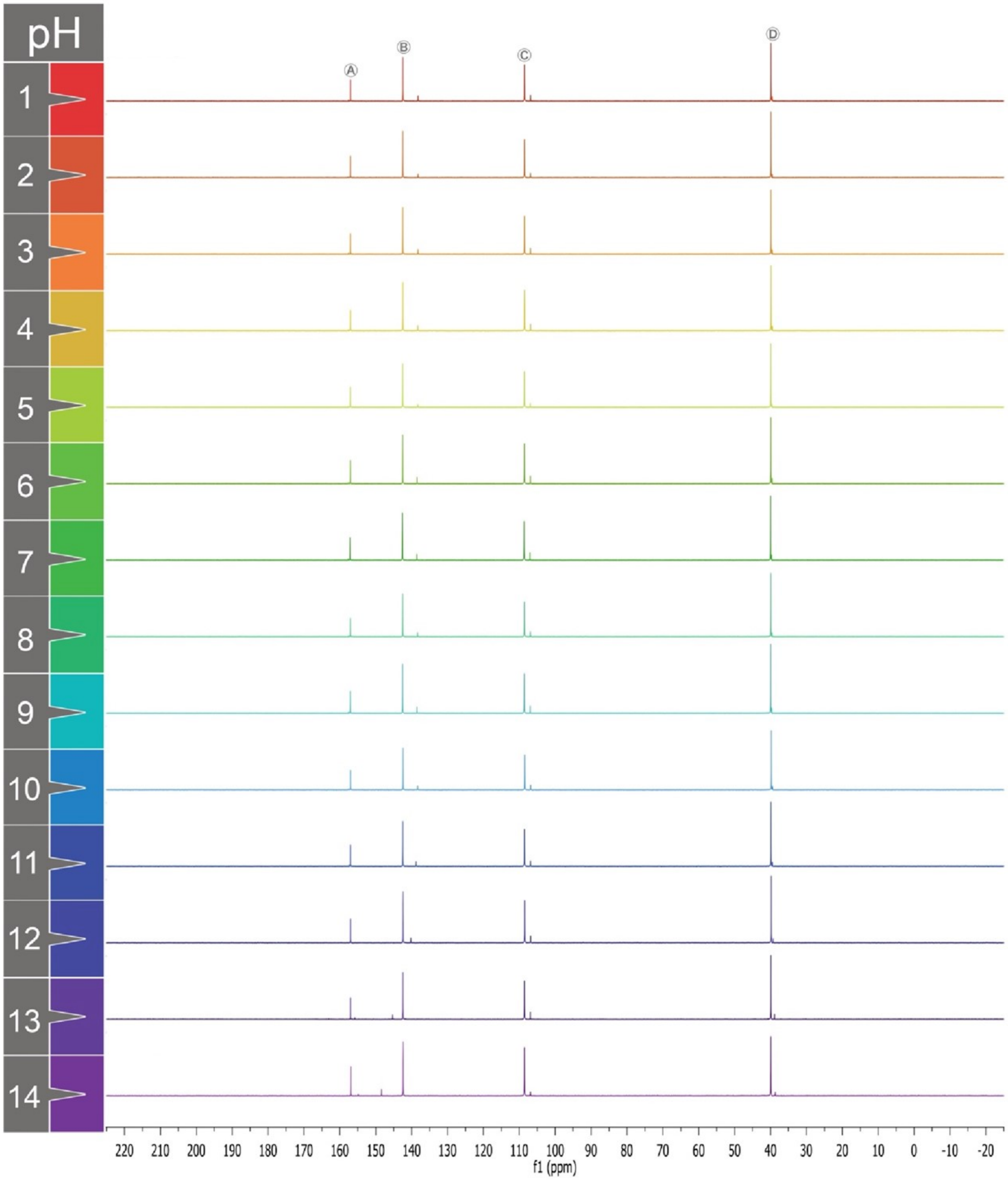

pH
1
2
3
4
5
6
7
8
9
10
11
12
13
14
A
B
C
D
220 210 200 190 180 170 160 150 140 130 120 110 100 90 80 70 60 50 40 30 20 10 0 -10 -20
f1 (ppm)

[10]B-NMR, T = 0

No baseline correction applied.

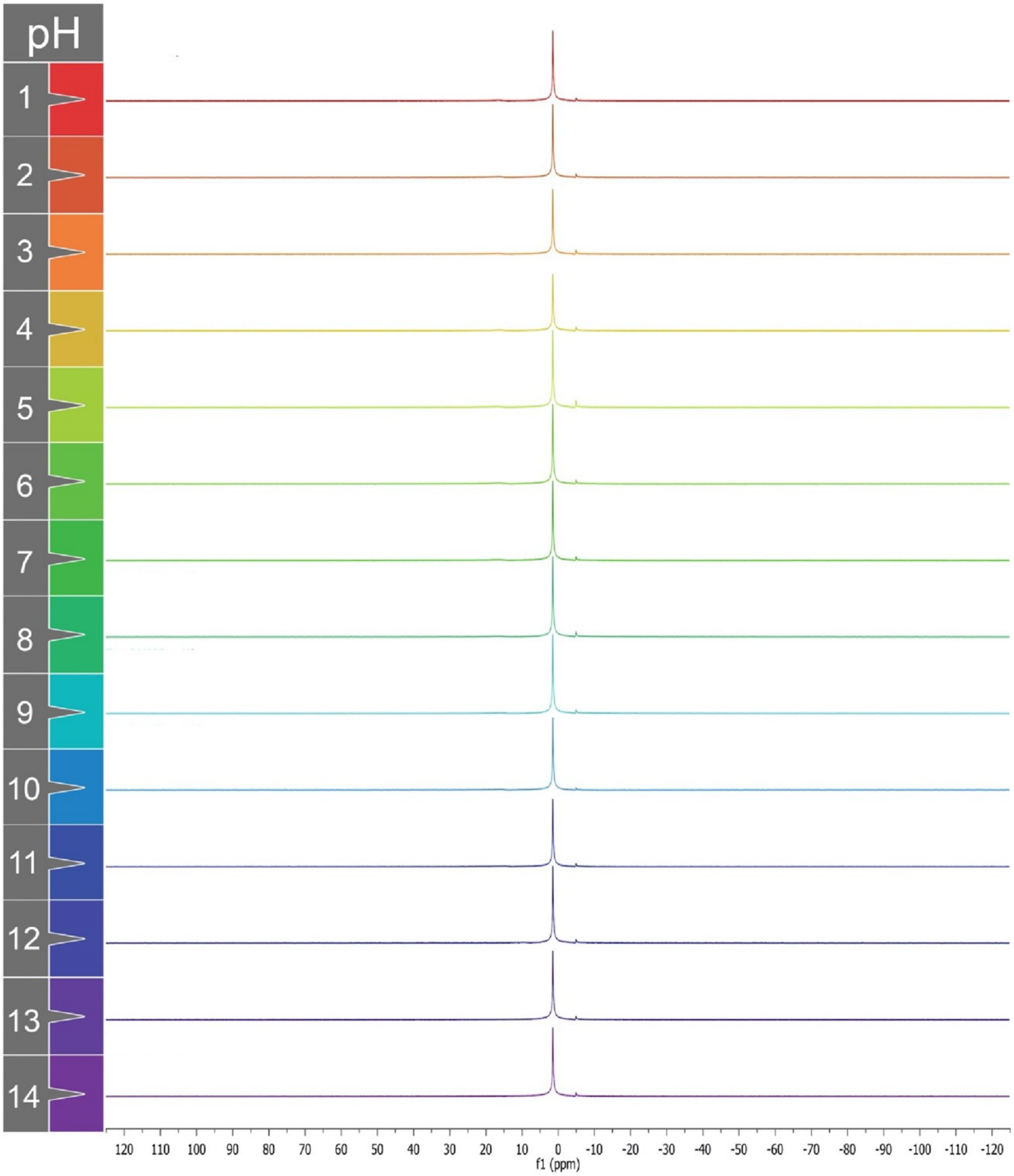

^{1}H-NMR, T = 1 week

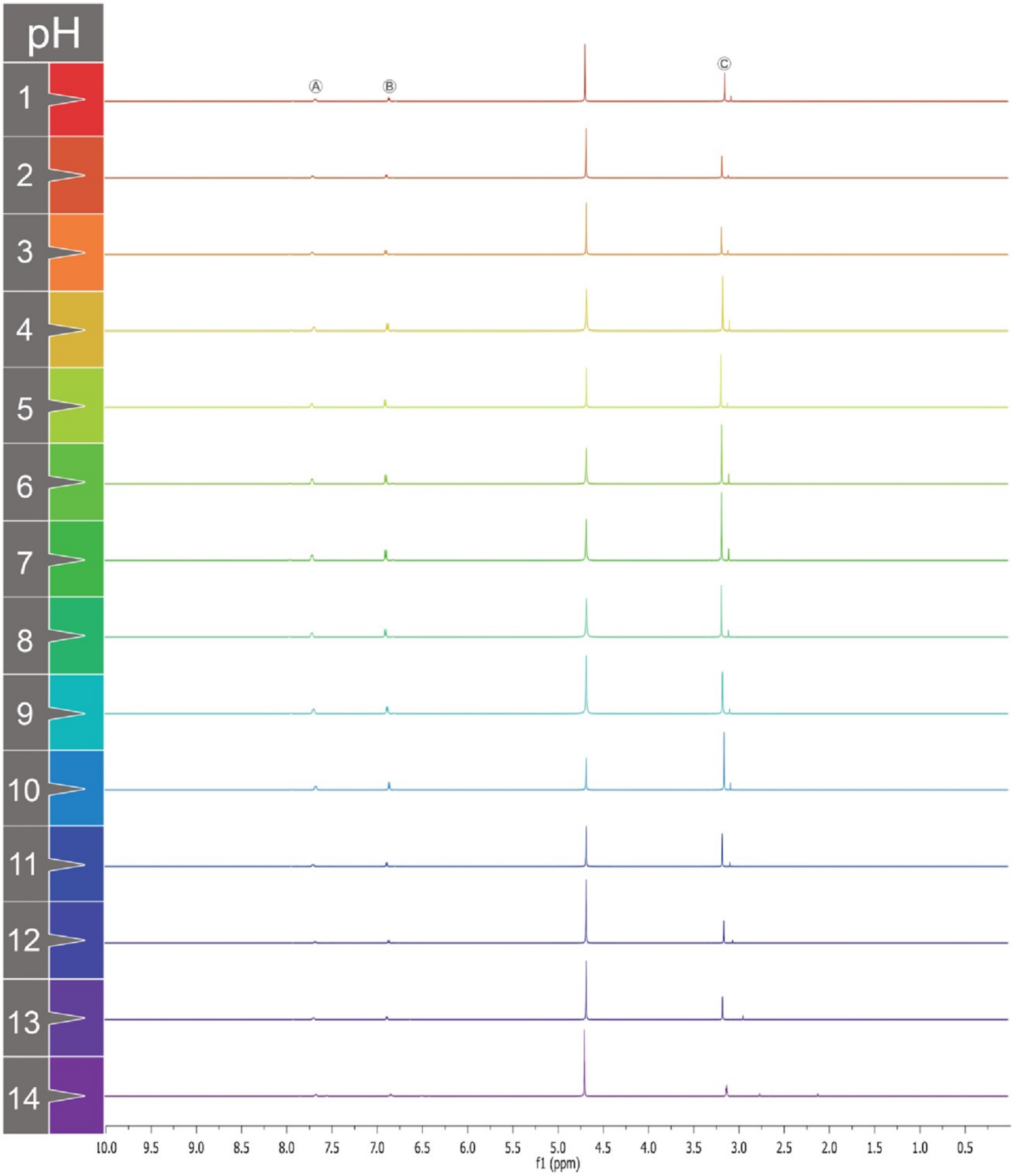

^{13}C-NMR, T = 1 week

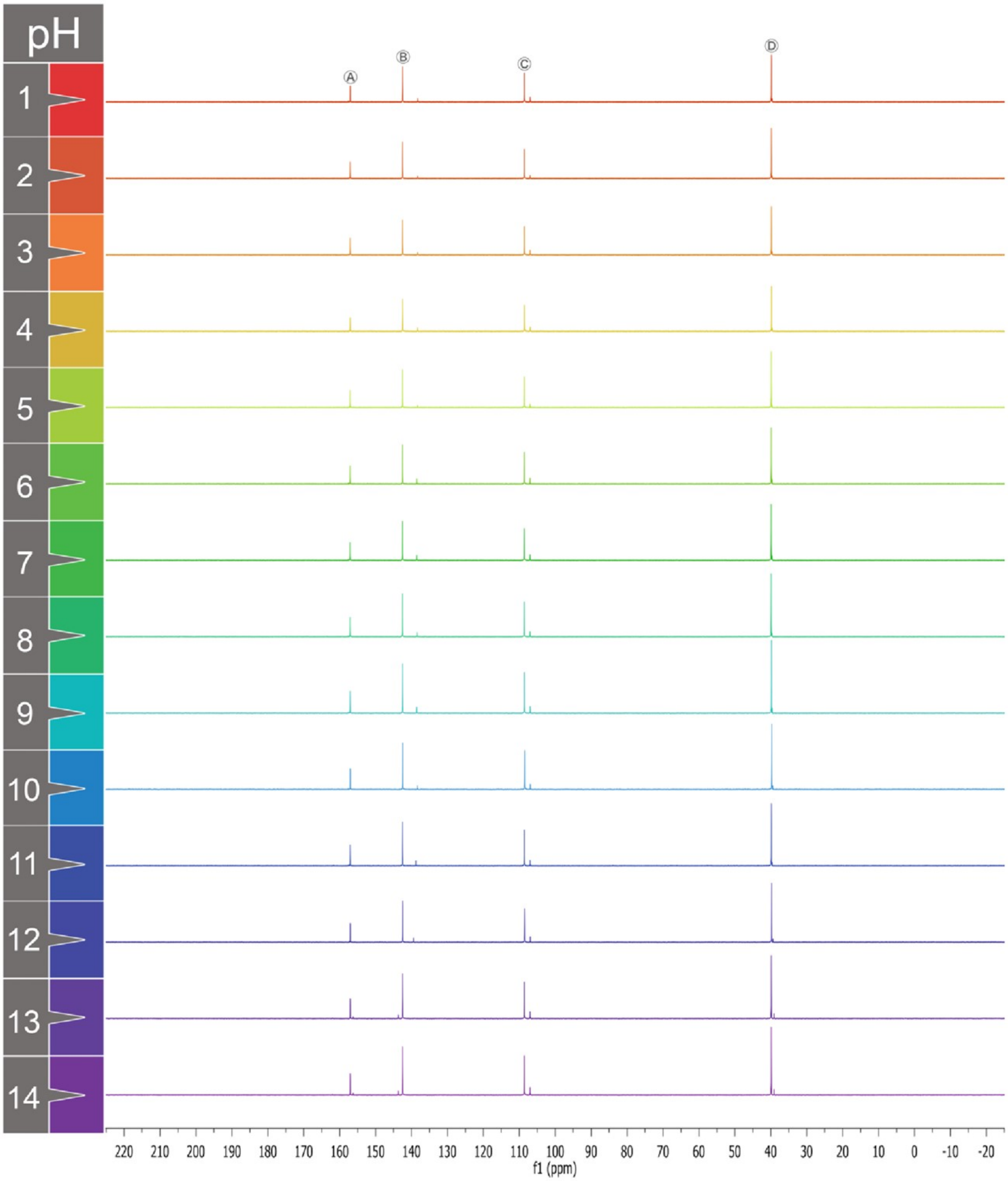

^{10}B-NMR, T = 1 week

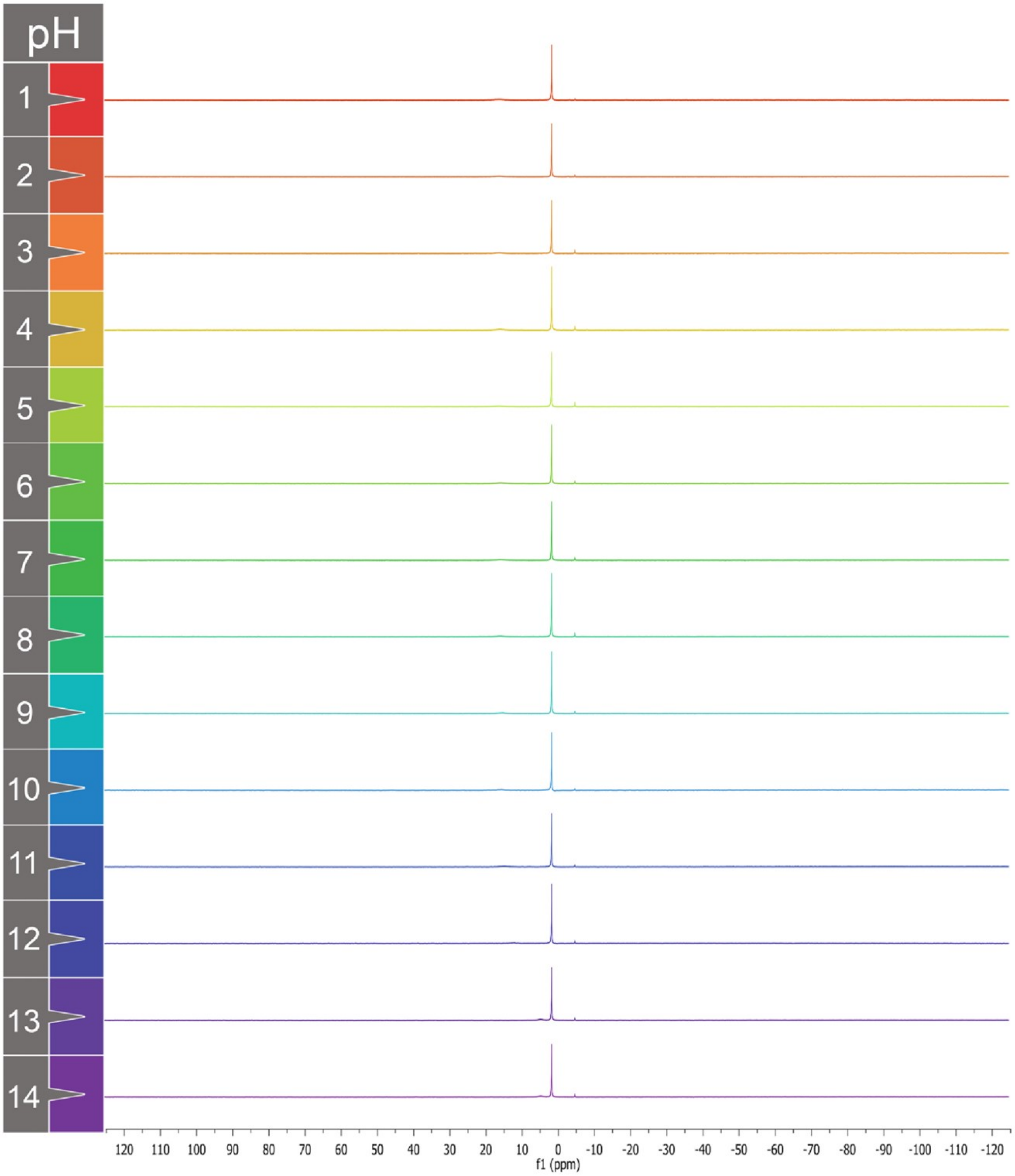

^{1}H-NMR, T = 4 months

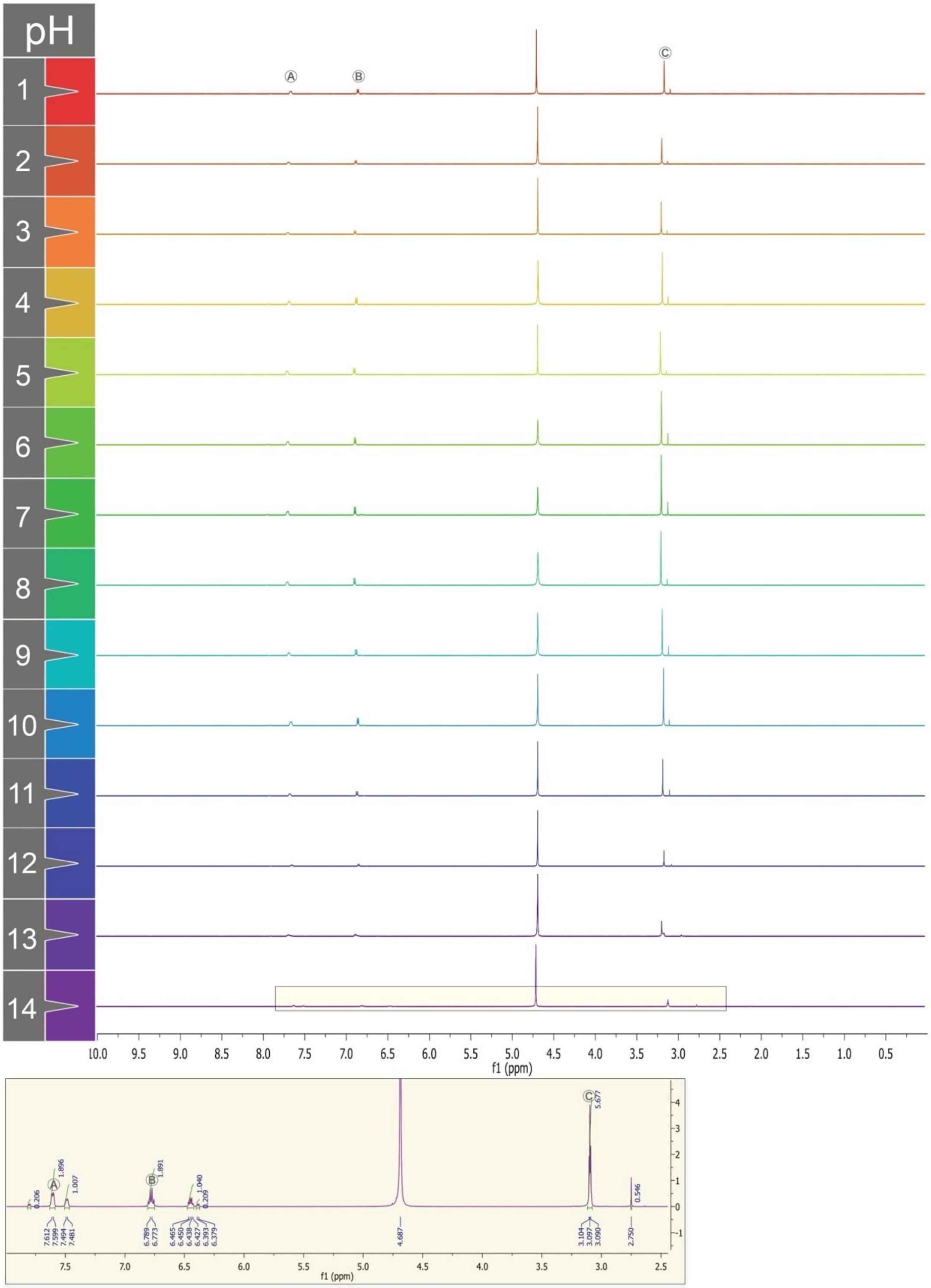

^{13}C-NMR, T = 4 months

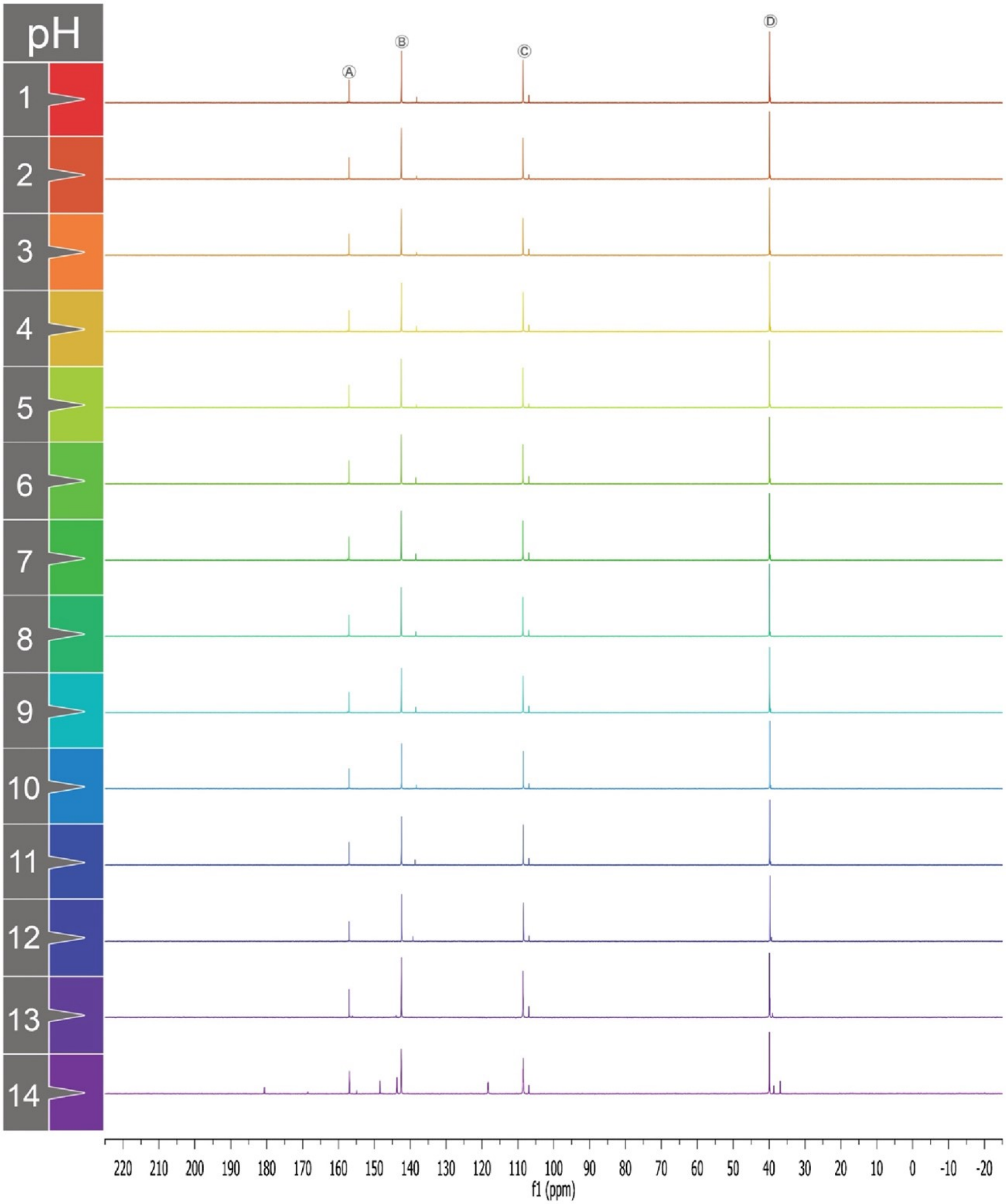

^{10}B-NMR, T = 4 months

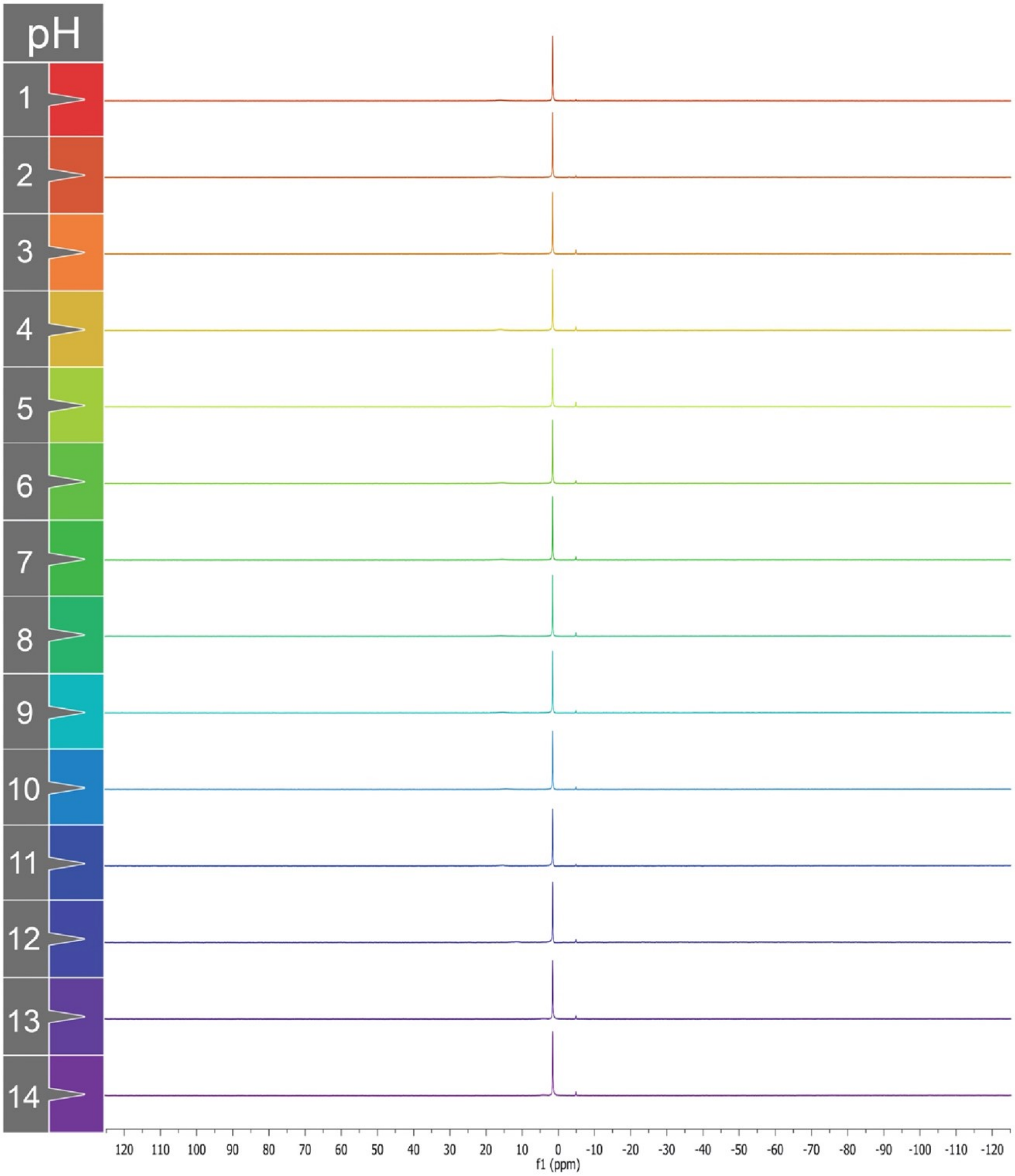

^{10}B-NMR Shifts over time, compound 1 summary:

pH	^{10}B Chemical Shift		
	t=0	t=1 week	t=4 mon
1	1.440	1.436	1.440
2	1.460	1.467	1.462
3	1.464	1.469	1.460
4	1.451	1.453	1.462
5	1.470	1.477	1.478
6	1.468	1.472	1.460
7	1.470	1.470	1.469
8	1.467	1.475	1.475
9	1.477	1.458	1.450
10	1.443	1.443	1.442
11	1.461	1.463	1.450
12	1.435	1.441	1.428
13	1.453	1.453	1.447
14	1.451	1.455	1.425
**AVERAGE**	1.46 ± 0.01	1.46 ± 0.01	1.45 ± 0.02

NMRs (pH stability study, compound $2Br_3$) Labelling keys

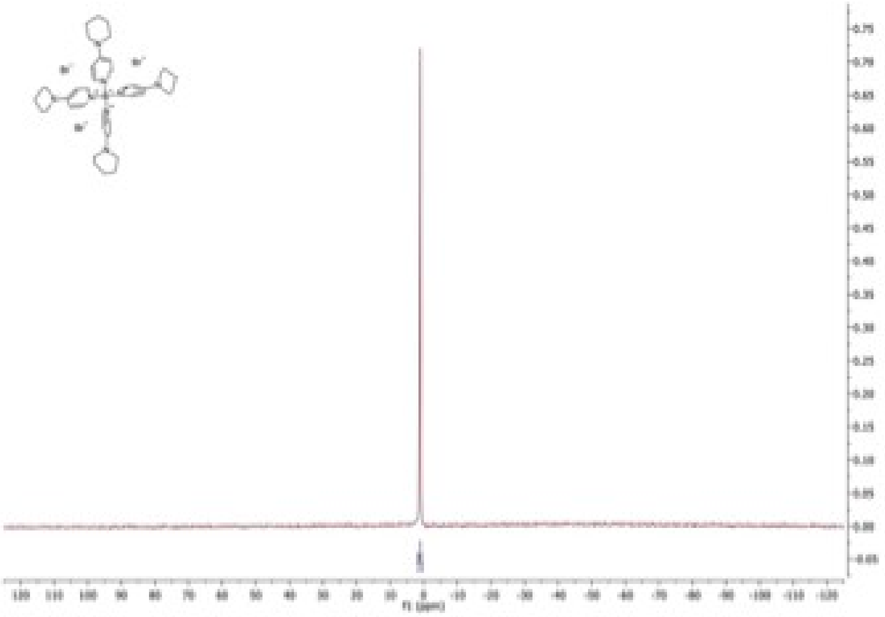

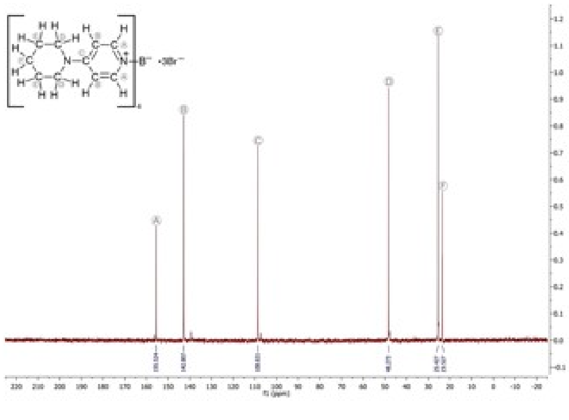

•3Br⁻
F1 (ppm)

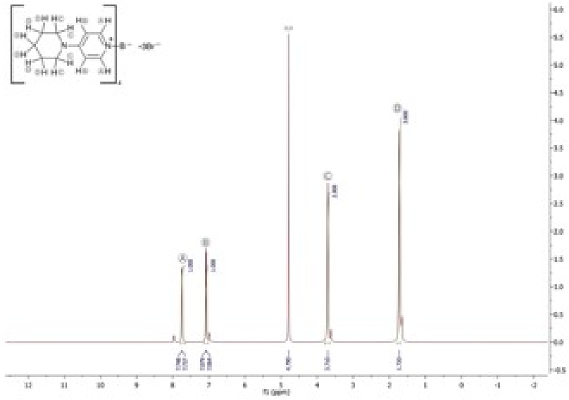

·3Br⁻
f1 (ppm)

^{1}H-NMR T = 5 min

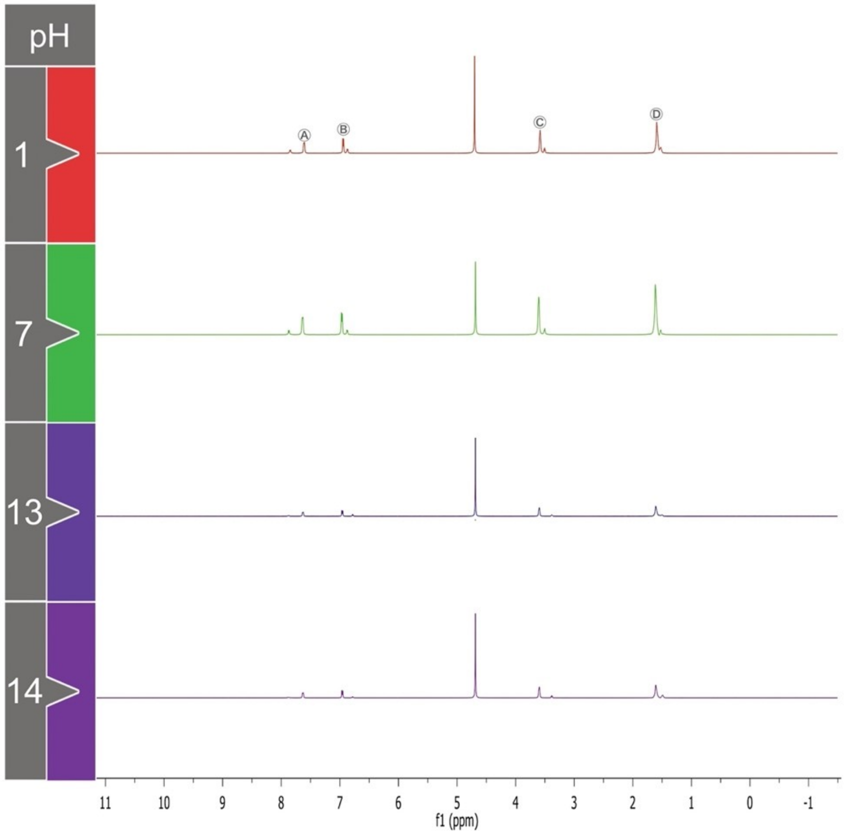

^{1}H-NMR T=1 wk

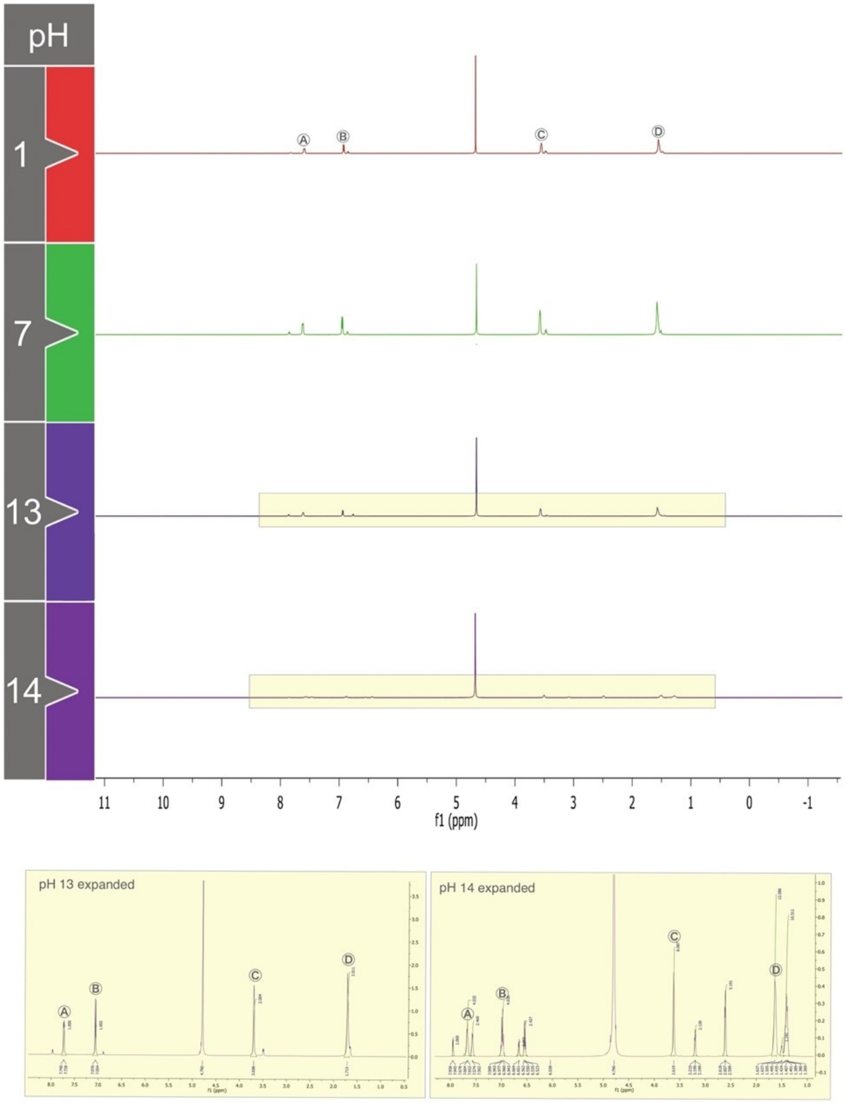
pH
1
7
13
14
A
B
C
D
11 10 9 8 7 6 5 4 3 2 1 0 -1
f1 (ppm)
pH 13 expanded
pH 14 expanded

^{1}H-NMR T = 4 mos

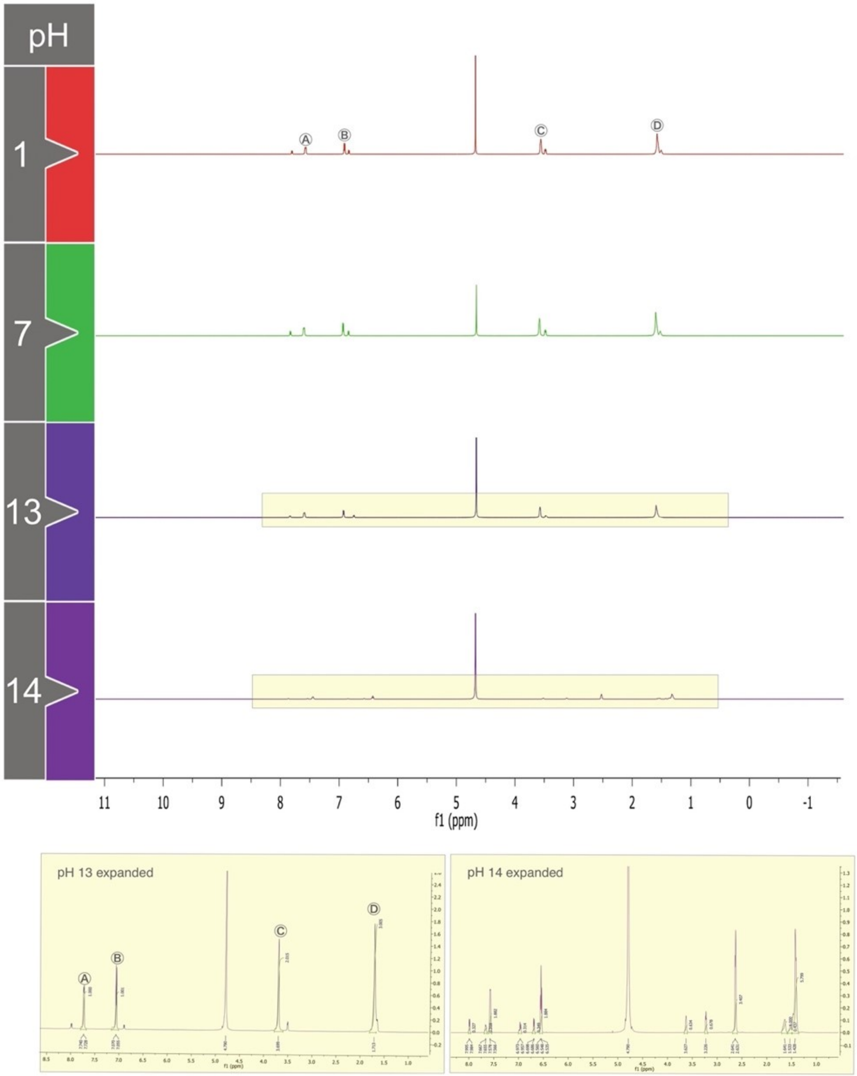
pH
1
7
13
14
A
B
C
D
11
10
9
8
7
6
5
4
3
2
1
0
-1
f1 (ppm)
pH 13 expanded
pH 14 expanded

^{13}C-NMR T = 5 min

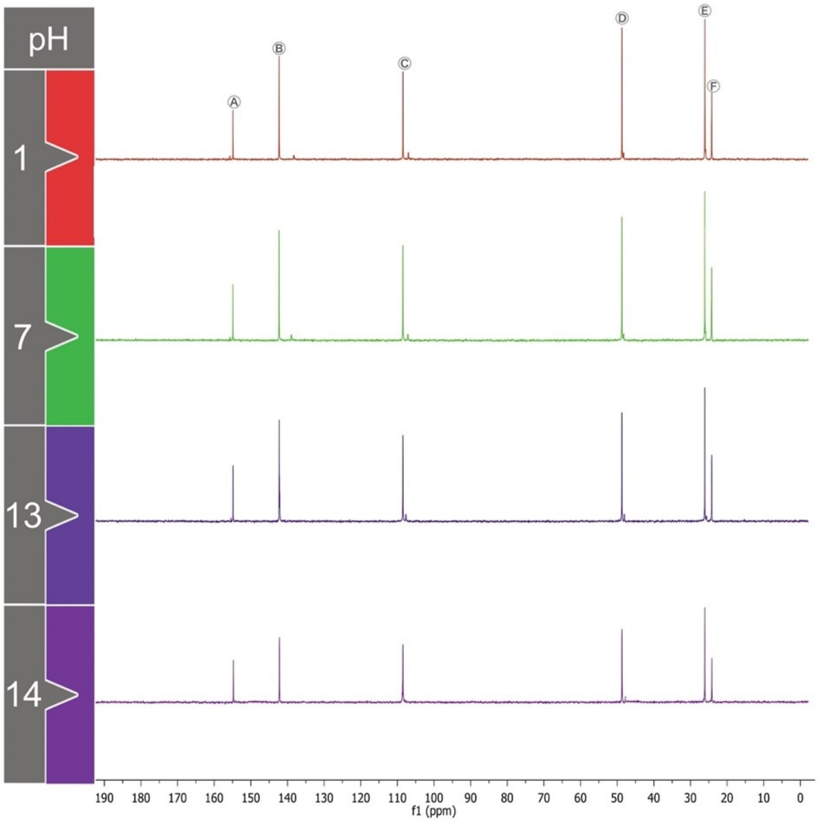

^{13}C-NMR T = 1 wk

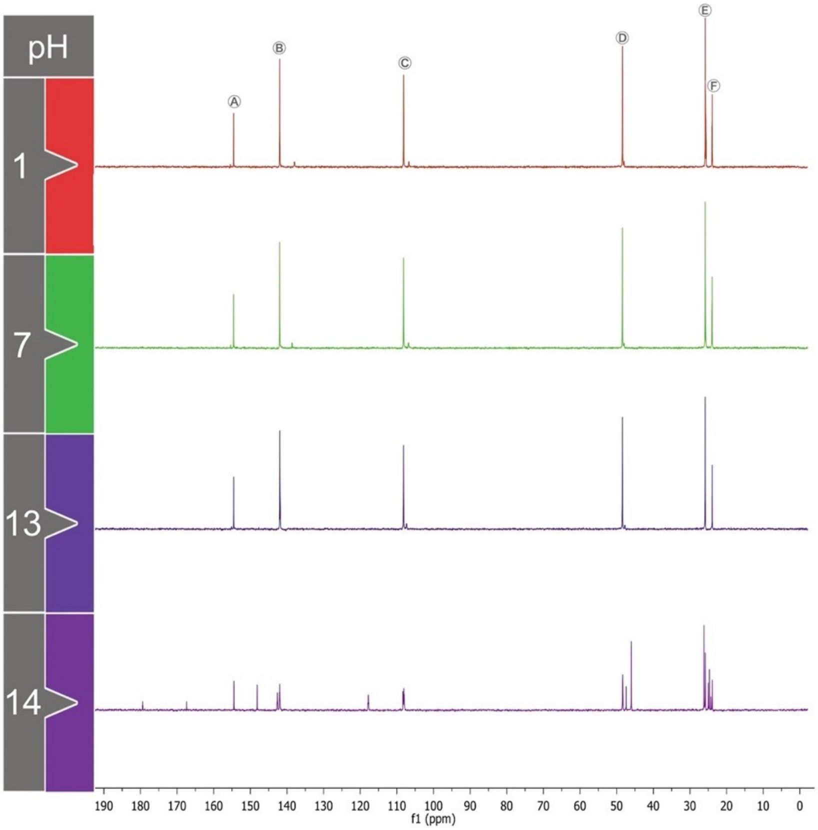
pH
1
7
13
14
A
B
C
D
E
F
190 180 170 160 150 140 130 120 110 100 90 80 70 60 50 40 30 20 10 0
f1 (ppm)

^{13}C-NMR T = 4 mos

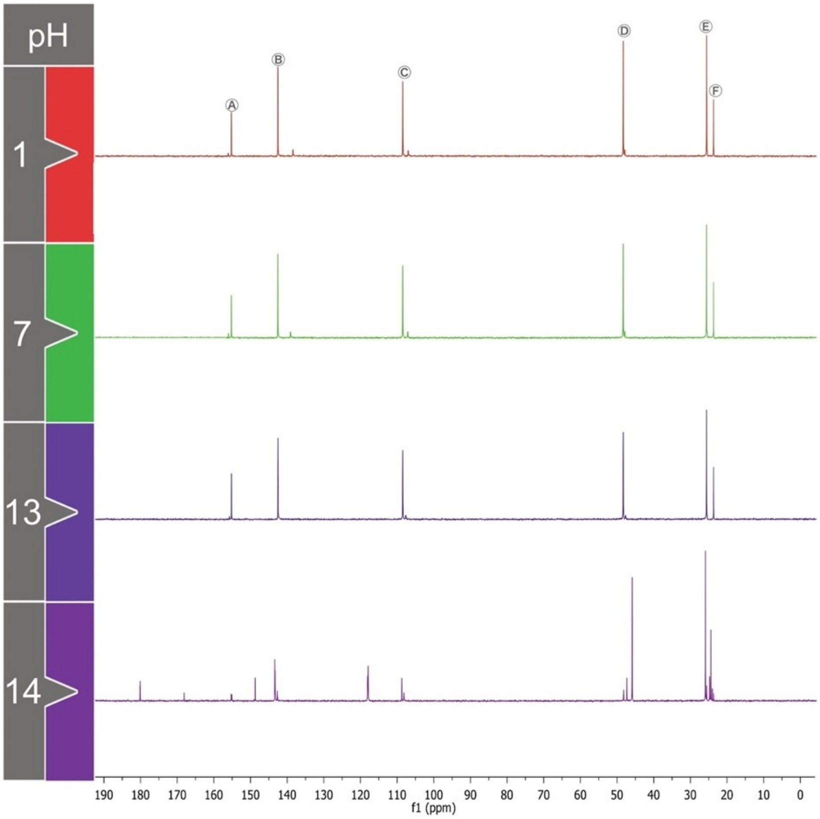

^{10}**B-NMR T = 5 min**

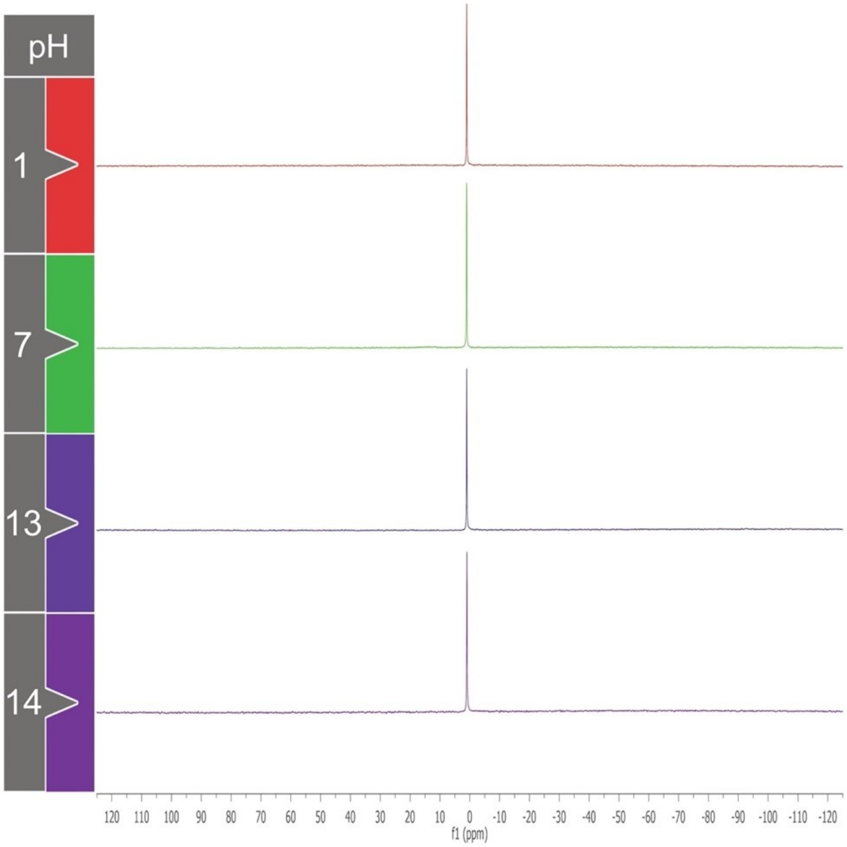

^{10}B-NMR T = 1 wk

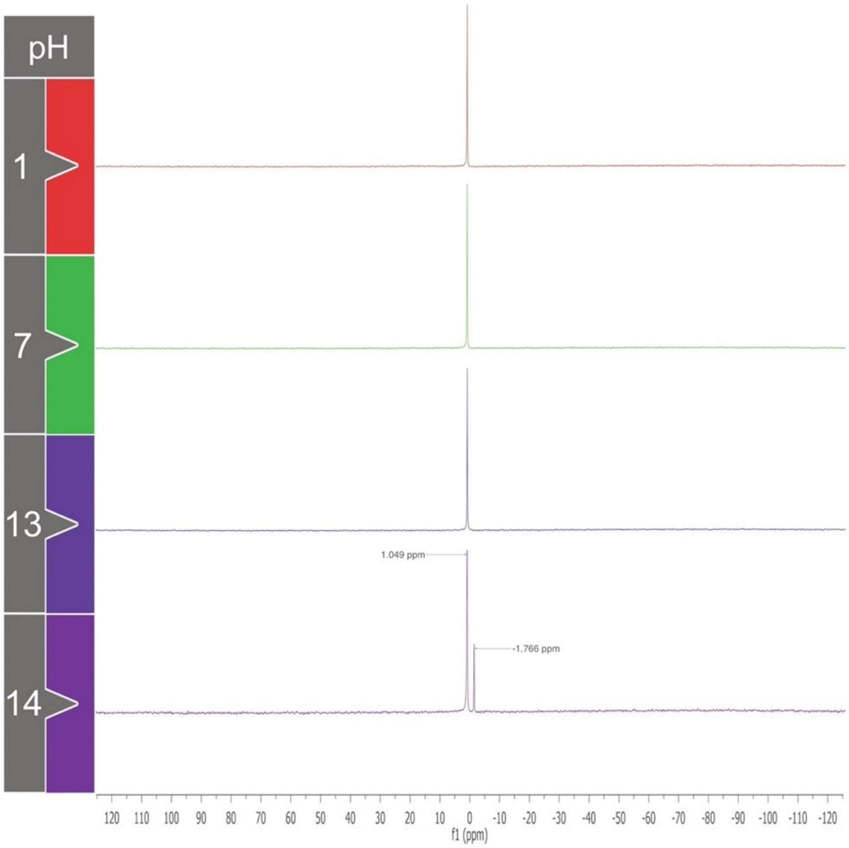

pH
1
7
13
14
1.049 ppm
-1.766 ppm
120 110 100 90 80 70 60 50 40 30 20 10 0 -10 -20 -30 -40 -50 -60 -70 -80 -90 -100 -110 -120
f1 (ppm)

^{10}B-NMR T = 4 mos

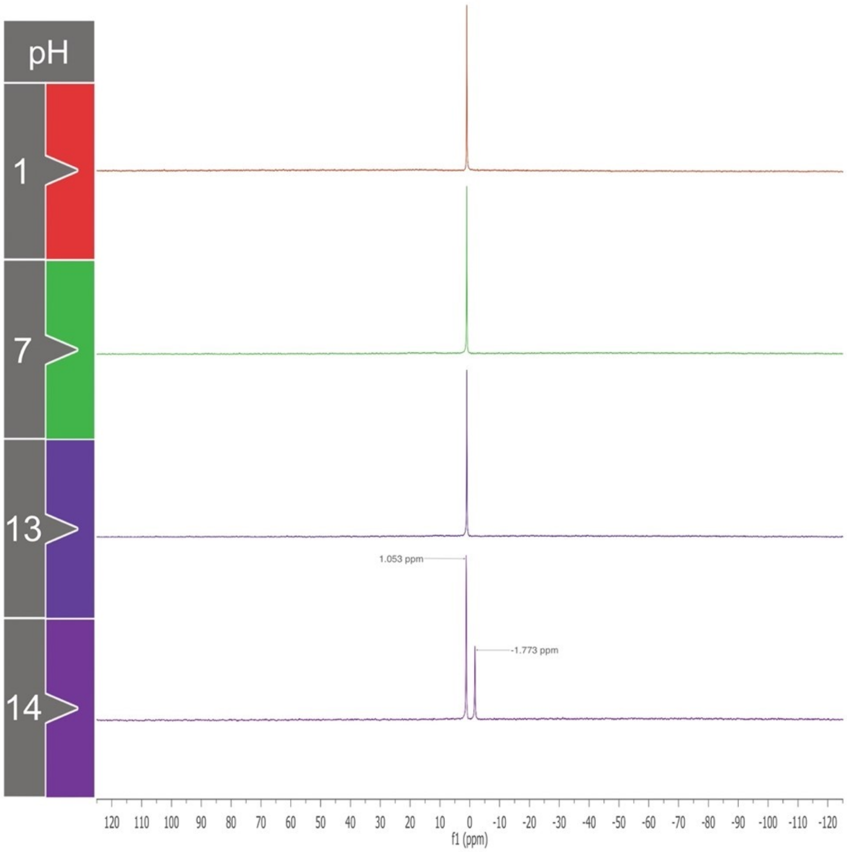

X-ray crystallographic information

Deposition Number 2394932-2394936

Summary of Data - Deposition Number 2394932

Compound Name: **2**$(Tf_2N)_3$
Data Block Name: data_GM1_56_0m
Unit Cell Parameters: a 17.2248(8) b 13.2729(6) c 27.7992(10) P21/n
----

Summary of Data - Deposition Number 2394933

Compound Name: **1**$(Tf_2N)_3$
Data Block Name: data_JD_08_16_23_A_0m
Unit Cell Parameters: a 11.1886(4) b 12.3411(5) c 20.7080(9) P-1

Summary of Data - Deposition Number 2394934

Compound Name: **3**$(Tf_2N)_3$
Data Block Name: data_mc2_81_b_0m_sq
Unit Cell Parameters: a 12.8183(4) b 15.0863(4) c 18.2974(6) P-1

Summary of Data - Deposition Number 2394936

Compound Name: **5**$(Tf_2N)_3$
Data Block Name: data_JD_09_28_23_0m
Unit Cell Parameters: a 13.3318(5) b 29.6715(11) c 17.2747(7) Cc

checkCIF (basic structural check) running

Checking for embedded fcf data in CIF ...
Found embedded fcf data in CIF. Extracting fcf data from uploaded CIF, please wait . . . .

checkCIF/PLATON (basic structural check)

Structure factors have been supplied for datablock(s) GM1_56_0m

THIS REPORT IS FOR GUIDANCE ONLY. IF USED AS PART OF A REVIEW PROCEDURE FOR PUBLICATION, IT SHOULD NOT REPLACE THE EXPERTISE OF AN EXPERIENCED CRYSTALLOGRAPHIC REFEREE.

No syntax errors found. CIF dictionary
Please wait while processing .... Interpreting this report

Structure factor report

Datablock: GM1_56_0m

```
Bond precision:          C-C = 0.0025 A                Wavelength=0.71073
Cell:        a=17.2248(8)       b=13.2729(6)       c=27.7992(10)
             alpha=90           beta=90.267(2)     gamma=90
Temperature:150 K
```

	Calculated	Reported
Volume	6355.5(5)	6355.5(5)
Space group	P 21/n	P 21/n
Hall group	-P 2yn	-P 2yn
Moiety formula	C40 H56 B N8, 3(C2 F6 N O4 S2), 0.29(H2 O)	C40 H56 B N8, 3(C2 F6 N O4 S2), 0.29(H2 O)
Sum formula	C46 H56.58 B F18 N11 O12.29 S6	C46 H56.58 B F18 N11 O12.29 S6
Mr	1505.38	1505.38
Dx,g cm-3	1.573	1.573
Z	4	4
Mu (mm-1)	0.334	0.334
F000	3083.5	3083.5
F000'	3088.69	
h,k,lmax	26,20,42	26,20,42
Nref	24322	24204
Tmin,Tmax	0.905,0.961	0.682,0.747
Tmin'	0.872	

```
Correction method= # Reported T Limits: Tmin=0.682 Tmax=0.747 AbsCorr = MULTI-SCAN
Data completeness= 0.995            Theta(max)= 33.175
R(reflections)= 0.0503( 19244)      wR2(reflections)= 0.1461( 24204)
S = 1.052                Npar= 1278
```

The following ALERTS were generated. Each ALERT has the format **test-name_ALERT_alert-type_alert-level**.
Click on the hyperlinks for more details of the test.

Alert level C

PLAT410_ALERT_2_C Short Intra H...H Contact H2_1 ..H6A_1 . 1.93 Ang.
x,y,z = 1_555 Check
And 7 other PLAT410 Alerts
More ...
PLAT910_ALERT_3_C Missing # of FCF Reflection(s) Below Theta(Min). 7 Note PLAT911_ALERT_3_C Missing FCF Refl Between Thmin & STh/L= 0.600 43 Report PLAT913_ALERT_3_C Missing # of Very Strong Reflections in FCF .... 16 Note

Alert level G

PLAT002_ALERT_2_G Number of Distance or Angle Restraints on AtSite 96 Note
PLAT003_ALERT_2_G Number of Uiso or Uij Restrained non-H Atoms ... 100 Report
PLAT172_ALERT_4_G The CIF-Embedded .res File Contains DFIX Records 4 Report
PLAT175_ALERT_4_G The CIF-Embedded .res File Contains SAME Records 5 Report
PLAT178_ALERT_4_G The CIF-Embedded .res File Contains SIMU Records 2 Report
PLAT230_ALERT_2_G Hirshfeld Test Diff for S2_2 --O3_2 . 6.5 s.u.
PLAT230_ALERT_2_G Hirshfeld Test Diff for F6_3 --C12_3 . 6.2 s.u.
PLAT242_ALERT_2_G Low 'MainMol' Ueq as Compared to Neighbors of C11_1 Check
PLAT242_ALERT_2_G Low 'MainMol' Ueq as Compared to Neighbors of C12_1 Check
PLAT302_ALERT_4_G Anion/Solvent/Minor-Residue Disorder (Resd 3) 100% Note
And 5 other PLAT302 Alerts
More ...
PLAT304_ALERT_4_G Non-Integer Number of Atoms in ..... (Resd 3) 7.76 Check
And 5 other PLAT304 Alerts
More ...
PLAT432_ALERT_2_G Short Inter X...Y Contact O4_2 ..C1_3 . 3.01 Ang.
1/2-x,-1/2+y,1/2-z = 2_545 Check
PLAT432_ALERT_2_G Short Inter X...Y Contact F4C_2 ..C6_2 . 2.70 Ang.
1/2-x,-1/2+y,1/2-z = 2_545 Check
And 3 other PLAT432 Alerts
More ...
1-x,-y,1-z = 3_656 Check
PLAT434_ALERT_2_G Short Inter HL..HL Contact F1_1 ..F1_1 . 2.63 Ang.
1-x,1-y,-z = 3_665 Check
PLAT720_ALERT_4_G Number of Unusual/Non-Standard Labels .... 197 Note
PLAT790_ALERT_4_G Centre of Gravity not Within Unit Cell: Resd. # 4 Note
C2 F6 N O4 S2
PLAT790_ALERT_4_G Centre of Gravity not Within Unit Cell: Resd. # 7 Note
C2 F6 N O4 S2
PLAT811_ALERT_5_G No ADDSYM Analysis: Too Many Excluded Atoms .... ! Info
PLAT860_ALERT_3_G Number of Least-Squares Restraints .... 2338 Note
PLAT912_ALERT_4_G Missing # of FCF Reflections Above STh/L= 0.600 67 Note
PLAT933_ALERT_2_G Number of HKL-OMIT Records in Embedded .res File 4 Note
PLAT941_ALERT_3_G Average HKL Measurement Multiplicity .... 4.9 Low
PLAT978_ALERT_2_G Number C-C Bonds with Positive Residual Density. 4 Info
PLAT992_ALERT_5_G Repd & Actual _reflns_number_gt Values Differ by 2 Check

0 **ALERT level A** = Most likely a serious problem - resolve or explain
0 **ALERT level B** = A potentially serious problem, consider carefully
11 **ALERT level C** = Check. Ensure it is not caused by an omission or oversight
37 **ALERT level G** = General information/check it is not something unexpected

0 ALERT type 1 CIF construction/syntax error, inconsistent or missing data
22 ALERT type 2 Indicator that the structure model may be wrong or deficient
5 ALERT type 3 Indicator that the structure quality may be low
19 ALERT type 4 Improvement, methodology, query or suggestion
2 ALERT type 5 Informative message, check

It is advisable to attempt to resolve as many as possible of the alerts in all categories. Often the minor alerts point to easily fixed oversights, errors and omissions in your CIF or refinement strategy, so attention to these fine details can be worthwhile. In order to resolve some of the more serious problems it may be necessary to carry out additional measurements or structure refinements. However, the purpose of your study may justify the reported deviations and the more serious of these should normally be commented upon in the discussion or experimental section of a paper or in the "special_details" fields of the CIF.

checkCIF was carefully designed to identify outliers and unusual parameters, but every test has its limitations and alerts that are not important in a particular case may appear. Conversely, the absence of alerts does not guarantee there are no aspects of the results needing attention. It is up to the individual to critically assess their own results and, if necessary, seek expert advice.

Publication of your CIF in IUCr journals

A basic structural check has been run on your CIF. These basic checks will be run on all CIFs submitted for publication in IUCr journals (*Acta Crystallographica*, *Journal of Applied Crystallography*, *Journal of Synchrotron Radiation*); however, if you intend to submit to *Acta Crystallographica Section C* or *E* or *IUCrData*, you should make sure that full publication checks are run on the final version of your CIF prior to submission.

Publication of your CIF in other journals

Please refer to the *Notes for Authors* of the relevant journal for any special instructions relating to CIF submission.

PLATON version of 06/07/2023; check.def file version of 30/06/2023

Datablock GM1_56_0m - ellipsoid plot

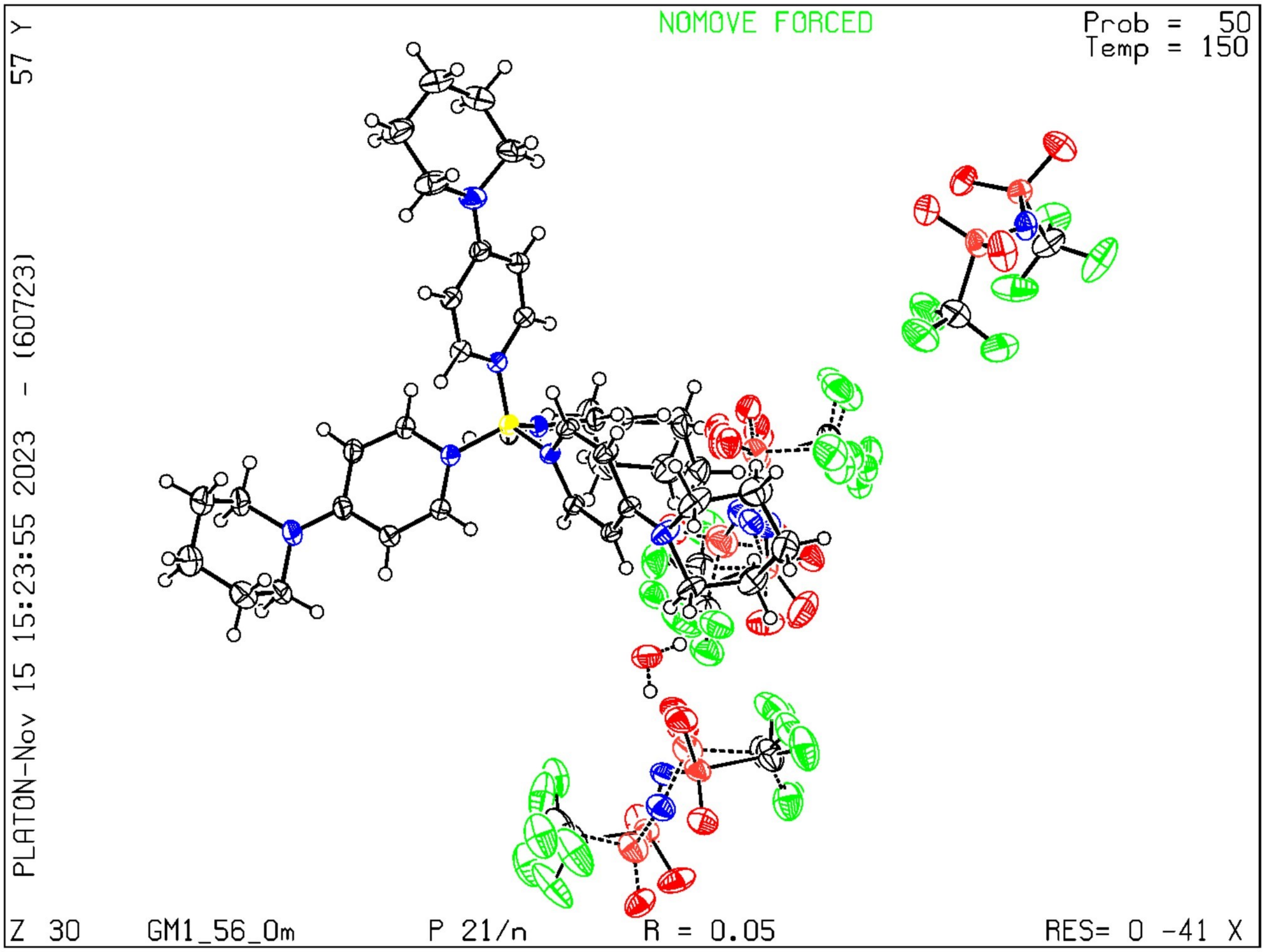

Download CIF editor (publCIF) from the IUCr
Download CIF editor (enCIFer) from the CCDC
Test a new CIF entry

checkCIF (basic structural check) running

Checking for embedded fcf data in CIF ...
Found embedded fcf data in CIF. Extracting fcf data from uploaded CIF, please wait . . . . .

checkCIF/PLATON (basic structural check)

Structure factors have been supplied for datablock(s) JD_08_16_23_A_0m

THIS REPORT IS FOR GUIDANCE ONLY. IF USED AS PART OF A REVIEW PROCEDURE FOR PUBLICATION, IT SHOULD NOT REPLACE THE EXPERTISE OF AN EXPERIENCED CRYSTALLOGRAPHIC REFEREE.

No syntax errors found. CIF dictionary
Please wait while processing .... Interpreting this report

Structure factor report

Datablock: JD_08_16_23_A_0m

Bond precision:	C-C = 0.0020 A		Wavelength=0.71073
Cell:	a=11.1886(4)	b=12.3411(5)	c=20.7080(9)
	alpha=104.968(2)	beta=96.406(2)	gamma=100.585(2)
Temperature:150 K			

	Calculated	Reported
Volume	2676.99(19)	2676.99(19)
Space group	P -1	P -1
Hall group	-P 1	-P 1
Moiety formula	C28 H40 B N8, 3(C2 F6 N O4 S2)	C28 H40 B N8, 3(C2 F6 N O4 S2)
Sum formula	C34 H40 B F18 N11 O12 S6	C34 H40 B F18 N11 O12 S6
Mr	1339.94	1339.94
Dx,g cm-3	1.662	1.662
Z	2	2
Mu (mm-1)	0.385	0.385
F000	1360.0	1360.0
F000'	1362.51 h,k,lmax 17,19,31	17,18,31
Nref	20459	19242
Tmin,Tmax	0.908,0.985	0.655,0.747 Tmin' 0.887

Correction method= # Reported T Limits: Tmin=0.655 Tmax=0.747 AbsCorr = MULTI-SCAN

Data completeness= 0.941 Theta(max)= 33.175

R(reflections)= 0.0443(11708) wR2(reflections)= 0.1218(19242)

S = 1.036 Npar= 1009

The following ALERTS were generated. Each ALERT has the format **test-name_ALERT_alert-type_alert-level**.
Click on the hyperlinks for more details of the test.

Alert level C

PLAT230_ALERT_2_C Hirshfeld Test Diff for S2_2 --C2_2 . 5.5 s.u.
PLAT906_ALERT_3_C Large K Value in the Analysis of Variance ...... 14.564 Check
PLAT906_ALERT_3_C Large K Value in the Analysis of Variance ...... 2.147 Check
PLAT910_ALERT_3_C Missing # of FCF Reflection(s) Below Theta(Min). 5 Note
PLAT911_ALERT_3_C Missing FCF Refl Between Thmin & STh/L= 0.600 3 Report

Alert level G

PLAT002_ALERT_2_G Number of Distance or Angle Restraints on AtSite 75 Note
PLAT003_ALERT_2_G Number of Uiso or Uij Restrained non-H Atoms ... 60 Report
PLAT154_ALERT_1_G The s.u.'s on the Cell Angles are Equal ..(Note) 0.002 Degree
PLAT168_ALERT_4_G The CIF-Embedded .res File Contains EXYZ Records 1 Report
PLAT171_ALERT_4_G The CIF-Embedded .res File Contains EADP Records 1 Report
PLAT175_ALERT_4_G The CIF-Embedded .res File Contains SAME Records 4 Report
PLAT178_ALERT_4_G The CIF-Embedded .res File Contains SIMU Records 3 Report
PLAT242_ALERT_2_G Low 'MainMol' Ueq as Compared to Neighbors of C1_2 Check
PLAT242_ALERT_2_G Low 'MainMol' Ueq as Compared to Neighbors of C2_2
Check PLAT300_ALERT_4_G Atom Site Occupancy of S1_4 Constrained at 0.5
Check **And 29 other PLAT300 Alerts** More ...
PLAT302_ALERT_4_G Anion/Solvent/Minor-Residue Disorder (Resd 3) 100% Note
And 3 other PLAT302 Alerts More ...
PLAT304_ALERT_4_G Non-Integer Number of Atoms in ..... (Resd 4) 7.50 Check
PLAT304_ALERT_4_G Non-Integer Number of Atoms in ..... (Resd 5) 7.50 Check
PLAT432_ALERT_2_G Short Inter X...Y Contact O1_3 ..C24_1 . 2.94 Ang.
1-x,1-y,2-z = 2_667 Check
PLAT434_ALERT_2_G Short Inter HL..HL Contact F6_2 ..F5_3 . 2.74 Ang.
x,y,z = 1_555 Check
PLAT720_ALERT_4_G Number of Unusual/Non-Standard Labels .... 152 Note
PLAT789_ALERT_4_G Atoms with Negative _atom_site_disorder_group # 30 Check
PLAT811_ALERT_5_G No ADDSYM Analysis: Too Many Excluded Atoms .... ! Info
PLAT822_ALERT_4_G CIF-embedded .res Contains Negative PART Numbers 2 Check
PLAT860_ALERT_3_G Number of Least-Squares Restraints .... 1002 Note
PLAT912_ALERT_4_G Missing # of FCF Reflections Above STh/L= 0.600 1208 Note
PLAT933_ALERT_2_G Number of HKL-OMIT Records in Embedded .res File 1 Note
PLAT941_ALERT_3_G Average HKL Measurement Multiplicity .... 2.9 Low
PLAT978_ALERT_2_G Number C-C Bonds with Positive Residual Density. 8 Info

0 **ALERT level A** = Most likely a serious problem - resolve or explain
0 **ALERT level B** = A potentially serious problem, consider carefully
5 **ALERT level C** = Check. Ensure it is not caused by an omission or oversight
56 **ALERT level G** = General information/check it is not something unexpected

1 ALERT type 1 CIF construction/syntax error, inconsistent or missing data
9 ALERT type 2 Indicator that the structure model may be wrong or deficient
6 ALERT type 3 Indicator that the structure quality may be low
44 ALERT type 4 Improvement, methodology, query or suggestion
1 ALERT type 5 Informative message, check

It is advisable to attempt to resolve as many as possible of the alerts in all categories. Often the minor alerts point to easily fixed oversights, errors and omissions in your CIF or refinement strategy, so attention to these fine details can be worthwhile. In order to resolve some of the more serious problems it may be necessary to carry out additional measurements or structure refinements. However, the purpose of your study may justify the reported deviations and the more serious of these should normally be commented upon in the discussion or experimental section of a paper or in the "special_details" fields of the CIF. checkCIF was carefully designed to identify outliers and unusual parameters, but every test has its limitations and alerts that are not important in a particular case may appear. Conversely, the absence of alerts does not guarantee there are no aspects of the results needing attention. It is up to the individual to critically assess their own results and, if necessary, seek expert advice.

Publication of your CIF in IUCr journals

A basic structural check has been run on your CIF. These basic checks will be run on all CIFs submitted for publication in IUCr journals (*Acta Crystallographica*, *Journal of Applied Crystallography*, *Journal of Synchrotron Radiation*); however, if you intend to submit to *Acta Crystallographica Section C* or *E* or

IUCrData, you should make sure that full publication checks are run on the final version of your CIF prior to submission.

Publication of your CIF in other journals

Please refer to the *Notes for Authors* of the relevant journal for any special instructions relating to CIF submission.

PLATON version of 06/07/2023; check.def file version of 30/06/2023

Datablock JD_08_16_23_A_0m - ellipsoid plot

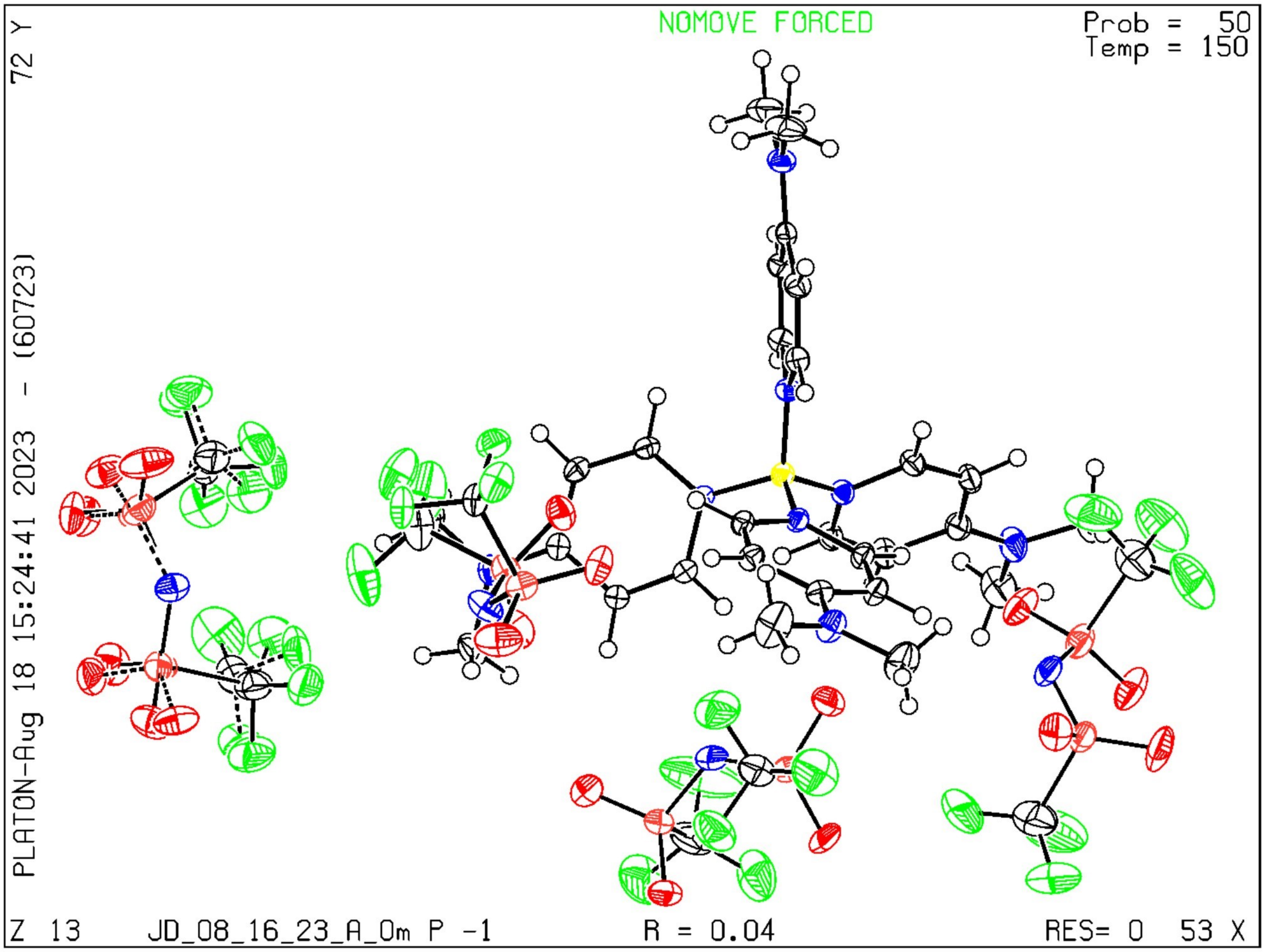

Download CIF editor (publCIF) from the IUCr
Download CIF editor (enCIFer) from the CCDC
Test a new CIF entry

checkCIF (basic structural check) running

Checking for embedded fcf data in CIF ...
Found embedded fcf data in CIF. Extracting fcf data from uploaded CIF, please wait . . . .

checkCIF/PLATON (basic structural check)

Structure factors have been supplied for datablock(s) JD_09_28_23_0m

THIS REPORT IS FOR GUIDANCE ONLY. IF USED AS PART OF A REVIEW PROCEDURE FOR PUBLICATION, IT SHOULD NOT REPLACE THE EXPERTISE OF AN EXPERIENCED CRYSTALLOGRAPHIC REFEREE.

No syntax errors found. CIF dictionary
Please wait while processing .... Interpreting this report

Structure factor report

Datablock: JD_09_28_23_0m

Bond precision: C-C = 0.0029 A Wavelength=0.71073

Cell: a=13.3318(5) b=29.6715(11) c=17.2747(7) alpha=90
beta=110.925(1) gamma=90

Temperature:150 K

	Calculated	Reported
Volume	6382.8(4)	6382.8(4)
Space group	C c	C c
Hall group	C -2yc	C -2yc
Moiety formula	C44 H56 B N8, 3(C2 F6 N O4 S2)	C44 H56 B N8, 3(C2 F6 N O4 S2)
Sum formula	C50 H56 B F18 N11 O12 S6	C50 H56 B F18 N11 O12 S6
Mr	1547.95	1548.22
Dx,g cm-3	1.611	1.611
Z	4	4
Mu (mm-1)	0.335	0.335
F000	3167.5	3168.0

F000' 3172.62 h,k,lmax 20,45,26 20,45,26 Nref 24402[12212] 23201

Tmin,Tmax 0.843,0.883 0.696,0.747 Tmin' 0.843

Correction method= # Reported T Limits: Tmin=0.696 Tmax=0.747 AbsCorr = MULTI-SCAN

Data completeness= 1.90/0.95 Theta(max)= 33.186

R(reflections)= 0.0328(21354) wR2(reflections)= 0.0896(23201)

S = 1.018 Npar= 1273

The following ALERTS were generated. Each ALERT has the format **test-name_ALERT_alert-type_alert-level**.
Click on the hyperlinks for more details of the test.

Alert level G

CELLZ01_ALERT_1_G Difference between formula and atom_site contents detected.
CELLZ01_ALERT_1_G ALERT: check formula stoichiometry or atom site occupancies.
From the CIF: _cell_formula_units_Z 4

From the CIF: _chemical_formula_sum C50 H56 B F18 N11 O12 S6
TEST: Compare cell contents of formula and atom_site data

atom	Z*formula	cif sites	diff
C	200.00	199.99	0.01
H	224.00	224.00	0.00
B	4.00	4.00	0.00
F	72.00	71.98	0.02
N	44.00	44.00	0.00
O	48.00	47.98	0.02
S	24.00	23.99	0.01

PLAT002_ALERT_2_G Number of Distance or Angle Restraints on AtSite 97 Note
PLAT003_ALERT_2_G Number of Uiso or Uij Restrained non-H Atoms ... 73 Report
PLAT068_ALERT_1_G Reported F000 Differs from Calcd (or Missing)... Please Check
PLAT171_ALERT_4_G The CIF-Embedded .res File Contains EADP Records 2 Report
PLAT174_ALERT_4_G The CIF-Embedded .res File Contains FLAT Records 1 Report
PLAT175_ALERT_4_G The CIF-Embedded .res File Contains SAME Records 9 Report
PLAT176_ALERT_4_G The CIF-Embedded .res File Contains SADI Records 2 Report
PLAT178_ALERT_4_G The CIF-Embedded .res File Contains SIMU Records 4 Report
PLAT230_ALERT_2_G Hirshfeld Test Diff for S2_7 --N1_7 . 8.6 s.u.
PLAT242_ALERT_2_G Low 'MainMol' Ueq as Compared to Neighbors of C2_5 Check

And 2 other PLAT242 Alerts

More ...

PLAT301_ALERT_3_G Main Residue Disorder ....(Resd 1) 11% Note
PLAT302_ALERT_4_G Anion/Solvent/Minor-Residue Disorder (Resd 2) 53% Note

And 3 other PLAT302 Alerts

More ...

PLAT304_ALERT_4_G Non-Integer Number of Atoms in ..... (Resd 4) 9.15 Check

And 2 other PLAT304 Alerts

More ...

PLAT411_ALERT_2_G Short Inter H...H Contact H8B_1 ..H8C_3 . 2.05 Ang.
x,-y,1/2+z = 2_555 Check
PLAT411_ALERT_2_G Short Inter H...H Contact H7B_2 ..H7C_3 . 2.10 Ang.
1/2+x,1/2-y,1/2+z = 4_555 Check
PLAT432_ALERT_2_G Short Inter X...Y Contact O2B_5 ..C1_3 . 2.97 Ang.
x,y,z = 1_555 Check
PLAT434_ALERT_2_G Short Inter HL..HL Contact F1_6 ..F5_7 . 2.79 Ang.
x,y,z = 1_555 Check

And 4 other PLAT434 Alerts

More ...

PLAT720_ALERT_4_G Number of Unusual/Non-Standard Labels .... 209 Note
PLAT790_ALERT_4_G Centre of Gravity not Within Unit Cell: Resd. # 3 Note
C2 F6 N O4 S2
PLAT811_ALERT_5_G No ADDSYM Analysis: Too Many Excluded Atoms .... ! Info
PLAT860_ALERT_3_G Number of Least-Squares Restraints .... 1963 Note
PLAT910_ALERT_3_G Missing # of FCF Reflection(s) Below Theta(Min). 4 Note
PLAT912_ALERT_4_G Missing # of FCF Reflections Above STh/L= 0.600 9 Note
PLAT933_ALERT_2_G Number of HKL-OMIT Records in Embedded .res File 1 Note
PLAT978_ALERT_2_G Number C-C Bonds with Positive Residual Density. 8 Info

0 **ALERT level A** = Most likely a serious problem - resolve or explain
0 **ALERT level B** = A potentially serious problem, consider carefully
0 **ALERT level C** = Check. Ensure it is not caused by an omission or oversight
38 **ALERT level G** = General information/check it is not something unexpected

3 ALERT type 1 CIF construction/syntax error, inconsistent or missing data
16 ALERT type 2 Indicator that the structure model may be wrong or deficient
3 ALERT type 3 Indicator that the structure quality may be low
15 ALERT type 4 Improvement, methodology, query or suggestion
1 ALERT type 5 Informative message, check

It is advisable to attempt to resolve as many as possible of the alerts in all categories. Often the minor alerts point to easily fixed oversights, errors and omissions in your CIF or refinement strategy, so attention to these fine details can be worthwhile. In order to resolve some of the more serious problems it may be

necessary to carry out additional measurements or structure refinements. However, the purpose of your study may justify the reported deviations and the more serious of these should normally be commented upon in the discussion or experimental section of a paper or in the "special_details" fields of the CIF. checkCIF was carefully designed to identify outliers and unusual parameters, but every test has its limitations and alerts that are not important in a particular case may appear. Conversely, the absence of alerts does not guarantee there are no aspects of the results needing attention. It is up to the individual to critically assess their own results and, if necessary, seek expert advice.

Publication of your CIF in IUCr journals

A basic structural check has been run on your CIF. These basic checks will be run on all CIFs submitted for publication in IUCr journals (*Acta Crystallographica*, *Journal of Applied Crystallography*, *Journal of Synchrotron Radiation*); however, if you intend to submit to *Acta Crystallographica Section C* or *E* or *IUCrData*, you should make sure that full publication checks are run on the final version of your CIF prior to submission.

Publication of your CIF in other journals

Please refer to the *Notes for Authors* of the relevant journal for any special instructions relating to CIF submission.

PLATON version of 06/07/2023; check.def file version of 30/06/2023

Datablock JD_09_28_23_0m - ellipsoid plot

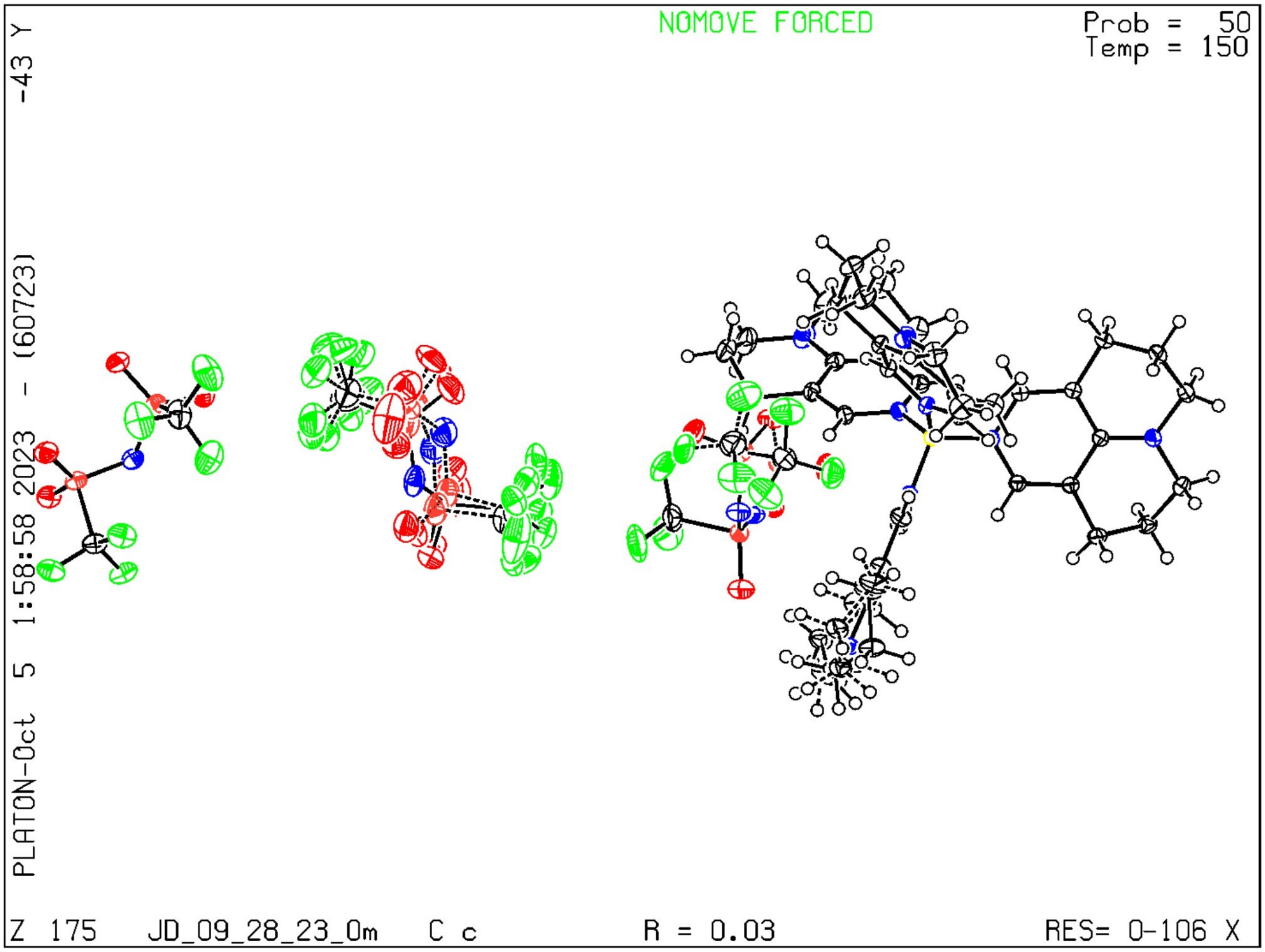

Download CIF editor (publCIF) from the IUCr
Download CIF editor (enCIFer) from the CCDC
Test a new CIF entry

checkCIF (basic structural check) running

Checking for embedded fcf data in CIF ...
Found embedded fcf data in CIF. Extracting fcf data from uploaded CIF, please wait . . . . .

checkCIF/PLATON (basic structural check)

Structure factors have been supplied for datablock(s) mc2_81_b_0m_sq

THIS REPORT IS FOR GUIDANCE ONLY. IF USED AS PART OF A REVIEW PROCEDURE FOR PUBLICATION, IT SHOULD NOT REPLACE THE EXPERTISE OF AN EXPERIENCED CRYSTALLOGRAPHIC REFEREE.

No syntax errors found. CIF dictionary
Please wait while processing .... Interpreting this report

Structure factor report

Datablock: mc2_81_b_0m_sq

Bond precision:	C-C = 0.0055 A		Wavelength=1.54178
Cell:	a=12.8183(4)	b=15.0863(4)	c=18.2974(6)
	alpha=108.533(2)	beta=91.680(2)	gamma=105.476(2)
Temperature:150 K			

	Calculated	Reported
Volume	3207.91(18)	3207.91(17)
Space group	P -1	P -1
Hall group	-P 1	-P 1
Moiety formula	C36 H48 B N8, 3(C2 F6 N O4 S2) [+ solvent]	C36 H48 B N8, 3(C2 F6 N O4 S2), [+ solvent]
Sum formula	C42 H48 B F18 N11 O12 S6 [+ solvent]	C42 H48 B F18 N11 O12 S6
Mr	1444.17	1444.08
Dx,g cm-3	1.495	1.495
Z	2	2
Mu (mm-1)	3.004	3.004
F000	1472.1	1472.0
F000'	1481.90	
h,k,lmax		16,19,23
Nref		13425

Tmin,Tmax 0.759,0.786 0.614,0.754 Tmin' 0.625

Correction method= # Reported T Limits: Tmin=0.614 Tmax=0.754 AbsCorr = MULTI-SCAN

Data completeness= Theta(max)= 80.491

R(reflections)= 0.0738(8269) wR2(reflections)= 0.2299(13425)

S = 1.046 Npar= 1344

The following ALERTS were generated. Each ALERT has the format **test-name_ALERT_alert-type_alert-level**.
Click on the hyperlinks for more details of the test.

Alert level C

PLAT088_ALERT_3_C Poor Data / Parameter Ratio .... 9.99 Note
PLAT241_ALERT_2_C High 'MainMol' Ueq as Compared to Neighbors of C7_3 Check
PLAT241_ALERT_2_C High 'MainMol' Ueq as Compared to Neighbors of C8_4 Check
PLAT250_ALERT_2_C Large U3/U1 Ratio for Average U(i,j) Tensor .... 2.4 Note
PLAT260_ALERT_2_C Large Average Ueq of Residue Including S1_7 0.107 Check
PLAT260_ALERT_2_C Large Average Ueq of Residue Including S1_8 0.129 Check
PLAT340_ALERT_3_C Low Bond Precision on C-C Bonds .... 0.0055 Ang.
PLAT906_ALERT_3_C Large K Value in the Analysis of Variance ...... 2.195 Check
PLAT911_ALERT_3_C Missing FCF Refl Between Thmin & STh/L= 0.600 15 Report
0 1 4, -15 4 5, 4-17 7, -1 1 7, -12 4 12, 0-16 15,
-5-13 15, -2 3 15, -3 7 16, -9 3 17, -1 5 17, -3 6 17,
-8 2 18, -4 5 18, -3 5 18,

Alert level G

PLAT002_ALERT_2_G Number of Distance or Angle Restraints on AtSite 105 Note
PLAT003_ALERT_2_G Number of Uiso or Uij Restrained non-H Atoms ... 105 Report
PLAT042_ALERT_1_G Calc. and Reported MoietyFormula Strings Differ Please Check
Calc: C36 H48 B N8, 3(C2 F6 N O4 S2)
Rep.: C36 H48 B N8, 3(C2 F6 N O4 S2),
PLAT154_ALERT_1_G The s.u.'s on the Cell Angles are Equal ..(Note) 0.002 Degree
PLAT171_ALERT_4_G The CIF-Embedded .res File Contains EADP Records 2 Report
PLAT175_ALERT_4_G The CIF-Embedded .res File Contains SAME Records 6 Report
PLAT178_ALERT_4_G The CIF-Embedded .res File Contains SIMU Records 4 Report
PLAT300_ALERT_4_G Atom Site Occupancy of S1_7 Constrained at 0.5 Check
And 29 other PLAT300 Alerts
More ...
PLAT302_ALERT_4_G Anion/Solvent/Minor-Residue Disorder (Resd 2) 100% Note
And 6 other PLAT302 Alerts
More ...
PLAT304_ALERT_4_G Non-Integer Number of Atoms in ..... (Resd 2) 10.97 Check
And 6 other PLAT304 Alerts
More ...
PLAT432_ALERT_2_G Short Inter X...Y Contact O3_7 ..C4_1 . 2.90 Ang.
1-x,1-y,-z = 2_665 Check
PLAT605_ALERT_4_G Largest Solvent Accessible VOID in the Structure 302 A**3
PLAT720_ALERT_4_G Number of Unusual/Non-Standard Labels .... 197 Note
N1_1 N2_1 C1_1 H1_1 C2_1 H2_1 C3_1 C4_1
H4_1 C5_1 H5_1 C6_1 H6A_1 H6B_1 C7_1 H7A_1
H7B_1 C8_1 H8A_1 H8B_1 C9_1 H9A_1 H9B_1 N1_2
N2_2 C1_2 H1_2 C2_2 H2_2 C3_2 C4_2 H4_2
C5_2 H5_2 C6_2 H6A_2 H6B_2 C7_2 H7A_2 H7B_2
C8_2 H8A_2 H8B_2 C9_2 H9A_2 H9B_2 N1_3 N2_3
C1_3 H1_3 C2_3 H2_3 C3_3 C4_3 H4_3 C5_3
H5_3 C6_3 H6A_3 H6B_3 C7_3 H7A_3 H7B_3 C8_3
H8A_3 H8B_3 C9_3 H9A_3 H9B_3 N1_4 N2_4 C1_4
H1_4 C2_4 H2_4 C3_4 C4_4 H4_4 C5_4 H5_4
C6_4 H6A_4 H6B_4 C7_4 H7A_4 H7B_4 C8_4 H8A_4
H8B_4 C9_4 H9A_4 H9B_4 F1_5 F2_5 F3_5 C1_5
S1_5 O1_5 O2_5 N1_5 S2_5 O3_5 O4_5 C2_5
F4_5 F5_5 F6_5 F1B_5 F2B_5 F3B_5 C1B_5 S1B_5
O1B_5 O2B_5 N1B_5 S2B_5 O3B_5 O4B_5 C2B_5 F4B_5
F5B_5 F6B_5 F1C_5 F2C_5 F3C_5 C1C_5 S1C_5 O1C_5
O2C_5 N1C_5 S2C_5 O3C_5 O4C_5 C2C_5 F4C_5 F5C_5
F6C_5 F1_6 F2_6 F3_6 C1_6 S1_6 O1_6 O2_6
N1_6 S2_6 O3_6 O4_6 C2_6 F4_6 F5_6 F6_6
F1B_6 F2B_6 F3B_6 C1B_6 S1B_6 O1B_6 O2B_6 N1B_6
S2B_6 O3B_6 O4B_6 C2B_6 F4B_6 F5B_6 F6B_6 F1_7
F2_7 F3_7 C1_7 S1_7 O1_7 O2_7 N1_7 S2_7
O3_7 O4_7 C2_7 F4_7 F5_7 F6_7 F1_8 F2_8
F3_8 C1_8 S1_8 O1_8 O2_8 N1_8 S2_8 O3_8
O4_8 C2_8 F4_8 F5_8 F6_8
PLAT789_ALERT_4_G Atoms with Negative _atom_site_disorder_group # 30 Check

PLAT811_ALERT_5_G No ADDSYM Analysis: Too Many Excluded Atoms .... ! Info
PLAT822_ALERT_4_G CIF-embedded .res Contains Negative PART Numbers 2 Check
PLAT860_ALERT_3_G Number of Least-Squares Restraints .... 2506 Note
PLAT869_ALERT_4_G ALERTS Related to the Use of SQUEEZE Suppressed ! Info
PLAT912_ALERT_4_G Missing # of FCF Reflections Above STh/L= 0.600 645 Note
PLAT941_ALERT_3_G Average HKL Measurement Multiplicity .... 3.7 Low
PLAT978_ALERT_2_G Number C-C Bonds with Positive Residual Density. 0 Info

0 **ALERT level A** = Most likely a serious problem - resolve or explain
0 **ALERT level B** = A potentially serious problem, consider carefully
9 **ALERT level C** = Check. Ensure it is not caused by an omission or oversight
62 **ALERT level G** = General information/check it is not something unexpected

2 ALERT type 1 CIF construction/syntax error, inconsistent or missing data
9 ALERT type 2 Indicator that the structure model may be wrong or deficient
6 ALERT type 3 Indicator that the structure quality may be low
53 ALERT type 4 Improvement, methodology, query or suggestion
1 ALERT type 5 Informative message, check

It is advisable to attempt to resolve as many as possible of the alerts in all categories. Often the minor alerts point to easily fixed oversights, errors and omissions in your CIF or refinement strategy, so attention to these fine details can be worthwhile. In order to resolve some of the more serious problems it may be necessary to carry out additional measurements or structure refinements. However, the purpose of your study may justify the reported deviations and the more serious of these should normally be commented upon in the discussion or experimental section of a paper or in the "special_details" fields of the CIF. checkCIF was carefully designed to identify outliers and unusual parameters, but every test has its limitations and alerts that are not important in a particular case may appear. Conversely, the absence of alerts does not guarantee there are no aspects of the results needing attention. It is up to the individual to critically assess their own results and, if necessary, seek expert advice.

Publication of your CIF in IUCr journals

A basic structural check has been run on your CIF. These basic checks will be run on all CIFs submitted for publication in IUCr journals (*Acta Crystallographica*, *Journal of Applied Crystallography*, *Journal of Synchrotron Radiation*); however, if you intend to submit to *Acta Crystallographica Section C* or *E* or *IUCrData*, you should make sure that full publication checks are run on the final version of your CIF prior to submission.

Publication of your CIF in other journals

Please refer to the *Notes for Authors* of the relevant journal for any special instructions relating to CIF submission.

PLATON version of 14/11/2023; check.def file version of 14/09/2023

Datablock mc2_81_b_0m_sq - ellipsoid plot

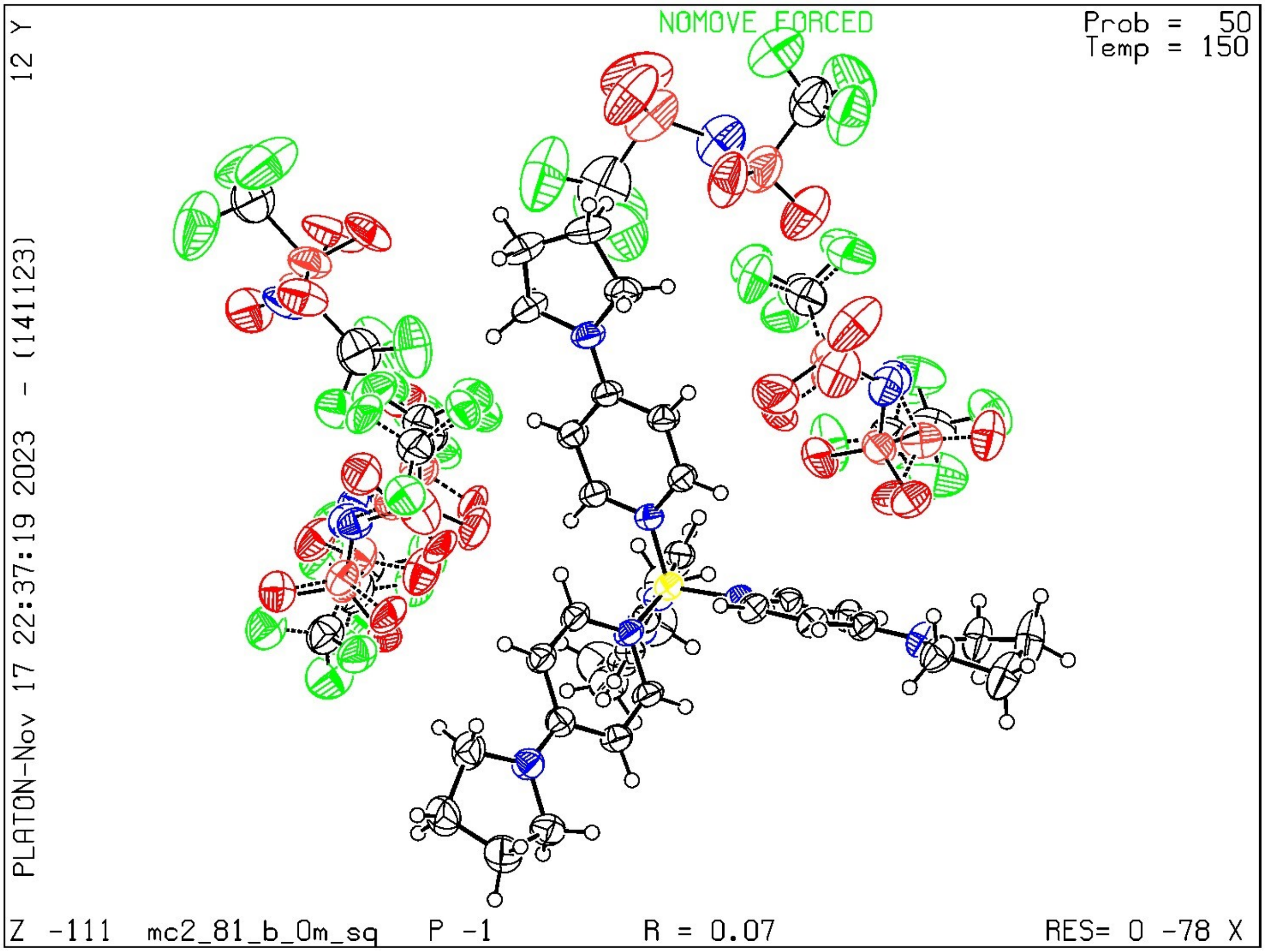

Download CIF editor (publCIF) from the IUCr
Download CIF editor (enCIFer) from the CCDC
Test a new CIF entry
